# The ethoxycarbonyl group as both activating and protective group in *N*-acyl-Pictet–Spengler reactions using methoxystyrenes. A short approach to racemic 1-benzyltetrahydroisoquinoline alkaloids

**DOI:** 10.3762/bjoc.17.183

**Published:** 2021-11-05

**Authors:** Marco Keller, Karl Sauvageot-Witzku, Franz Geisslinger, Nicole Urban, Michael Schaefer, Karin Bartel, Franz Bracher

**Affiliations:** 1 Department of Pharmacy - Center for Drug Research, Ludwig-Maximilians University, Butenandtstr. 5–13, 81377 Munich, Germany; 2 Rudolf-Boehm-Institute for Pharmacology and Toxicology, Härtelstr. 16–18, 04107 Leipzig, Germany

**Keywords:** acyl Pictet–Spengler reaction, alkaloids, antiproliferative activity, benzyltetrahydroisoquinolines, ion channels, protective group, total synthesis

## Abstract

We present a systematic investigation on an improved variant of the *N*-acyl-Pictet–Spengler condensation for the synthesis of 1-benzyltetrahydroisoquinolines, based on our recently published synthesis of *N*-methylcoclaurine, exemplified by the total syntheses of 10 alkaloids in racemic form. Major advantages are a) using ω-methoxystyrenes as convenient alternatives to arylacetaldehydes, and b) using the ethoxycarbonyl residue for both activating the arylethylamine precursors for the cyclization reaction, and, as a significant extension, also as protective group for phenolic residues. After ring closure, the ethoxycarbonyl-protected phenols are deprotected simultaneously with the further processing of the carbamate group, either following route A (lithium alanate reduction) to give *N*-methylated phenolic products, or following route B (treatment with excess methyllithium) to give the corresponding alkaloids with free N–H function. This dual use of the ethoxycarbonyl group shortens the synthetic routes to hydroxylated 1-benzyltetrahydroisoquinolines significantly. Not surprisingly, these ten alkaloids did not show noteworthy effects on TPC2 cation channels and the tumor cell line VCR-R CEM, and did not exhibit P-glycoprotein blocking activity. But due to their free phenolic groups they can serve as valuable intermediates for novel derivatives addressing all of these targets, based on previous evidence for structure–activity relationships in this chemotype.

## Introduction

The benzylisoquinoline alkaloids are a large and diverse class of plant secondary metabolites including about 2,500 known structures. Among these are “simple” benzylisoquinolines and, even more important, their 1,2,3,4-tetrahydro analogues and bisbenzylisoquinolines derived thereof, as well as more complex tetra- and pentacylic ring systems (aporphines, protoberberines, cularines, morphinane-type alkaloids). The common biosynthetic origin of these alkaloids has been investigated thoroughly over decades, and (*S*)-norcoclaurine, a metabolite formally built up by condensation of dopamine and 4-hydroxyphenylacetaldehyde, was identified as the common intermediate. A broad range of biological activities has been reported for benzylisoquinoline alkaloids, including spasmolytic, narcotic, dopaminergic, ion-channel modulating, and cytotoxic properties. Occurrence, biosynthesis and pharmacology of benzylisoquinoline alkaloids has been reviewed comprehensively by Hagel and Facchini [1].

**Figure 1:**
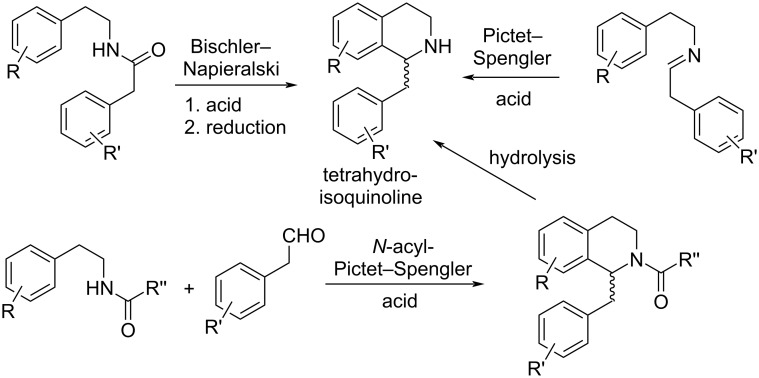
Prominent synthetic approaches to 1-benzyltetrahydroisoquinolines: Bischler–Napieralski, Pictet–Spengler, and *N*-acyl-Pictet–Spengler reactions.

Synthetic approaches to the monomeric 1-benzyl-1,2,3,4-tetrahydroisoquinoline alkaloids are typically inspired by their biosynthesis and comprise Bischler–Napieralski-type cyclizations of arylacetamides (followed by reduction of the resulting 3,4-dihydroisoquinolines) or Pictet–Spengler-type cyclizations of arylacetaldimines of phenylethylamines [2] (Figure 1). Especially the Pictet–Spengler reaction has attracted considerable interest in chemistry and drug development in recent years, and modern methods like enantioselective approaches, organocatalysis, and enzymatic methods have been introduced by numerous groups [3–5].

**Figure 2:**
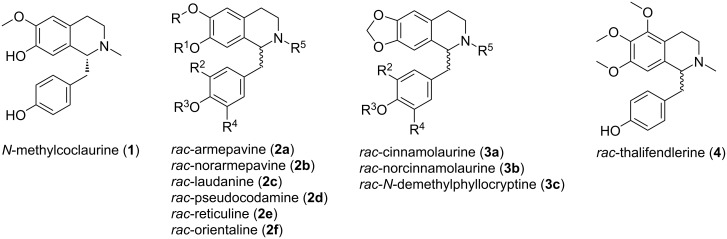
Structures of *N*-methylcoclaurine (**1**) and the ten 1-benzyl-1,2,3,4-tetrahydroisoquinoline alkaloids synthesized (in racemic form) in this investigation.

In continuation of our recent work on the chemistry and pharmacology of benzylisoquinolines and related compounds [6–14] we investigated truncated analogues of the bisbenzylisoquinoline alkaloid tetrandrine as blockers of the calcium channel two-pore channel 2 (TPC2), and identified 1-benzyl-1,2,3,4-tetrahydroisoquinolines bearing phenoxy and benzyloxy substituents (SG-005, SG-094; for structures of bioactive compounds mentioned in this text, see Figure S1 in Supporting Information File 1) on both aromatic rings as potent blockers with promising antitumor activity [15]. In this work we took advantage of the hitherto less explored *N*-acyl-Pictet–Spengler reaction and related chemistry based on the seminal work of Speckamp [16], where an *N*-acyl residue at the arylethylamine building block leads to enhanced cyclization rates due to the acid-mediated formation of highly electrophilic *N*-acyliminium intermediates [17]. As a special aspect, we used a carbamate unit (instead of the commonly used carboxamide), ending up with 1-benzyl-1,2,3,4-tetrahydroisoquinolines bearing an *N*-ethoxycarbonyl residue, which in turn was easily converted directly into an *N*-methyl group by lithium alanate reduction [18–19]. In a novel total synthesis of the racemic alkaloid *N*-methylcoclaurine (**1**) performed in this course we also used the ethoxycarbonyl group successfully for protection of two phenolic groups at two different rings during the *N*-acyl-Pictet–Spengler reaction [15]. This prompted us to investigate the generality of this approach (dual function of the ethoxycarbonyl residue as activating group for the Pictet–Spengler reaction and as protective group for phenolic groups). This effort ended up with short and effective total syntheses of ten racemic 1-benzyl-1,2,3,4-tetrahydroisoquinoline alkaloids (Figure 2 and Table 1). Since, as mentioned above, we recently demonstrated that substituted 1-benzyl-1,2,3,4-tetrahydroisoquinolines exhibit both cytotoxic and calcium channel-modulating activities, we subjected these alkaloids to our established screening systems in order to gain new evidence on structure–activity relationships in this chemotype.

**Table 1:** Substitution patterns of the 1-benzyl-1,2,3,4-tetrahydroisoquinoline alkaloids **2a**–**f** and **3a**–**c** shown in Figure 2.

Compound	R	R^1^	R^2^	R^3^	R^4^	R^5^

**2a**	CH_3_	CH_3_	H	H	H	CH_3_
**2b**	CH_3_	CH_3_	H	H	H	H
**2c**	CH_3_	CH_3_	OH	CH_3_	H	CH_3_
**2d**	CH_3_	CH_3_	H	H	OCH_3_	CH_3_
**2e**	CH_3_	H	OH	CH_3_	H	CH_3_
**2f**	CH_3_	H	H	H	OCH_3_	CH_3_
**3a**	–	–	H	H	H	CH_3_
**3b**	–	–	H	H	H	H
**3c**	–	–	OH	CH_3_	H	CH_3_

## Results and Discussion

**Figure 3:**
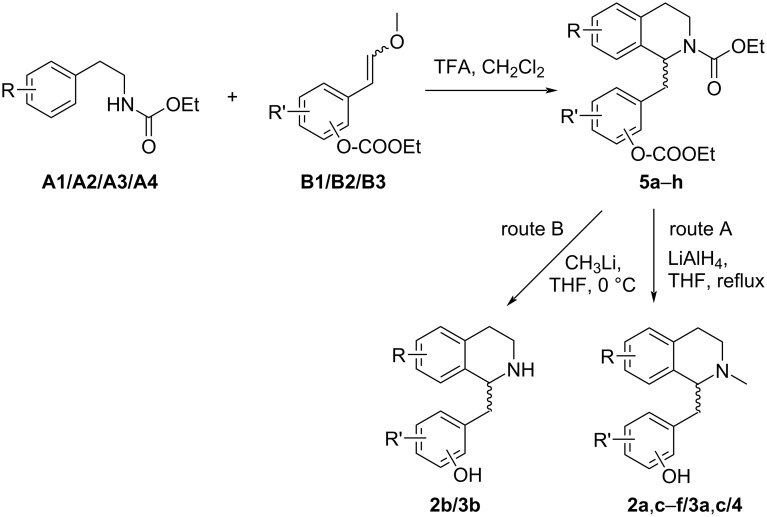
Two routes using *N*- and *O*-alkoxycarbonylated building blocks for the synthesis of phenolic *N*-methyl 1-benzyl-1,2,3,4-tetrahydroisoquinoline alkaloids (route A) and their N–H congeners (route B).

Encouraged by scattered reports on *N*-acyl-Pictet–Spengler reactions using *N*-alkoxycarbonyl activation [10,15,18–21] (noteworthy, *N*-Boc is not reliably resistant to the strongly acidic cyclization conditions [22–23]) we further intended to replace the unstable and poorly accessible arylacetaldehyde building blocks [19,22] by more common equivalents, and ended up with ω-methoxystyrenes [18], which have been demonstrated to be advantageous arylacetaldehyde equivalents in strongly acidic, anhydrous media, used for *N*-acyl-Pictet–Spengler [18] and Povarov [23] reactions before. In order to avoid tedious protective group manipulations in the synthesis of alkaloids bearing free phenolic groups (e.g., with commonly used isopropoxy [19], TIPS or benzyl [24], or benzoyl [25] protection) we investigated the utilization of the ethoxycarbonyl group for phenol protection. The main objective of this concept was, that after successful *N*-acyl-Pictet–Spengler cyclization two remaining pairs of transformations might be performed in one single transformation each: route A comprises reduction with lithium alanate, and should lead to an *N*-methyl group and to reductive cleavage of the carbonate-protected phenol(s); route B is based on treatment with an alkyllithium compound [26], which should remove all ethoxycarbonyl groups and provide *N*-nor analogues of the products obtained in route A (Figure 3).

**Figure 4:**
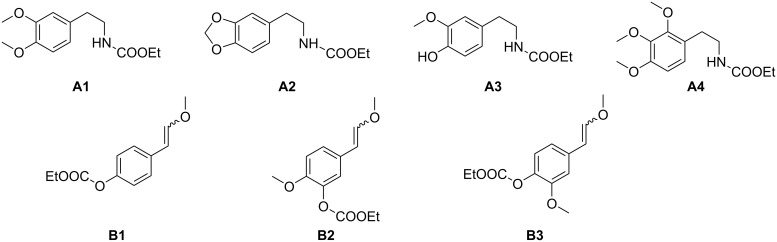
Structures of the building blocks **A1**–**A4** (*N*-ethoxycarbonyl phenethylamines) and **B1**–**B3** (ω-methoxystyrenes).

The required building blocks (ring-substituted *N*-ethoxycarbonyl phenethylamines **A** and ω-methoxystyrenes **B**) were, if not commercially available, prepared from appropriately substituted benzaldehydes (see Supporting Information File 1). For the ω-methoxystyrenes, phenolic groups in the benzaldehydes were protected using ethyl chloroformate/Et_3_N, followed by Wittig olefination with an ylide generated from (methoxymethyl)triphenylphosphonium chloride and LDA to give the enol ethers as *E*/*Z* mixtures. *N*-Phenethyl carbamates were obtained from benzaldehydes via Henry reaction with nitromethane, followed by zinc dust reduction of the intermediate nitrostyrenes and *N*-ethoxycarbonylation of the resulting primary amines [15]. For the synthesis of the alkaloids *rac*-reticuline (**2e**) and *rac*-orientaline (**2f**) we used a carbamate building block **A3** without protection of the phenolic group, since our previous work [10] demonstrated that this building block is compatible with *N*-acyl-Pictet–Spengler cyclization conditions. The structures of the utilized building blocks are shown in Figure 4.

The *N*-acyl-Pictet–Spengler cyclizations of appropriate pairs of the building blocks with TFA in dichloromethane at 0 °C for 19–90 h (TLC control) gave the desired racemic *N*-ethoxycarbonyl-1-benzyltetrahydroisoquinolines **5a**–**h** with intact ethoxycarbonyl protection of the phenolic groups (Table 2). Simultaneous phenol deprotection and reduction of the carbamate group (route A) to an *N*-methyl group was accomplished by lithium alanate reduction in THF under reflux to give the racemic forms of the alkaloids armepavine (**2a**; from *Rhamnus frangula* [27–29]), laudanine (**2c**; from *Papaver somniferum* [30–31]), pseudocodamine (**2d**; metabolite of isoorientaline in *Corydalis platycarpa makino* cell species [32]), reticuline (**2e**; from *Papaver somniferum* [33]), orientaline (**2f**; from *Cryptocarya amygdalina* [34–35]), cinnamolaurine (**3a**; from *Cinnamomum sp. T.G.H. 13077* [36]), *N*-demethylphyllocryptine (**3c;** from *Cryptocarya phyllosternon* [37–38]) and thalifendlerine (**4**; from *Thalictrum fendleri* [39–40]) in yields ranging from 36 to 82%. The *N*-nor-alkaloids *rac*-norarmepavine (**2b**; from *Nelumbo nucifer* [41]) and *rac*-norcinnamolaurine (**3b**; from *Cinnamomum sp. T.G.H. 13077* [36]) were obtained in moderate yields following route B, in which ethoxycarbonyl groups from both nitrogen and oxygen were removed by treatment with excess methyllithium in THF at 0 °C for 1 h (Table 2). Spectroscopic data of the racemic products were in full agreement with published data from former total syntheses [24–25,36,38,42–45].

**Table 2:** Two-step synthesis of the racemic 1-benzyl-1,2,3,4-tetrahydroisoquinoline alkaloids starting from building blocks of types **A** and **B** (for details, see Figure 3 and Figure 4).

Building blocks combined	Intermediate (yield)	Route	Alkaloid (yield), previous total synthesis (selection)

**A1** + **B1**	**5a** (77%)	A	*rac*-armepavine (**2a**, 62%) [24]
**A1** + **B1**	**5a** (77%)	B	*rac*-norarmepavine (**2b**, 35%) [42]
**A1** + **B2**	**5b** (33%)	A	*rac*-laudanine (**2c**, 48%) [25]
**A1** + **B3**	**5c** (46%)	A	*rac*-pseudocodamine (**2d**, 69%) [25]
**A3** + **B2**	**5d** (35%)	A	*rac*-reticuline (**2e**, 63%) [43]
**A3** + **B3**	**5e** (52%)	A	*rac*-orientaline (**2f**, 62%) [45]
**A2** + **B1**	**5f** (53%)	A	*rac*-cinnamolaurine (**3a**, 67%) [36]
**A2** + **B1**	**5f** (53%)	B	*rac*-norcinnamolaurine (**3b**, 44%) [36]
**A2** + **B2**	**5g** (31%)	A	*rac*-*N*-demethylphyllocryptine (**3c**, 36%) [38]
**A4** + **B1**	**5h** (44%)	A	*rac*-thalifendlerine (**4**, 82%) [44]

## Screening

Bisbenzylisoquinoline alkaloids like tetrandrine and cepharanthine exhibit a broad spectrum of antimicrobial activities, antitumor, and antiviral effects [15]. Mechanistically, they have been shown to block two pore channel 2 (TPC2), an endolysosomal cation channel which is important for cellular migration and survival [6,15,46–47]. In our recent search for TPC2 blockers with antiproliferative potential we identified ring-substituted 1-benzyl-1,2,3,4-tetrahydroisoquinolines (SG-005 and SG-094) as potent lead structures. For activity on TPC2 as well as tumor cells, these compounds were preferably bearing phenoxy and benzyloxy substituents on both aromatic rings, however, some analogues missing this “aromatic decoration” retained antiproliferative activity, while losing activity on TPC2 [15]. This prompted us to analyze both TPC2 modulating and antiproliferative activities of the alkaloids from this project.

### TPC2 modulation

To assess the activity of racemic 1-benzyl-1,2,3,4-tetrahydroisoquinoline alkaloids on TPC2 in comparison to tetrandrine and cepharanthine, we performed Ca^2+^ measurements in multiwell plates. Therefore, heterologously TPC2-expressing HEK293 cells were loaded with calcium indicator fluo-4/AM and Ca^2+^ entry was measured for 5 min after injection of various alkaloid concentrations (0.1–100 µM). None of the alkaloids exhibited the efficiency to activate TPC2 (data not shown). To evaluate the inhibitory potency, afterwards the same cells were stimulated with the TPC2 activators TPC2-A1-P (30 µM) and TPC2-A1-N (10 µM). Compared with the respective solvent control (DMSO), only *rac*-norcinnamolaurine (**3b**) showed a weak TPC2 inhibition at 100 µM. The nine other alkaloids did not significantly inhibit human TPC2, whereas tetrandrine and cepharanthine concentration-dependently blocked with IC_50_ values of 31/21 µM and 3.6/57 µM (Figure S2A and Figure S2B in Supporting Information File 1). Note that TPC2 activation by TPC2-A1-P is better targetable than by TPC2-A1-N.

This result is in accordance with our previous observations that TPC2-blocking activity is only observed for monomeric benzyltetrahydroisoquinolines if they are decorated with aromatic residues, which imitate the two benzenoid rings of the second benzylisoquinoline moiety of the bisbenzylisoquinolines [15].

### Antiproliferative activity

Several anticancer effects have been reported for benzylisoquinoline alkaloids, for instance antiproliferative and antimigratory as well as chemoresistance-reversing activities [6,15,46–47]. Yet, detailed data on structure–activity relationship are still rare.

**Figure 5:**
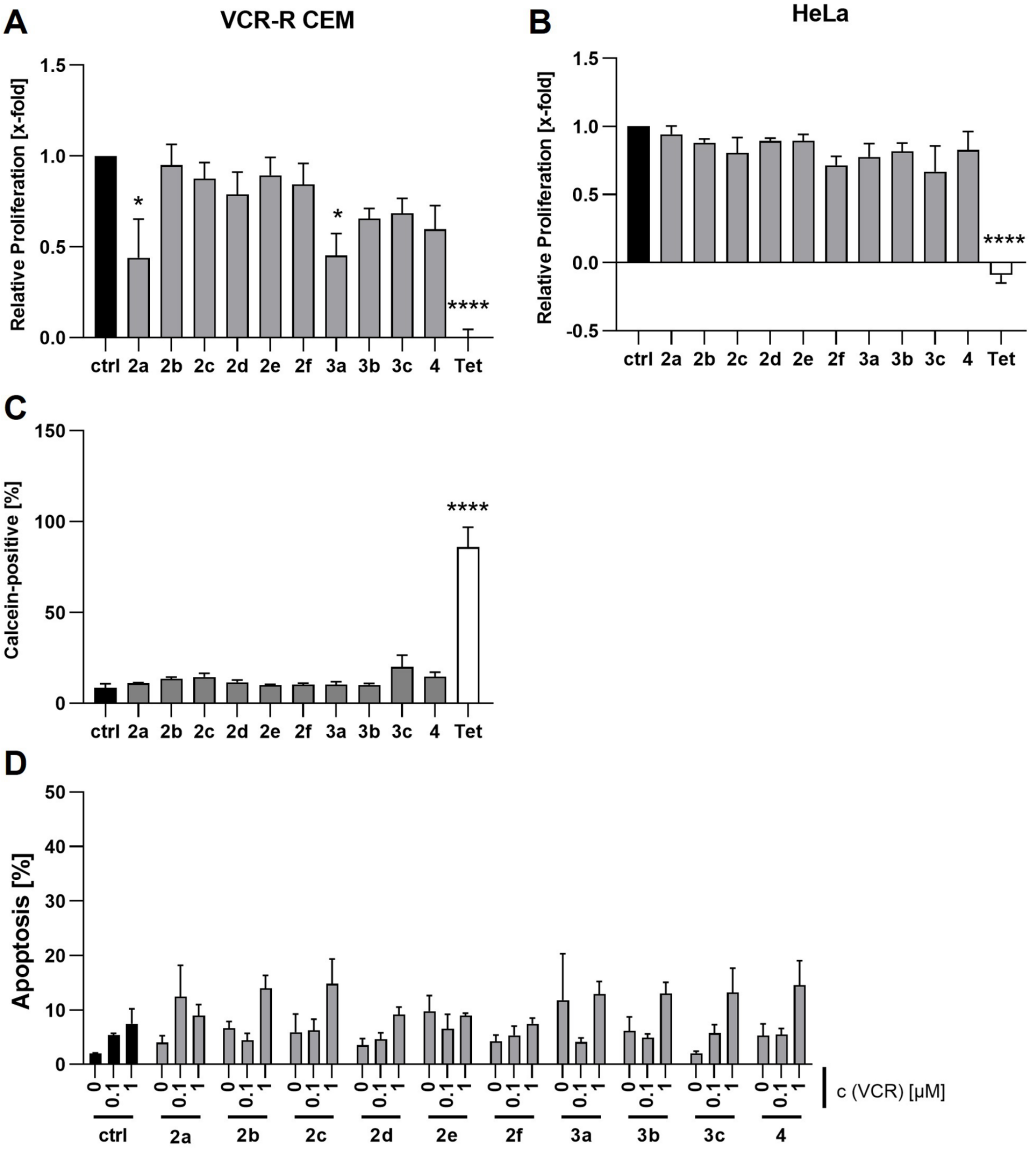
Biological activity. Antiproliferative effects of the 1-benzyltetrahydroisoquinoline alkaloids in A) HeLa and B) VCR-R CEM cells. Cells were treated with the respective compound (50 µM) for 72 h and relative proliferation was assessed by CellTiter Blue assay. Statistical significance was determined by ordinary one-way ANOVA and Dunnett’s post test. *p < 0.05; ****p < 0.0001. C) Influence of benzylisoquinolines on the retention of the P-glycoprotein substrate calcein-AM. VCR-R CEM cells were treated with the respective compounds (10 µM) for 30 min in presence of calcein-AM (200 nM) and 60 min without calcein-AM and analyzed by flow cytometry. Cells were gated for calcein-positive events. Statistical significance was determined by ordinary one-way ANOVA and Dunnett’s post test. ****p < 0.0001. D) Combination treatment of benzylisoquinolines and vincristine. VCR-R CEM cells were treated with 0.1 or 1 µM vincristine for 48 h in presence or absence of the respective benzylisoquinoline (10 µM). Apoptosis was determined by propidium iodide staining and flow cytometry. Statistical significance was determined by two-way ANOVA and Dunnett’s post test (no significant changes for any combination).

First, we assessed the antiproliferative activity of the benzylisoquinolines **2a**–**f**, **3a**–**c** and **4** by CellTiter Blue cell viability assay and used tetrandrine as reference compound. In the cervical cancer cell line HeLa, none of the newly tested compounds showed antiproliferative effects, in contrast to tetrandrine which potently inhibits cancer cell proliferation. In the multidrug resistant leukemia cell line VCR-R CEM proliferation was measurably reduced upon treatment with two of the investigated compounds, *rac*-armepavine (**2a**) and *rac*-cinnamolaurine (**3a**), however, to a significantly lesser extent (IC_50_ values about 40 µM; see Figure S3 in Supporting Information File 1) as compared to tetrandrine treatment (IC_50_ = 4 µM). The other tested compounds showed again no activity (Figure 5A and Figure 5B). The alkaloids with measurable antiproliferative effect from this and a previous investigation (*N*-methylcoclaurine (**1**)) have in common a 4-hydroxybenzyl residue at C-1.

These results indicate that the „aromatic decoration“ of benzylisoquinolines (phenoxy or benzyloxy residues on both aromatic rings) [15] is also important for antiproliferative activity. This is supported by the fact that the alkaloids investigated here bearing only hydroxy and small alkoxy residues, show no inhibitory effect on TPC2 as well. Our results further suggest that antiproliferative activity of benzylisoquinolines is connected to their inhibitory potency on TPC2.

Interestingly, macrocyclic bisbenzylisoquinolines such as tetrandrine [48–49] and related alkaloids [50], have been reported to inhibit P‐glycoprotein (P-gp), a crucial factor of multidrug resistance in cancer. This is noteworthy as multidrug resistance is still a major problem in cancer therapy and no approved P-gp inhibitors are available in clinics [50]. Further, structurally related molecules, like the *seco*-analogues dauricine and daurisoline and the truncated bisbenzylisoquinoline muraricine (Supporting Information File 1, Figure S1) also show [51] P-gp inhibitory potential, so we investigated the compounds **2a**–**f**, **3a**–**c** and **4** regarding their P-glycoprotein blocking potential. For that, we used the P-glycoprotein substrate calcein as reporter dye and analyzed intracellular calcein fluorescence of P-glycoprotein overexpressing VCR-R CEM cells by flow cytometry. An increase of the calcein-positive population indicates inhibition of P-glycoprotein. However, none of the tested compounds was able to retain calcein, indicating no P-glycoprotein inhibition. Tetrandrine served as positive control (Figure 5C). In line with that, none of the compounds restored sensitivity of VCR-R CEM cells to the P-glycoprotein substrate vincristine in a combination treatment of benzylisoquinoline alkaloids and vincristine (Figure 5D).

Taken together, our data clearly indicate that „aromatic decoration“, as present in SG-005 and SG-094 (Figure S1 in Supporting Information File 1), is indispensable for strong biological effects of benzyltetrahydroisoquinolines, including modulation of TPC2 and anticancer effects, as natural benzyltetrahydroisoquinolines bearing only hydroxy, methoxy and/or methylenedioxy substituents show no significant activity in this regard.

## Conclusion

In the present investigation we demonstrated the broad utility of a variant of the *N*-acyl-Pictet–Spengler condensation for the synthesis of 1-benzyltetrahydroisoquinolines using easily accessible and stable building blocks (*N*-ethoxycarbonyl phenethylamines and ω-methoxystyrenes). Particularly, we showed that the ethoxycarbonyl group is very advantageous for the construction of the tetrahydroisoquinoline moiety, since on the one side it enables cyclization using the effective *N*-acyl-Pictet–Spengler methodology, and on the other side it can, in the final step, be optionally converted into a *N*-methyl (route A) or a NH group (route B). And finally, the ethoxycarbonyl group can as well be utilized for the protection of phenolic groups, which frequently occur in 1-benzyltetrahydroisoquinoline alkaloids. After successful ring closure, the ethoxycarbonyl-protected phenols are deprotected simultaneously with the further processing of the carbamate group following routes A or B, thus reducing the required number of steps for alkaloid total syntheses significantly. In this way we worked out convenient racemic total syntheses of ten 1-benzyltetrahydroisoquinoline alkaloids. Formally, this approach also opens the option for the preparation of enantiomerically pure alkaloids, as numerous methods for separation of enantiomers are well known in this field [52–55]. Further, this protocol might be extended to an asymmetric *N*-acyl-Pictet–Spengler condensation by using homochiral carbamates, as demonstrated by Comins [18], or by using enantioselective organocatalysis based on Jacobsen’s pioneering work [56].

Since we had recently identified substituted 1-benzyltetrahydroisoquinolines as truncated analogues of bioactive bisbenzylisoquinoline alkaloids (SG-005 and SG-094) as TPC2 blockers with antiproliferative activity, we tested our alkaloids for effects on cation channels and the VCR-R CEM tumor cell line. As expected, the ten alkaloids did not block the cation channel TPC2, and this result confirms former evidence on structure–activity relationships on this target. In line with that, testing for biological activities of the novel benzylisoquinolines **2a**–**f**, **3a-c** and **4** revealed that they possess neither antiproliferative nor P-glycoprotein blocking activity.

Nevertheless, the results of this investigation represent a considerable contribution to the further development of benzyltetrahydroisoquinoline-derived bioactive compounds, since the here developed convenient access to phenolic benzyltetrahydroisoquinolines opens the possibility for flexible late stage etherification with various aromatic, benzylic and related residues for a comprehensive investigation of the chemical space around this pharmacophoric backbone.

## Experimental

### Materials and methods

All chemicals used were of analytical grade and were obtained from abcr (Karlsruhe, Germany), Fischer Scientific (Schwerte, Germany), Merck, Darmstadt, Germany, TCI (Eschborn, Germany) or Th. Geyer (Renningen, Germany). HPLC grade and dry solvents were purchased from VWR (Darmstadt, Germany) or Merck (Darmstadt, Germany), all other solvents were purified by distillation. All reactions were monitored by thin-layer chromatography (TLC) using precoated plastic sheets POLYGRAM^®^ SIL G/UV254 from Macherey-Nagel and detected by irradiation with UV light (254 nm). Flash column chromatography (FCC) was performed on Merck silica gel Si 60 (0.015–0.040 mm).

NMR spectra (^1^H, ^13^C, DEPT, H,H-COSY, HSQC, HMQC, HMBC) were recorded at 23 °C on an Avance III 400 MHz Bruker BioSpin or Avance III 500 MHz Bruker BioSpin instrument. Chemical shifts δ are stated in parts per million (ppm) and are calibrated using residual protic solvent as an internal reference for proton (CDCl_3_: δ = 7.26 ppm, methanol-*d*_4_: δ = 3.31 ppm, C_2_D_2_Cl_4_: δ = 5.91 ppm, DMSO-*d*_6_: δ = 2.50 ppm) and for carbon the central carbon resonance of the solvent (CDCl_3_: δ = 77.2 ppm, methanol-*d*_4_: δ = 49.0 ppm, C_2_D_2_Cl_4_: δ = 74.2 ppm, DMSO-*d*_6_: δ = 39.5 ppm). Multiplicity is defined as s = singlet, d = doublet, t = triplet, q = quartet, p = pentet, m = multiplet. NMR spectra were analyzed with NMR software MestReNova, version 14.2.0-26256 (Mestrelab Research S.L.). High-resolution mass spectra were performed by the LMU Mass Spectrometry Service applying a Thermo Finnigan LTQ FT Ultra Fourier Transform Ion Cyclotron Resonance device at 250 °C for ESI. IR spectra were recorded on a Perkin Elmer FT-IR Paragon 1000 instrument as neat materials. Absorption bands were reported in wave numbers (cm^−1^), obtained on a ATR PRO450-S accessory (Jasco). Melting points were determined by the open tube capillary method on a Büchi melting point B-540 apparatus and are uncorrected. HPLC purities were determined using an HP Agilent 1100 HPLC with a diode array detector and an Agilent Zorbax Eclipse plus C18 column (150 × 4.6 mm; 5 µm) with methanol/water in different proportions adjusted to pH 9 with NaOH or neutral as mobile phase.

Details of the syntheses and spectroscopic data are presented in Supporting Information File 1.

### Fluorometric Ca^2+^ measurements

Concentration–response curves of 1-benzyltetrahydroisoquinolines were generated by using a custom-made fluorescence imaging plate reader (FLIPR) built into a robotic liquid handling station (Fluent, Tecan, Switzerland) and a HEK293 cell line stably expressing the human TPC2 (C-terminally RFP-tagged) rerouted to the plasma membrane as previously described [57]. Trypsinized HEK293 cells (stably expressing hTPC2L11A/L12A*)* suspensions were loaded with 4 µM fluo-4/AM (Life Technologies, Eugene, OR) for 30 min at 37 °C. After centrifugation, cells were resuspended in HEPES-buffered saline (HBS), containing 132 mM NaCl, 6 mM KCl, 1 mM MgCl_2_, 1 mM CaCl_2_, 10 mM HEPES, and 5.5 mM ᴅ-glucose (pH was adjusted to 7.4 with NaOH) and dispensed into black pigmented, clear-bottom 384-well plates (Greiner, Germany). Then, plates were mounted on the FLIPR, fluo-4 was excited by LED array and emitted light was imaged through a 515 nm long pass filter with a Zyla 5.5 camera (Andor, Belfast, UK) under control of the Micromanager software. After recording a baseline for 60 s, serially prediluted compounds were added with a 384-tip multichannel arm (MCA384, Tecan) at the indicated concentrations and incubated for 5 min. In a second step, all 384 wells were stimulated with 10 µM TPC2-A1-N or 30 µM TPC2-A1-P. Finally, fluorescence intensities in each single well was calculated with ImageJ software, corrected for the respective background signals and normalized to the initial fluorescence intensities (F/F_0_). Concentration–response curves were generated by fitting the data to a four-parameter Hill equation (*E*_min_, *E*_max_, EC_50_/IC_50_, and Hill coefficient *n*).

### Antiproliferative acitvity

#### Cell lines and culture

HeLa cells were purchased from German Research Centre of Biological Material (DSMZ) and cultured in DMEM (PAN Biotech) supplemented with 10% FCS (PAA Laboratories). VCR-R CEM cells were a kind gift from Prof. Maria Kavallaris (University of New South Wales) [58] and cultured in RPMI-1640 (PAN Biotech) supplemented with 10% FCS (PAA Laboratories). Cells were cultured at 37 °C with high humidity and 5% CO_2_.

#### Cell proliferation assays

HeLa (5 × 10^3^ cells/well) or VCR-R CEM cells (20 × 10^3^ cells/well) were plated in 96-well plates and stimulated as indicated for 72 h. Two hours before termination, CellTiter Blue reagent (Promega) was added according to manufacturer’s instructions. Fluorescence intensity was measured at 590 nm on a Sunrise ELISA reader (TECAN) and is proportional to the cell number. Relative proliferation was calculated after substraction of the basal value.

#### Flow cytometry

P-glycoprotein activity was assessed using the reporter dye calcein acetoxymethyl ester (AM) as described previously [59].

To assess apoptosis, VCR-R CEM cells were seeded at 0.125 × 10^6^ cells/well, incubated for 4 h and stimulated as indicated for 48 h. Apoptosis was analyzed by propidium iodide staining and flow cytometry as described by Nicoletti et al. [60]. All flow cytometry experiments were conducted on a BD FACS Canto II (BD Biosciences).

## Supporting Information

File 1 Alkaloid structures, experimental, copies of spectra, and biological screening.
