# Supplementary material for: The ethoxycarbonyl group as both activating and protective group in N-acyl-Pictet–Spengler reactions using methoxystyrenes. A short approach to racemic 1-benzyltetrahydroisoquinoline alkaloids
Source: Beilstein J Org Chem. 2021 Nov 5;17:2716–25. doi: 10.3762/bjoc.17.183 (PMC8576818; doi:10.3762/bjoc.17.183)
Supplement: File 1 — Alkaloid structures, experimental, copies of spectra, and biological screening. [file Beilstein_J_Org_Chem-17-2716-s001.pdf]

BEILSTEIN JOURNAL OF ORGANIC CHEMISTRY

Supporting Information

for

The ethoxycarbonyl group as both activating and protective group in *N*-acyl-Pictet–Spengler reactions using methoxystyrenes. A short approach to racemic 1-benzyltetrahydroisoquinoline alkaloids

Marco Keller, Karl Sauvageot-Witzku, Franz Geisslinger, Nicole Urban, Michael Schaefer, Karin Bartel and Franz Bracher

Beilstein J. Org. Chem. **2021,** *17,* 2716–2725. doi:10.3762/bjoc.17.183

Alkaloid structures, experimental, copies of spectra, and biological screening

License and Terms: This is a supporting information file under the terms of the Creative Commons Attribution License (https://creativecommons.org/licenses/by/4.0). Please note that the reuse, redistribution and reproduction in particular requires that the author(s) and source are credited and that individual graphics may be subject to special legal provisions.
The license is subject to the *Beilstein Journal of Organic Chemistry* terms and conditions: (https://www.beilstein-journals.org/bjoc/terms)

Table of contents

1. Structures of bioactive alkaloids and their analogues

2. Chemistry: General procedures and details on the synthesis of precursors

3. ^{1}H and ^{13}C NMR spectra

4. Screening for TPC2 modulation

5. Screening for antiproliferative activity of selected alkaloids

References

1. Structures of bioactive alkaloids and their analogues

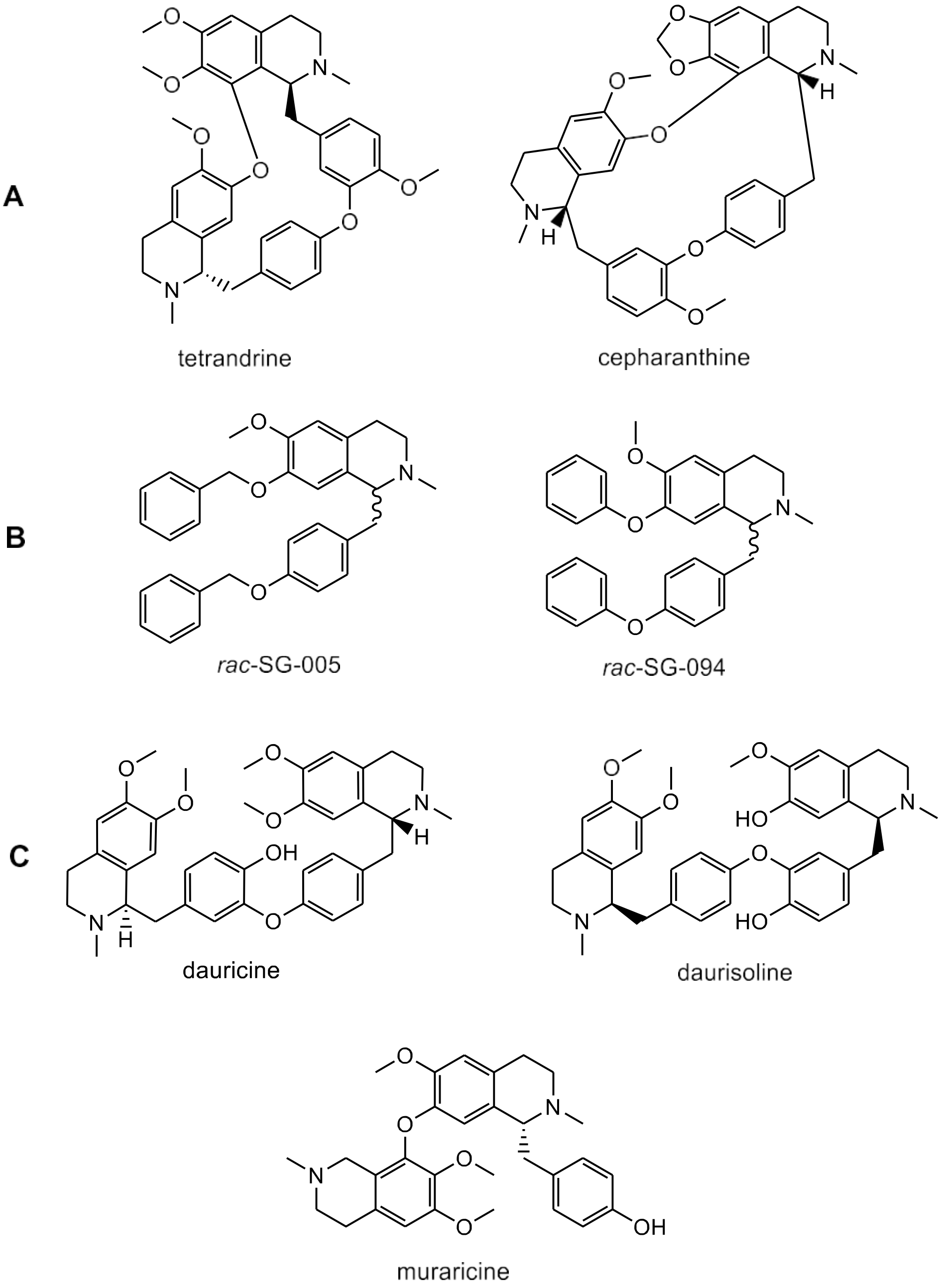

Figure S1 Structures of bioactive alkaloids and their analogues mentioned in the manuscript:

A: bisbenzylisoquinoline alkaloids **tetrandrine**, **cepharanthine**

B: synthetic, truncated analogues of tetrandrine: **SG-005**, **SG-094**

C: seco-bisbenzylisoquinoline alkaloids: **dauricine**, **daurisoline**, **muraricine**

2. Chemistry

All chemicals used were of analytical grade and were obtained from abcr (Karlsruhe, Germany), Fischer Scientific (Schwerte, Germany), Merck, Darmstadt, Germany, TCI (Eschborn, Germany) or Th. Geyer (Renningen, Germany). HPLC grade and dry solvents were purchased from VWR (Darmstadt, Germany) or Merck (Darmstadt, Germany), all other solvents were purified by distillation. All reactions were monitored by thin-layer chromatography (TLC) using pre-coated plastic sheets POLYGRAM® SIL G/UV254 from Macherey-Nagel and detected by irradiation with UV light (254 nm). Flash column chromatography (FCC) was performed on Merck silica gel Si 60 (0.015–0.040 mm).

NMR spectra (^{1}H, ^{13}C, DEPT, H-H-COSY, HSQC/HMQC, HMBC) were recorded at 23 °C on an Avance III 400 MHz Bruker BioSpin or Avance III 500 MHz Bruker BioSpin instrument. Chemical shifts δ are stated in parts per million (ppm) and are calibrated using residual protic solvent as an internal reference for proton ($CDCl_3$: δ = 7.26 ppm, methanol-d_4: δ = 3.31 ppm, $C_2D_2Cl_4$: δ = 5.91 ppm, DMSO-d_6: δ = 2.50 ppm) and for carbon the central carbon resonance of the solvent ($CDCl_3$: δ = 77.2 ppm, methanol-d_4: δ = 49.0 ppm, $C_2D_2Cl_4$: δ =74.2 ppm, DMSO-d_6: δ = 39.5 ppm). Multiplicity is defined as s = singlet, d = doublet, t = triplet, q = quartet, p = pentet, m = multiplet. NMR spectra were analysed with NMR software MestReNova, version 14.2.0-26256 (Mestrelab Research S.L.). High resolution mass spectra were performed by the LMU Mass Spectrometry Service applying a Thermo Finnigan LTQ FT Ultra Fourier Transform Ion Cyclotron Resonance device at 250 °C for ESI. IR spectra were recorded on a Perkin Elmer FT-IR Paragon 1000 instrument as neat materials. Absorption bands were reported in wave number (cm^{-1}) with ATR PRO450-S. Melting points were determined by the open tube capillary method on a Büchi melting point B-540 apparatus and are uncorrected. HPLC purities were determined using an HP Agilent 1100 HPLC with a diode array detector and an Agilent Zorbax Eclipse plus C18 column (150 × 4.6 mm; 5 µm) with methanol/water in different proportions adjusted to pH 9 with NaOH or neutral as mobile phase.

Synthesis details and analytical data:

General Procedure A – Protection with an ethoxycarbonyl group

In a manner similar to a procedure in [1], the appropriate arylethylamine or phenol (1.0 equiv) was dispersed/dissolved in dry CH_2Cl_2 and cooled to −20 °C, followed by dropwise addition of NEt_3 (1.5–3.5 equiv) and ethyl chloroformate (1.3–2.5 equiv). The reaction mixture was refluxed for 4–72 h, before water was added. The mixture was extracted thrice with CH_2Cl_2, the combined organic layers were dried over Na_2SO_4 and concentrated *in vacuo.* Purification was accomplished by flash column chromatography (FCC) or recrystallization to yield carbamates and carbonates.

General Procedure B – Wittig reaction

In a manner similar to a procedure in [1], (methoxymethyl)triphenylphosphonium chloride (1.2 equiv) was suspended in dry THF under nitrogen and cooled to −4 °C, before LDA (1 equiv) was added dropwise. The mixture was stirred for 30 min at −4 °C, followed by slow, dropwise addition of a solution of the appropriate aldehyde (1.0 equiv) in dry THF. The solution was stirred for 2 h at 0 °C, then poured on brine and extracted thrice with CH_2Cl_2. The combined organic layers were dried over Na_2SO_4, filtered and concentrated *in vacuo*. The crude product was purified by FCC to give desired *cis*/*trans*-enol ethers. Due to limited stability, the products are stored at −18 °C.

General Procedure C – *N*-Acyl Pictet–Spengler reaction

In a manner similar to a procedure in [1], carbamate (1.0 equiv) and enol ether (1.0–1.2 equiv) were dissolved in dry dichloromethane, 4 Å molecular sieves (1.00 g per 20 mL solvent) was added and the mixture was cooled to 0 °C, before TFA (10–11 equiv) was added dropwise. The mixture was allowed to warm to rt and stirred for 18–90 h, before the molecular sieves was removed by filtration. Saturated aqueous $NaHCO_3$ solution was added. The mixture was extracted thrice with CH_2Cl_2, the combined organic layers were washed with 10% aqueous citric acid and brine, dried over Na_2SO_4, filtered and concentrated *in vacuo*. Purification was accomplished by FCC or recrystallization to yield desired racemic tetrahydroisoquinolines.

General Procedure D – Lithium alanate reduction

In a manner similar to a procedure in [1], $LiAlH_4$ (7–12 equiv) was suspended under nitrogen in 8 mL dry THF and a solution of the carbamate (1.0 equiv) in 8 mL dry THF

was added dropwise, and the suspension was refluxed for 3–20 h. The mixture was cooled to 0 °C and a saturated solution of Na_2SO_4 was added in small portions under vigorous stirring over 30 min. Water was added and the mixture was extracted thrice with CH_2Cl_2. The combined organic layers were dried over Na_2SO_4 and concentrated *in vacuo*. Purification was accomplished by FCC to give tertiary amines.

Preparation of additional compounds (precursors)

(*E*)-1,2,3-Trimethoxy-4-(2-nitrovinyl)benzene

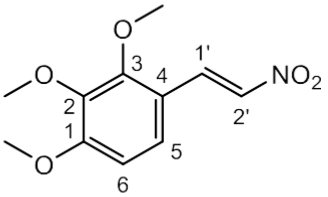

Synthesis and characterization of (*E*)-1,2,3-trimethoxy-4-(2-nitrovinyl)benzene was reported previously in [2].

Ethyl (5-formyl-2-methoxyphenyl) carbonate

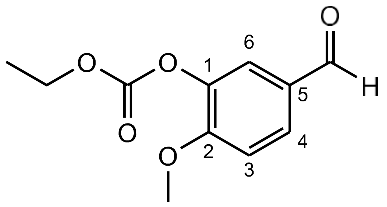

Following general procedure A, 3-hydroxy-4-methoxy-benzaldehyde (2.00 g, 13.1 mmol, 1.0 equiv) in dry CH_2Cl_2 (10 mL), NEt_3 (2.75 mL, 19.7 mmol, 1.5 equiv) and ethyl chloroformate (1.63 mL, 17.1 mmol, 1.3 equiv) were used. The reaction was completed after 14 h and the product was purified by recrystallization from ethyl acetate and hexanes to yield the desired aldehyde (2.78 g, 13.1 mmol, 95%) as a colorless solid. m.p.: 61.1 °C; ^{1}H NMR (400 MHz, $CDCl_3$): δ (ppm) = 9.87 (s, 1H, CHO), 7.78 (dd, *J* = 8.5, 2.0 Hz, 1H, 4-H), 7.68 (d, *J* = 2.0 Hz, 1H, 6-H), 7.09 (d, *J* = 8.5 Hz, 1H, 3-H), 4.33 (q, *J* = 7.1 Hz, 2H, $C\underline{H}_2CH_3$), 3.95 (s, 3H, OCH_3), 1.40 (t, *J* = 7.1 Hz, 3H, $CH_2C\underline{H}_3$); ^{13}C NMR (101 MHz, $CDCl_3$): δ (ppm) = 190.1 (CHO), 156.5 (C-2), 153.0 (CO), 140.7 (C-1), 130.3 (C-7), 130.1 (C-5), 123.3 (C-6), 112.3 (C-3), 65.4 (CH_2), 56.5 (OCH_3), 14.3 (CH_3); IR (ATR) $\tilde{\nu}_{max}/cm^{-1}$ = 1753, 1689, 1603, 1512, 1254,

1210, 1123, 1055, 1021,999, 941, 901, 827, 791, 764; HRMS (EI): calcd. for $C_{11}H_{12}O_5$ 225.0763 $[M+H]^+$; found 225.0761; purity (HPLC): >97%.

Ethyl (4-formyl-2-methoxyphenyl) carbonate

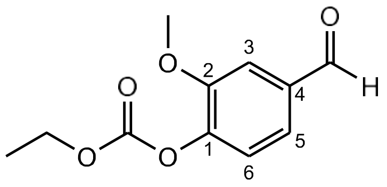

Synthesis and characterization of ethyl (4-formyl-2-methoxyphenyl) carbonate was reported previously in [3]. The synthesis was adapted and the missing NMR data is reported. Following general procedure A, 4-hydroxy-3-methoxybenzaldehyde (2.00 g, 13.1 mmol, 1.0 equiv) in dry CH_2Cl_2 (10 mL), NEt_3 (2.75 mL, 19.7 mmol, 1.5 equiv) and ethyl chloroformate (1.63 mL, 17.1 mmol, 1.3 equiv) were used. The reaction was completed after 14 h and the product was purified by recrystallization from ethyl acetate and hexanes to yield the desired aldehyde (2.55 g, 11.4 mmol, 87%) as a colorless solid. m.p.: 73.1 °C; ^{1}H NMR (400 MHz, $CDCl_3$): δ (ppm) = 9.95 (s, 1H, CHO), 7.51 (d, J = 1.8 Hz, 1H, 3-H), 7.48 (dd, J = 8.0, 1.8 Hz, 1H, 5-H), 7.32 (d, J = 8.0 Hz, 1H, 6-H), 4.33 (q, J = 7.1 Hz, 2H, CH_2), 3.93 (s, 3H, OCH_3), 1.39 (t, J = 7.1 Hz, 3H, CH_3); ^{13}C NMR (101 MHz, $CDCl_3$): δ (ppm) = 191.1 (CHO), 152.6 (C=O), 152.2 (C-2), 145.1 (C- 1), 135.4 (C-4), 124.8 (C-5), 123.1 (C-6), 111.1 (C-3), 65.5 (CH_2), 56.3 (OCH_3), 14.3 (CH_3); IR (ATR) $\tilde{\nu}_{max}/cm^{-1}$ = 1753, 1699, 1599, 1508, 1393, 1264, 1215, 1154, 1131, 1031, 994, 961, 899, 863, 825, 804, 7714, 725; HRMS (EI): calcd. for $C_{11}H_{12}O_5$ 225.0763 $[M+H]^+$; found 225.0760; purity (HPLC): >99%.

Ethyl (4-formylphenyl) carbonate

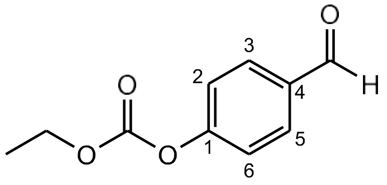

Synthesis and characterization of ethyl (4-formylphenyl) carbonate was reported previously in [1].

Ethyl (4-(2-methoxyvinyl)phenyl) carbonate (B1, *E*/*Z* mixture)

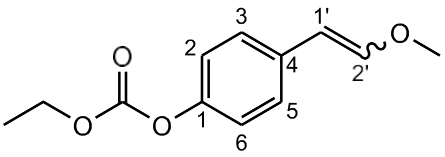

Synthesis and characterization of compound **B1** was reported previously in [1].

Ethyl (2-methoxy-5-(2-methoxyvinyl)phenyl) carbonate (B2, *E*/*Z* mixture)

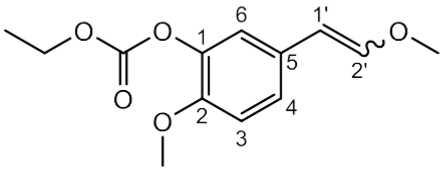

Following general procedure B, (methoxymethyl)triphenylphosphonium chloride (1.8 g, 5.27 mmol; 1.2 equiv) in 20 mL dry THF, 1.12 mL LDA (2 M in THF, 4.39 mmol, 1 equiv) and ethyl(5-formyl-2-methoxyphenyl) carbonate (985 mg; 4.39 mmol, 1.0 equiv) in 20 mL dry THF were used. The reaction was completed after 2 h and the crude product was purified by FCC using hexane/EtOAc (EtOAc 0% up to 20%) to yield the desired *cis/trans*-enol ether (526 mg, 2.09 mmol, 47%) as a light yellow oil. ^{1}H NMR (400 MHz, $CDCl_3$): δ (ppm) = 7.48 (d, J = 2.1 Hz, 1H, *cis* 6-H), 7.32 (dd, J = 8.5, 2.1 Hz, 1H, *cis* 4-H), 7.10 – 6.99 (m, 2H, *trans* 4-H, *trans* 6-H), 6.94 – 6.85 (m, 3H, *trans* 2‘-H; *cis* 3-H, *trans* 3-H), 6.07 (d, J = 6.9 Hz, 1H, *cis* 2‘-H), 5.73 (d, J = 12.9 Hz, 1H, *trans* 1‘-H), 5.14 (d, J = 6.9 Hz, 1H, *cis* 1‘-H), 4.31 (qd, J = 7.1, 1.2 Hz, 4H, *cis*/*trans* $C\underline{H}_2CH_3$), 3.83 (d, J = 2.1 Hz, 6H, *cis*/*trans* 2-OCH_3), 3.75 (s, 3H, *cis* 2‘-OCH_3), 3.66 (s, 3H, *trans* 2’- OCH_3), 1.38 (td, J = 7.1, 1.3 Hz, 6H, *cis*/*trans* $CH_2C\underline{H}_3$); ^{13}C NMR (101 MHz, $CDCl_3$): δ (ppm) = 153.5 (*cis*/*trans* OCOO), 149.4 (*trans* C-2), 149.2 (*cis* C-2), 148.5 (*trans* C- 2’), 147.3 (*cis* C-2’), 140.4 (*cis* C-1), 139.9 (*trans* C-1), 129.7 (*trans* C-5), 129.4 (*cis* C- 5), 126.9 (*cis* C-4), 123.8 (*trans* C-4), 122.3 (*cis* C-6), 119.1 (*trans* C-6), 112.9 (*cis* C- 3), 112.3 (*trans* C-3), 104.7 (*trans* C-1‘), 104.1 (*cis* C-1’), 65.0 (*cis*/*trans* $\underline{C}H_2CH_3$), 60.7 (*cis* 2’-OCH_3), 56.7 (*trans* 2’-OCH_3), 56.2 (*cis* 2-OCH_3), 56.1 (*trans* 2-OCH_3), 14.3 (*cis*/*trans* $CH_2\underline{C}H_3$); *cis/trans*-ratio according to ^{1}H-NMR is 0.81; IR (ATR): $\tilde{\nu}_{max}$ [cm^{-1}] = 3448, 2937, 1759, 1642, 1511, 1454, 1368, 1246, 1210, 1133, 1091, 1025, 930, 766; HR-MS (ESI): calcd. for $C_{13}H_{16}O_5$: 253.1076 $[M+H]^+$, found 253.1072; purity (HPLC): >90%.

Ethyl (2-methoxy-4-(2-methoxyvinyl)phenyl) carbonate (B3, *E*/*Z* mixture)

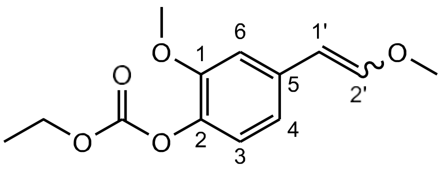

Following general procedure B, (methoxymethyl)triphenylphosphonium chloride (917 mg; 2.68 mmol; 1.2 equiv) in 10 mL dry THF, LDA (2 M in THF, 1.12 mL 2.23 mmol; 1 equiv) and a solution of ethyl (4-formyl-2-methoxyphenyl) carbonate (500 mg; 2.23 mmol, 1.0 equiv) in 5 mL dry THF were used. The reaction was completed after 2 h and the crude product was purified by FCC using hexanes/CH_2Cl_2 (CH_2Cl_2 2% up to 50%) to yield the desired *cis/trans*-enol ether (234 mg, 0.928 mmol, 41%) as a yellow oil. ^{1}H NMR (400 MHz, $CDCl_3$): δ (ppm) = 7.29 (d, *J* = 1.9 Hz, 1H, *cis* 6-H), 7.08 (dd, *J* = 8.3, 1.9 Hz, 1H, *cis* 4-H), 7.04 – 6.98 (m, 3H, *trans* 3-H, *cis* 3-H, *trans* 2'-H), 6.82-6.77 (m, 2H, *trans* 4-H, *trans* 6-H), 6.13 (d, *J* = 7.0 Hz, 1H, *cis* 2'-H), 5.78 (d, *J* = 13.0 Hz, 1H, *trans* 1'-H), 5.19 (d, *J* = 7.0 Hz, 1H, *cis* 1'-H), 4.30 (qd, *J* = 7.2, 1.5 Hz, 4H, *cis*/*trans* C<u>H</u>$_2$CH$_3$), 3.85 (d, *J* = 0.8 Hz, 6H, *cis/trans* 1-OCH$_3$,), 3.78 (s, 3H, *cis* 2'-OCH$_3$), 3.68 (s, 3H, *trans* 2'-OCH$_3$), 1.38 (td, *J* = 7.1, 1.8 Hz, 6H, *cis*/*trans* CH$_2$C<u>H</u>$_3$); ^{13}C NMR (101 MHz, $CDCl_3$): δ (ppm) = 153.6 (*cis/trans* OCOO), 151.3 (*trans* C-1), 150.8 (*cis* C-1), 149.3 (*trans* C-2'), 148.2 (*cis* C-2'), 138.3 (*cis C-2*), 138.1 (*trans* C-2), 135.8 (*trans* C- 5), 135.2 (*cis* C-5), 122.5 (*trans* C-3), 122.0 (*cis* C-3), 120.8 (*cis* C-4), 117.5 (*trans* C- 4), 112.6 (*cis* C-6), 109.4 (*trans* C-6), 105.3 (*cis* C-1'), 104.7 (*trans* C-1'), 65.0 (*cis*/*trans* <u>C</u>H$_2$CH$_3$), 60.9 (*cis* 2'-OCH$_3$), 56.7 (*trans* 2'-OCH$_3$), 56.0 (*cis/trans* 1-OCH$_3$), 14.3 (*cis*/*trans* CH$_2$<u>C</u>H$_3$); c*is/trans*-ratio according to ^{1}H-NMR is 0.87; IR (ATR): $\tilde{\nu}_{max}$ [cm^{-1}] = 2934, 1758, 1641, 1601, 1511, 1368, 1247, 1202, 1154, 1091, 1031, 776; HR-MS (ESI): calcd. for $C_{13}H_{16}O_5$: 253.1076 [M+H]$^+$, found 253.1072; purity (HPLC): >95%.

2-(2,3,4-Trimethoxyphenyl)ethan-1-amine

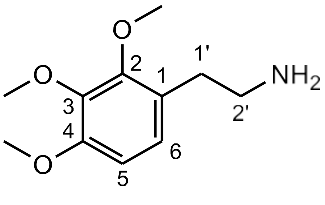

Synthesis and the ^{1}H NMR spectrum of 2-(2,3,4-trimethoxyphenyl)ethan-1-amine was reported previously in [4]. The synthesis was adapted and the analytical data was complemented.

1,2,3-Trimethoxy-4-(2-nitrovinyl)benzene (2.00 g, 8.36 mmol, 1 equiv), granular zinc 20–30 mesh (6.56 g, 100 mmol, 12 equiv) and aqueous HCl (37%, 18.8 mL, 201 mmol, 24 equiv) were added alternating in portions to 20 mL EtOH at 0 °C over 30 min. The reaction mixture was stirred at 0 °C for 4 h, before excessive zinc was filtered off. The *pH* was adjusted to pH 8 using methanolic sodium hydroxide. The precipitate (zinc hydroxide) was filtered off and washed with methanol. The organic solvents were removed *in vacuo* from the filtrate and the remaining aqueous solution was extracted with dichloromethane (3 × 100 mL). The combined organic layers were dried over Na_2SO_4 and filtered. Concentration *in vacuo* gave the crude product which was purified by FCC using CH_2Cl_2/C_2H_5OH (C_2H_5OH up to 10%) yielding the desired amine as an yellow oil (168 mg, 0.795 mmol, 9.5%). ^1H NMR (400 MHz, $CDCl_3$) δ (ppm) = 6.84 (d, J = 8.5 Hz, 1H, 6-H), 6.60 (d, J = 8.4 Hz, 1H, 5-H), 3.91 – 3.75 (m, 9H, 2-OCH_3, 3-OCH_3, 4-OCH_3), 2.92 (t, J = 7.0 Hz, 2H, 2'-H), 2.72 (t, J = 7.0 Hz, 2H, 1'-H), 2.30 (s, 2H, -NH_2); ^{13}C NMR (101 MHz, $CDCl_3$): δ (ppm) = 152.5 (C-2), 152.2 (C-4), 142.5 (C-3), 125.4 (C-1), 124.5 (C-6), 107.3 (C-5), 61.1 (3-OCH_3), 60.9 (2-OCH_3), 56.1 (4-OCH_3), 42.9 (C-2'), 33.7 (C-1'); IR (ATR): $\tilde{\nu}_{max}$ [cm^{-1}] = 2935, 1599, 1493, 1466, 14,16, 1265, 1096, 1015, 799, 731, 700; HR-MS (ESI): calcd. for $C_{11}H_{17}NO_3$: 212.1287 $[M+H]^+$, found 212.1283; purity (HPLC): >99%.

Ethyl (3,4-dimethoxyphenethyl) carbamate (A1)

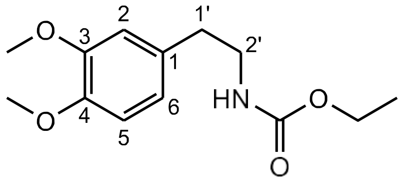

Synthesis and characterization of compound **A1** was reported previously in [5].

Ethyl (2-(benzo[*d*][1,3]dioxol-5-yl)ethyl) carbamate (A2)

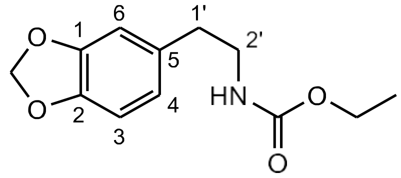

Synthesis of compound **A2** was reported previously in [6]. A different synthetic approach and full analytical data is provided subsequently. Following general

procedure A, 3,4-(methylenedioxyphenyl)ethylamine (2.00 g; 11.5 mmol; 1.0 equiv) in 25 mL dry CH_2Cl_2, NEt_3 (4.8 mL; 34.5 mmol; 3 equiv) and ethyl chloroformate (2.2 mL; 23 mmol; 2 equiv) were used. The mixture was heated to reflux for 4 h and purification by flash column chromatography (FCC) using hexanes/ EtOAc (EtOAc 3% up to 35%) yielded the desired carbamate (2.39 g, 10.1 mmol, 87%) as a colorless solid [6]. mp: 40.6 °C; ^{1}H NMR (400 MHz, $C_2D_2Cl_4$): δ (ppm) = 6.68 (d, J = 7.9 Hz, 1H, 3-H), 6.61 (d, J = 1.7 Hz, 1H, 6-H), 6.56 (dd, J = 7.9, 1.7 Hz, 1H, 4-H), 5.86 (s, 2H, O-CH_2-O), 4.62 (s, 1H, NH), 3.99 (q, J = 7.1 Hz, 2H, $OC\underline{H}_2$-CH_3), 3.27 (t, J = 6.6 Hz, 2H, 2'-H), 2.63 (t, J = 7.1 Hz, 2H, 1'-H), 1.14 (t, J = 7.1 Hz, 3H, OCH_2-$C\underline{H}_3$); ^{13}C NMR (101 MHz, $C_2D_2Cl_4$): δ (ppm) = 156.7 (NCO), 147.9 (C-1), 146.3 (C-2), 132.9 (C-5), 122.0 (C-4), 109.4 (C-6), 108.7 (C-3), 101.3 (O-CH_2-O), 61.1 ($OC\underline{H}_2$-CH_3), 42.5 (C-2'), 36.1 (C-1'), 15.0 (OCH_2-$\underline{C}H_3$); IR (ATR): $\tilde{\nu}_{max}$ [cm^{-1}] = 3320, 2872, 1682, 1540, 1501, 1478, 1438, 1307, 1240, 1194, 1150, 1032, 924, 819, 781; HR-MS (ESI): calcd. for $C_{12}H_{15}NO_4$: 238.1079 [M+H]$^+$, found 238.1075; purity (HPLC): >99%.

Ethyl (4-hydroxy-3-methoxyphenethyl)carbamate (A3)

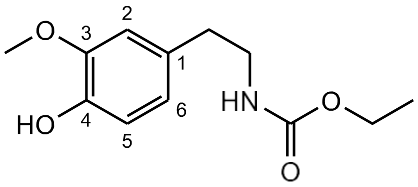

Synthesis and characterization of compound **A3** was reported previously in [1].

Ethyl (2,3,4-trimethoxyphenethyl) carbamate (A4)

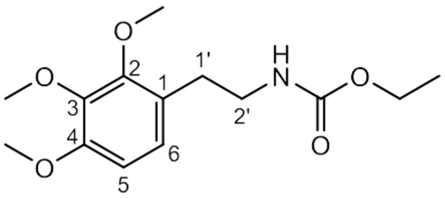

Synthesis of compound **A4** was reported previously in [7]. A different synthetic approach and full analytical data is provided subsequently. Following general procedure A, 2-(2,3,4-trimethoxyphenyl)ethan-1-amine (450 mg; 1.92 mmol; 1.0 equiv) in 5 mL dry CH_2Cl_2, NEt_3 (0.800 mL; 5.75 mmol; 3 eq.) and ethyl chloroformate (367 µL; 23.0 mmol; 2 equiv) were used. The mixture was heated to reflux for 4 h and purification by flash column chromatography (FCC) using hexane/ EtOAc (EtOAc up

to 30%) yielded the desired carbamate (286 mg, 1.0 mmol, 56%) as a yellow oil. ^{1}H NMR (400 MHz, $C_2D_2Cl_4$): δ (ppm)= 6.82 (d, J = 8.5 Hz, 1H, 6-H), 6.61 (d, J = 8.5 Hz, 1H, 5-H), 4.81 (s, 1H, NH), 4.09 (q, J = 7.1 Hz, 2H, $OC\underline{H}_2CH_3$), 3.89 (s, 3H, 2-OCH_3), 3.86 (s, 3H, 3-OCH_3), 3.84 (s, 3H, 4-OCH_3), 3.36 (m, 2H, 2'-H), 2.75 (t, J = 6.9 Hz, 2H, 1'-H), 1.22 (t, J = 7.1 Hz, 3H, $OCH_2C\underline{H}_3$); ^{13}C NMR (101 MHz, $C_2D_2Cl_4$): δ (ppm) = 156.8 (C=O), 152.7 (C-4), 152.2 (C-2), 142.4 (C-3), 124.9 (C-1), 124.6 (C-6), 107.5 (C-5), 61.1 (2-OCH_3), 60.9 (3-OCH_3), 60.8 ($O\underline{C}H_2CH_3$), 56.1 (4-OCH_3), 42.0 (C-2'), 30.4 (C-1'), 14.8 ($OCH_2\underline{C}H_3$); IR (ATR): $\tilde{\nu}_{max}$ [cm^{-1}] = 3346, 2934, 1693, 1524, 1493, 1466, 1416, 1243, 1097, 1065, 1035, 1015, 902, 799; HR-MS (ESI): calcd. for $C_{14}H_{21}NO_5$: 284.1498 $[M+H]^+$, found 284.1495; purity (HPLC): >99%.

(±)-Ethyl 1-(4-((ethoxycarbonyl)oxy)benzyl)-6,7-dimethoxy-3,4-dihydroisoquinoline-2(1*H*)-carboxylate (5a)

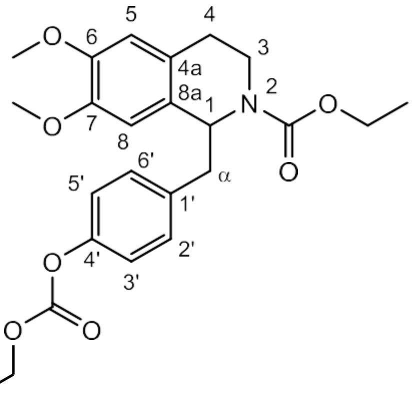

Synthesis and characterization of compound **5a** was reported previously in [1].

(±)-Ethyl 1-(3-((ethoxycarbonyl)oxy)-4-methoxybenzyl)-6,7-dimethoxy-3,4-dihydroisoquinoline-2(1*H*)-carboxylate (5b)

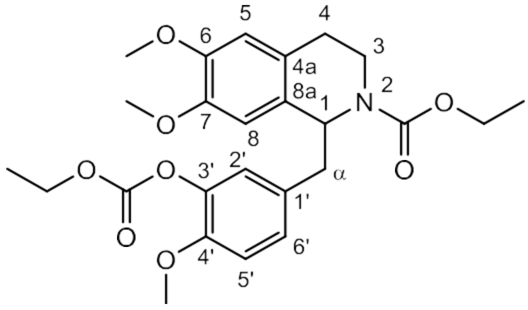

Following general procedure C, ethyl (3,4-dimethoxyphenethyl) carbamate (**A1**, 288 mg, 1.14 mmol, 1 equiv), ethyl (2-methoxy-5-(2-methoxyvinyl)phenyl) carbonate (**B2**, 287 mg, 1.14 mmol, 1 equiv) and TFA (0.85 mL, 11.4 mmol, 10 equiv) were used. The reaction was completed after 12 h and the crude product was purified by FCC using hexanes/ EtOAc (EtOAc 5% up to 35%) yielding the desired tetrahydroisoquinoline (**5b**, 178 mg, 0.376 mmol, 33%) as a yellow solid. mp: 58 °C; ^{1}H NMR (400 MHz, $C_2D_2Cl_4$, 100 °C) *δ* (ppm) = 6.88 – 6.77 (m, 3H, 2'-H, 5'-H, 6'-H), 6.56 (s, 1H, 5-H), 6.27 (s, 1H, 8-H), 5.14 (t, *J* = 6.6 Hz, 1H, 1-H), 4.25 (q, *J* = 7.1 Hz, 2H, $OCOOC\underline{H}_2CH_3$), 4.05 (q, *J* = 7.0 Hz, 2H, $NCOOC\underline{H}_2CH_3$), 3.98 – 3.85 (m, 1H, 3-H), 3.78 (s, 3H, 6-OCH_3), 3.77 (s, 3H, 4'-OCH_3), 3.63 (s, 3H, 7-OCH_3), 3.27 (ddd, *J* = 13.4, 9.3, 4.7 Hz, 1H, 3-H), 3.03 (dd, *J* = 13.6, 6.4 Hz, 1H, α-H), 2.90 (dd, *J* = 13.6, 7.0 Hz, 1H, α-H), 2.75 (ddd, *J* = 15.5, 9.4, 5.8 Hz, 1H, 4-H), 2.54 (dt, *J* = 15.9, 4.8 Hz, 1H, 4-H), 1.33 (t, *J* = 7.1 Hz, 3H, $OCOOCH_2C\underline{H}_3$), 1.22 – 1.15 (t, *J* = 7.5 Hz, 3H, $NCOOCH_2C\underline{H}_3$); ^{13}C NMR (101 MHz, $C_2D_2Cl_4$, 100 °C) *δ* (ppm) = 155.7 (N-CO), 153.2 (O-CO), 150.3 (C-4'), 148.8 (C-6), 148.2 (C-7), 140.8 (C-3'), 131.7 (C-1'), 129.1 (C-8a), 128.2 (C-5'), 127.1 (C-4a), 123.8 (C-6'), 113.5 (C-2'), 113.3 (C-5), 112.3 (C-8), 65.0 ($OCOO\underline{C}H_2CH_3$), 61.5 ($NCOO\underline{C}H_2CH_3$), 56.7 (6-OCH_3), 56.7 (7-OCH_3), 56.6 (4'-OCH_3), 56.4 (C-1), 42.3 (C-α), 39.1 (C-3), 28.2 (C-4), 14.8 ($NCOOCH_2\underline{C}H_3$), 14.4 ($OCOOCH_2\underline{C}H_3$); IR (ATR): $\tilde{\nu}_{max}$ [cm^{-1}] = 2935, 1760, 1689, 1513, 1428, 1368, 1246, 1214, 1099, 1026, 766; HR-MS (ESI): calcd. for $C_{25}H_{31}NO_8$: 474.2128 $[M+H]^+$, found 474.2123; purity (HPLC): >99%.

(±)-Ethyl 1-(4-((ethoxycarbonyl)oxy)-3-methoxybenzyl)-6,7-dimethoxy-3,4-dihydroisoquinoline-2(1*H*)-carboxylate (5c)

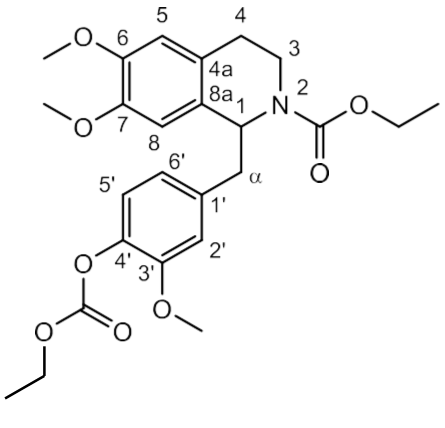

Following general procedure C, ethyl (3,4-dimethoxyphenethyl) carbamate (**A1**, 279 mg, 1.1 mmol, 1 equiv), ethyl (2-methoxy-4-(2-methoxyvinyl)phenyl) carbonate (**B3**, 278

mg, 1.1 mmol, 1 equiv) and TFA (0.83 mL, 11 mmol, 10 equiv) were used. The reaction was completed after 12 h and the crude product was purified by FCC using hexanes/ EtOAc (EtOAc 5% up to 35 %) yielding the desired tetrahydroisoquinoline (**5c**, 240 mg; 0.507 mmol, 46%) as a yellow oil. ^{1}H NMR (400 MHz, $C_2D_2Cl_4$, 100 °C) *δ* (ppm) = 6.96 (d, *J* = 8.4 Hz, 1H, 5'-H), 6.63 (dd, *J* = 4.3, 2.4 Hz, 2H, 2'-H, 6'-H), 6.57 (s, 1H, 5-H), 6.26 (s, 1H, 8-H), 5.17 (t, *J* = 6.6 Hz, 1H, 1-H), 4.24 (q, *J* = 7.1 Hz, 2H, $OCOOC\underline{H}_2CH_3$), 4.15 – 4.01 (m, 2H, $NCOOC\underline{H}_2CH_3$), 4.00-3,86 (m, 1H, 3-H), 3.78 (s, 3H, 6-OCH_3), 3.72 (s, 3H, 3'-OCH_3), 3.63 (s, 3H, 7-OCH_3), 3.28 (ddd, *J* = 13.0, 9.3, 4.8 Hz, 1H, 3-H), 3.08 (dd, *J* = 13.4, 6.3 Hz, 1H, α-H), 2.93 (dd, *J* = 13.5, 7.1 Hz, 1H, α-H), 2.76 (ddd, *J* = 15.4, 9.3, 5.8 Hz, 1H, 4-H), 2.55 (dt, *J* = 15.9, 4.7 Hz, 1H, 4-H), 1.33 (t, *J* = 7.1 Hz, 3H, $OCOOCH_2C\underline{H}_3$), 1.19 (t, *J* = 7.1 Hz, 3H, $NCOOCH_2C\underline{H}_3$); ^{13}C NMR (101 MHz, $C_2D_2Cl_4$, 100 °C) *δ* (ppm) = 155.8 (N-CO), 153.3 (O-CO), 151.3 (C-3'), 148.9 (C-6), 148.2 (C-7), 139.8 (C-4'), 137.7 (C-1'), 129.0 (C-8a), 127.1 (C-4a), 122.4 (C-5'), 122.2 (C-6'), 115.2 (C-2'), 113.3 (C-5), 112.5 (C-8), 65.0 ($OCOO\underline{C}H_2CH_3$), 61.5 ($NCOO\underline{C}H_2CH_3$), 56,7 (3'-OCH_3), 56.7 (6-OCH_3), 56.6 (7-OCH_3), 56.4 (C-1), 43.1 (C-α), 40.2 (C-3), 28.3 (C-4), 14.9 ($OCOOCH_2\underline{C}H_3$), 14.4 ($NCOOCH_2\underline{C}H_3$); IR (ATR): $\tilde{v}_{max}$ [cm^{-1}] = 2935, 1760, 1687, 1606, 1511, 1420, 1241, 1202, 1099, 1031, 859, 762; HR- MS (ESI): calcd. for $C_{25}H_{31}NO_8$: 474.2128 $[M+H]^+$, found 474.2123; purity (HPLC): >99%.

(±)-Ethyl 1-(3-((ethoxycarbonyl)oxy)-4-methoxybenzyl)-7-hydroxy-6-methoxy-3,4-dihydroisoquinoline-2(1*H*)-carboxylate (5d)

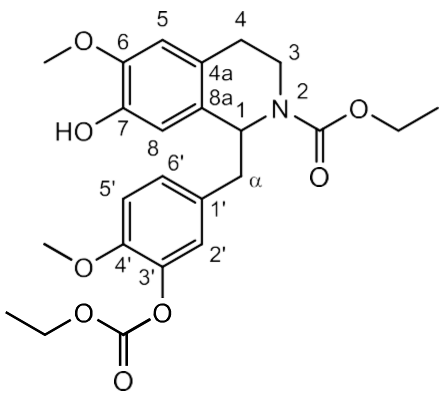

Following general procedure C, ethyl (4-hydroxy-3-methoxyphenethyl) carbamate (**A3**, 210 mg, 0.878 mmol, 1 equiv), ethyl (2-methoxy-5-(2-methoxyvinyl)phenyl) carbonate (**B2**, 221 mg, 0.878 mmol, 1 equiv) and TFA (0.66 mL, 8.78 mmol, 10 equiv) were used.

The reaction was completed after 12 h and the crude product was purified by FCC using hexanes/ EtOAc (EtOAc up to 40%) yielding the desired tetrahydroisoquinoline (**5d,** 141 mg, 0.307 mmol, 35%) as a yellow oil. ^{1}H NMR (400 MHz, $C_2D_2Cl_4$, 100 °C): *δ* (ppm) = 6.87 (dd, *J* = 8.3, 2.1 Hz, 1H, 6'-H), 6.82 (d, *J* = 8.3 Hz, 1H, 5'-H), 6.79 (d, *J* = 2.0 Hz, 1H, 2'-H), 6.54 (s, 1H, 5-H), 6.50 (s, 1H, 8-H), 5.33 (s, 1H, -O<u>H</u>), 5.12 (t, *J* = 6.5 Hz, 1H, 1-H), 4.25 (q, *J* = 7.1, 2H, OCOOC<u>H</u>$_2$CH$_3$), 4.06 – 3.87 (m, 3H, NCOOC<u>H</u>$_2$CH$_3$, 3-H), 3.82 (s, 3H, 6-OCH$_3$), 3.78 (s, 3H, 4'-OCH$_3$), 3.20 (ddd, *J* = 13.5, 9.6, 4.5 Hz, 1H, 3-H), 2.96 (d, *J* = 6.5 Hz, 2H, α-H), 2.73 (ddd, *J* = 15.4, 9.5, 5.8 Hz, 1H, 4-H), 2.48 (dt, *J* = 15.7, 4.6 Hz, 1H, 4-H), 1.33 (t, *J* = 7.1, 3H, OCOOCH$_2$C<u>H</u>$_3$), 1.13 (t, *J* = 7.1 Hz, 3H, NCOOCH$_2$C<u>H</u>$_3$); ^{13}C NMR (101 MHz, $C_2D_2Cl_4$, 100 °C): *δ* (ppm) = 155.7 (N-CO), 153.2 (O-CO), 150.3 (C-4'), 146.1 (C-6), 144.4 (C-7), 140.7 (C-3'), 131.7 (C-1'), 129.9 (C-8a), 128.1 (C-6'), 126.4 (C-4a), 123.8 (C-2'), 113.6 (C-8), 113.5 (C-5'), 111.7 (C-5), 65.0 (OCOO<u>C</u>H$_2$CH$_3$), 61.4 (NCOO<u>C</u>H$_2$CH$_3$), 56.7 (4'-OCH$_3$), 56.5 (6-OCH$_3$), 56.1 (C-1), 42.2 (C-α), 39,0 (C-3), 28.3 (C-4), 14.8 (NCOOCH$_2$<u>C</u>H$_3$), 14.4 (OCOOCH$_2$<u>C</u>H$_3$); IR (ATR): $\tilde{\nu}_{max}$ [cm^{-1}] = 3354, 2934, 1760, 1682, 1511, 1428, 1369, 1247, 1214, 1098, 1027, 890, 732; HR-MS (ESI): calcd. for $C_{24}H_{29}NO_8$: 460.1971 [M+H]$^+$, found 460.1967; purity (HPLC): >99%.

(±)-Ethyl 1-(4-((ethoxycarbonyl)oxy)-3-methoxybenzyl)-7-hydroxy-6-methoxy-3,4-dihydro-isoquinoline-2(1*H*)-carboxylate (5e)

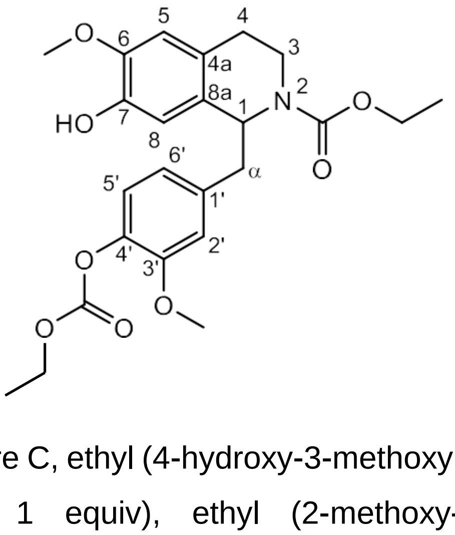

Following general procedure C, ethyl (4-hydroxy-3-methoxyphenethyl) carbamate (**A3**, 212 mg, 0.888 mmol, 1 equiv), ethyl (2-methoxy-4-(2-methoxyvinyl)phenyl) carbonate (**B3**, 224 mg, 0.888 mmol, 1 equiv) and TFA (0.666 mL, 8.88 mmol, 10 equiv) were used. The reaction was completed after 12 h and the crude product was purified by FCC using hexanes/ EtOAc (EtOAc 5% up to 50%) yielding the desired

tetrahydroisoquinoline (**5e**, 210 mg, 0.457 mmol, 52%) as a colourless solid. mp: 67 °C; ^{1}H NMR (400 MHz, $C_2D_2Cl_4$, 100 °C): *δ* (ppm) = 6.95 (d, *J* = 8.5 Hz, 1H, 5'-H), 6.67 – 6.60 (m, 2H, 2'-H, 6'-H), 6.54 (s, 1H, 5-H), 6.51 (s, 1H, 8-H), 5.16 (t, *J* = 6.6 Hz, 1H, 1-H), 4.24 (q, *J* = 7.1 Hz, 2H, OCOOC$\underline{H}_2$CH$_3$), 3.96 (m, 3H, NCOOC$\underline{H}_2$CH$_3$, 3-H), 3.82 (s, 3H, 3'-OCH$_3$), 3.72 (s, 3H, 6-OCH$_3$), 3.20 (ddd, *J* = 13.1, 9.8, 4.6 Hz, 1H, 3-H), 3.00 (d, *J* = 6.5 Hz, 2H, α-H), 2.74 (ddd, *J* = 15.8, 9.7, 5.8 Hz, 1H, 4-H), 2.48 (d, *J* = 15.9 Hz, 1H, 4-H), 1.32 (t, *J* = 7.1 Hz, 3H, OCOOCH$_2$C$\underline{H}_3$), 1.12 (t, *J* = 7.0 Hz, 3H, NCOOCH$_2$C$\underline{H}_3$); ^{13}C NMR (101 MHz, $C_2D_2Cl_4$, 100 °C) *δ* (ppm) = 155.7 (N-CO), 153.3 (O-CO), 151.2 (C-3'), 146.1 (C-6), 144.4 (C-7), 139.7 (C-4'), 137.7 (C-1'), 129.8 (C-8a), 126.4 (C-4a), 122.2 (C-6'), 122.1 (C-5'), 115.1 (C-2'), 113.6 (C-8), 111.6 (C-5), 65.0 (OCOO$\underline{C}$H$_2$CH$_3$), 61.5 (NCOO$\underline{C}$H$_2$CH$_3$), 56.5 (3'-OCH$_3$), 56.5 (6-OCH$_3$), 56.1 (C-1); 42.5 (C-α), 38.6 (C-3), 28.3 (C-4), 14.8 (NCOOCH$_2\underline{C}$H$_3$), 14.4 (OCOOCH$_2\underline{C}$H$_3$); IR (ATR): $\tilde{\nu}_{max}$ [cm^{-1}] = 2935, 1760, 1682, 1510, 1420, 1247, 1202, 1155, 1098, 1031, 869, 763; HR-MS (ESI): calcd. for $C_{24}H_{29}NO_8$: 460.1971 [M+H]$^+$, found 460.1967; purity (HPLC): >95%.

(±)-Ethyl (*S*)-5-(4-((ethoxycarbonyl)oxy)benzyl)-7,8-dihydro-[1,3]dioxolo[4,5-*g*]isoquinoline-6(5*H*)-carboxylate (5f)

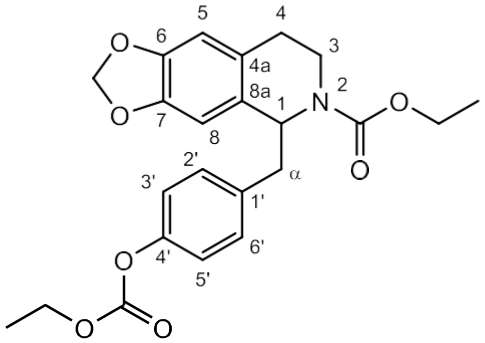

Following general procedure C, ethyl (2-(benzo[*d*][1,3]dioxol-5-yl)ethyl) carbamate (**A2**, 452 mg, 1.9 mmol, 1 equiv), ethyl (4-(2-methoxyvinyl)phenyl) carbonate (**B1**, 423 mg, 1.9 mmol, 1 equiv) and TFA (1.43 mL, 10 mmol, 10 equiv) were used. The reaction was completed after 16 h and the crude product was purified by FCC using hexanes/ EtOAc (EtOAc up to 30%) yielding the desired tetrahydroisoquinoline (**5f**, 429 mg, 1.0 mmol, 53%) as a colorless solid. mp: 115 °C; ^{1}H NMR (400 MHz, $C_2D_2Cl_4$, 100 °C): *δ* (ppm) = 7.04 (m, 4H, 2'-H, 3'-H, 5'-H, 6'-H), 6.53 (s, 1H, 5-H), 6.39 (s, 1H, 8-H), 5.85 (dd, *J* = 11.1, 1.4 Hz, 2H, O-CH$_2$-O), 5.14 (m, 1H, 1-H), 4.25 (q, *J* = 7.1 Hz, 2H, OCOOC$\underline{H}_2$CH$_3$), 3.95 (m, 3H, NCOOC$\underline{H}_2$CH$_3$, 3-H), 3.24 (ddd, *J* = 13.5, 9.3, 4.6 Hz,

1H, 3-H), 2.99 (dd, J = 6.8, 3.4 Hz, 2H, α-H), 2.79-2.64 (m, 1H, 4-H), 2.56-2.44 (m, 1H, 4-H), 1.33 (t, J = 7.1 Hz, 3H, OCOOCH$_2$C<u>H</u>$_3$), 1.10 (m, 3H, NCOOCH$_2$C<u>H</u>$_3$); ^{13}C NMR (101 MHz, $C_2D_2Cl_4$, 100 °C): δ (ppm) = 155.6 (N-CO), 153.6 (O-CO), 150.3 (C-4'), 146.8 (C-7), 146.3 (C-6), 136.2 (C-4a), 130.7 (C-2', C-6'), 130.0 (C-8a), 128.1 (C-1'), 120.9 (C-3', C-5') 108.8 (C-5), 107.5 (C-8), 101.1 (O-CH$_2$-O), 65.0 (OCOO<u>C</u>H$_2$CH$_3$), 61.5 (NCOO<u>C</u>H$_2$CH$_3$), 56.7 (C-1), 42.6 (C-α), 38.7 (C-3), 28.7 (C-4), 14.8 (NCOOCH$_2$<u>C</u>H$_3$), 14.5 (OCOOCH$_2$<u>C</u>H$_3$); IR (ATR): $\tilde{\nu}_{max}$ [cm^{-1}] = 2984, 1743, 1687, 1487, 1428, 1369, 1263, 1215, 1037, 1003, 933, 862, 783, 764; HR-MS (ESI): calcd. for $C_{23}H_{25}NO_7$: 428.1709 [M+H]$^+$, found 428.1704; purity (HPLC): >99%.

(±)-Ethyl 5-(3-((ethoxycarbonyl)oxy)-4-methoxybenzyl)-7,8-dihydro-[1,3]dioxolo[4,5-*g*]isoquinoline-6(5*H*)-carboxylate (5g)

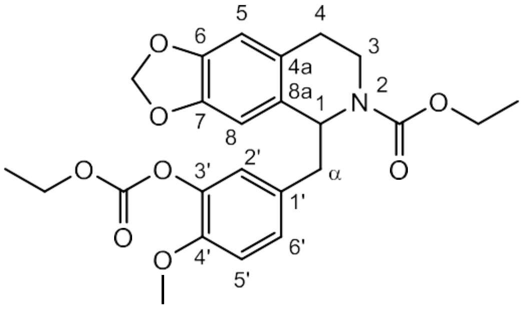

Following general procedure C, ethyl (2-(benzo[*d*][1,3]dioxol-5-yl)ethyl) carbamate (**A2**, 205 mg, 0.865 mmol, 1 equiv), ethyl (2-methoxy-5-(2-methoxyvinyl)phenyl) carbonate (**B2**, 220 mg, 0.865 mmol, 1 equiv) and TFA (0.85 mL, 11.4 mmol, 10 equiv) were used. The reaction was completed after 16 h and the crude product was purified by FCC using hexanes/ EtOAc (EtOAc 2% up to 35%) yielding the desired tetrahydroisoquinoline (**5g**, 124 mg, 0.271 mmol, 31%) as a yellow solid. mp: 58 °C; ^{1}H NMR (400 MHz, $C_2D_2Cl_4$, 100 °C): δ (ppm) = 6.86 (dd, J = 8.4, 2.0 Hz, 1H, 6'-H), 6.82 (d, J = 8.3 Hz, 1H, 5'-H), 6.79 (d, J = 2.0 Hz, 1H, 2'-H), 6.53 (s, 1H, 5-H), 6.36 (s, 1H, 8-H), 5.84 (dd, J = 9.2, 1.4 Hz, 2H, O-CH$_2$-O), 5.12 (t, J = 6.6 Hz, 1H, 1-H), 4.25 (q, J = 7.1 Hz, 2H, OCOOC<u>H</u>$_2$CH$_3$), 4.02 (m, 2H, NCOOC<u>H</u>$_2$CH$_3$), 3.92 – 3.81 (m, 1H, 3-H), 3.78 (s, 3H, OCH$_3$), 3.25 (ddd, J = 13.4, 9.0, 4.8 Hz, 1H, 3-H), 3.08 – 2.86 (m, 2H, α-H), 2.82 – 2.63 (m, 1H, 4-H), 2.49 (dt, J = 16.0, 5.0 Hz, 1H, 4-H), 1.33 (t, J = 7.1 Hz, 3H, OCOOCH$_2$C<u>H</u>$_3$), 1.15 (t, J = 7.1 Hz, 3H, NCOOCH$_2$C<u>H</u>$_3$); ^{13}C NMR (101 MHz, $C_2D_2Cl_4$, 100 °C): δ (ppm) = 155.7 (N-CO), 153.2 (O-CO), 150.3 (C-4'), 146.8 (C-6), 146.3 (C-7), 140.7 (C-3'), 131.5 (C-1'), 130.1 (C-8a), 128.1 (C-6'), 128.0 (C-4a), 123.8

(C-2’), 113.5 (C-5’), 108.7 (C-5), 107.6 (C-8), 101.0 (O-CH2-O), 65.0 (OCOOC̲H2CH3), 61.5 (NCOOC̲H2CH3), 56.7 (C-1), 56.7 (OCH3), 42.3 (C-α), 39.0 (C-3), 28.6 (C-4), 14.8 (NCOOCH2C̲H3), 14.4 (OCOOCH2C̲H3); IR (ATR): $\tilde{\nu}_{max}$ [cm^{-1}] = 2931, 1760, 1686, 1483, 1427, 1240, 1211, 1133, 1107, 1033, 923, 763; HR-MS (ESI): calcd. for $C_{24}H_{27}NO_8$: 458.1815 [M+H]$^+$, found 458.1810; purity (HPLC): 99%.

(±)-Ethyl 1-(4-((ethoxycarbonyl)oxy)benzyl)-5,6,7-trimethoxy-3,4-dihydroisoquinoline-2(1*H*)-carboxylate (5h)

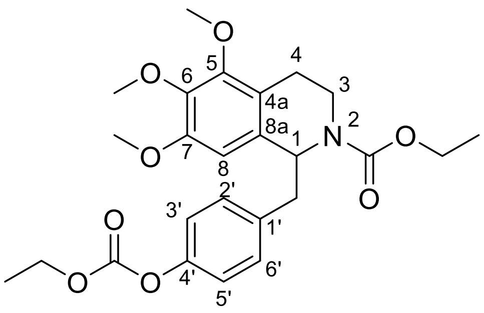

Following general procedure C, ethyl (2,3,4-trimethoxyphenethyl) carbamate (**A4**, 60 mg, 0.918 mmol, 1 eq.), ethyl (4-(2-methoxyvinyl)phenyl) carbonate (**B1**, 204 mg, 0.918 mmol, 1 eq.) and TFA (0.69 mL, 9.18 mmol, 10 eq.) were used. The reaction was completed after 16 h and the crude product was purified by FCC using hexanes/acetone (acetone up to 20%) yielding the desired tetrahydroisoquinoline (**5h**, 189 mg, 0.399 mmol, 44%) as a colorless solid. mp: 124 °C; ^{1}H NMR (400 MHz, $C_2D_2Cl_4$, 100 °C): δ (ppm) = 7.11 – 6.96 (m, 4H, 2‘-H, 3’H, 5‘-H, 6‘-H), 6.05 (s, 1H, 8-H), 5.14 (t, J = 6.8 Hz, 1H, 1-H), 4.26 (q, J = 7.1 Hz, 2H, OCOOCH̲2CH3), 3.97 (d, J = 39.7 Hz, 3H, NCOOCH̲2CH3, 3-H), 3.81 (s, 3H, 5-OCH3), 3.80 (s, 3H, 6-OCH3), 3.62 (s, 3H, 7-OCH3), 3.27 (ddd, J = 13.6, 9.3, 5.0 Hz, 1H, 3-H), 3.07 (dd, J = 13.5, 6.7 Hz, 1H, α-H), 2.94 (dd, J = 13.5, 7.0 Hz, 1H, α-H), 2.71 (ddd, J = 15.6, 9.3, 6.0 Hz, 1H, 4-H), 2.58 (dd, J = 16.4, 4.8 Hz, 1H, 4-H), 1.34 (t, J = 7.1 Hz, 3H, OCOOCH2CH̲3), 1.16 (t, J = 7.1 Hz, 3H, NCOOCH2CH̲3); ^{13}C NMR (101 MHz, $C_2D_2Cl_4$, 100 °C): δ (ppm) = 155.7 (N-CO), 153.6 (O-CO), 151.9 (C-7), 151.2 (C-5), 150.3 (C-4‘), 141.5 (C-6), 136.4 (C-1‘), 131.9 (C-8a), 130.8 (C-2‘, C-6‘), 121.2 (C-4a), 120.9 (C-3‘, C-5‘), 107.6 (C-8), 64.9 (OCOOC̲H2CH3), 61.5 (NCOOC̲H2CH3), 61.0 (6-OCH3), 60.7 (5-OCH3), 56.6 (7-OCH3), 56.5 (C-1), 42.6 (C- α), 38.5 (C-3), 22.5 (C-4), 14.8 (NCOOCH2C̲H3), 14.4 (OCOOCH2C̲H3); IR (ATR): $\tilde{\nu}_{max}$ [cm^{-1}] = 2979, 1755, 1674, 1465, 1282, 1212, 1101,

1035, 987, 851, 763; HR-MS (ESI): calcd. for $C_{25}H_{31}NO_8$: 474.2128 $[M+H]^+$, found 474.2124; purity (HPLC): >99%.

(±)-4-((6,7-Dimethoxy-2-methyl-1,2,3,4-tetrahydroisoquinolin-1-yl)methyl) phenol (*rac*-Armepavine) (2a)

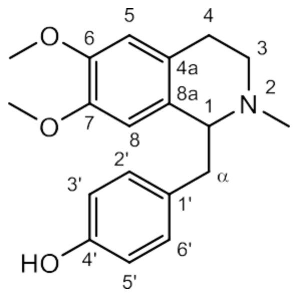

Synthesis and characterization of compound **2a** was reported previously in [1]. However, this published method gave **2a** in only 35 % yield, in a mixture with a by-product.

In a manner similar to a procedure in [1] and following general procedure D, $LiAlH_4$ (124 mg, 3.27 mmol, 11 equiv) and (±)-ethyl 1-(4-((ethoxycarbonyl)oxy)benzyl)-6,7-dimethoxy-3,4-dihydroisoquinoline-2(1*H*)-carboxylate (**5a**, 132 mg, 0.298 mmol, 1 equiv) were used. The reaction was completed after 3 h and the crude product was purified by FCC using CH_2Cl_2/C_2H_5OH (C_2H_5OH up to 10%) yielding *rac*-armepavine (**2a**, 57 mg, 0.183 mmol) as a colorless solid (yield 62%). mp: 162 °C; ^1H NMR (400 MHz, methanol-d_4): δ (ppm) = 6.88 (d, J = 8.5 Hz, 2H, 2'-H, 6'-H), 6.70 (d, J = 8.4 Hz, 2H, 3'-H, 5'-H), 6.66 (s, 1H, 5-H), 5.84 (s, 1H, 8-H), 3.76 (s, 3H, 6-OCH_3), 3.73 (dd, J = 9.5, 4.4 Hz, 1H, 1-H), 3.42 (s, 3H, 7-OCH_3), 3.28 – 3.19 (m, 1H, 3-H), 3.16 (dd, J = 13.2, 4.3 Hz, 1H, α-H), 2.97 – 2.85 (m, 1H, 4-H), 2.84 – 2.74 (m, 1H, 3-H), 2.66 (dd, J = 13.3,9.5 Hz, 2H, α-H, 4-H), 2.53 (s, 3H, NCH_3); ^{13}C NMR (101 MHz, methanol-d_4): δ (ppm)= 157.0 (C-4'), 149.0 (C-6), 147.5 (C-7), 132.0 (C-2', C-6'), 131.3 (C-1'), 129.8 (C-8a), 126.5 (C-4a), 116.1 (C-3', C-5'), 113.0 (C-8), 112.9 (C-5), 66.0 (C-1), 56.3 (6-OCH_3), 55.9 (7-OCH_3), 47.0 (C-3), 42.4 (NCH_3), 40.0 (C-α), 26.0 (C-4); IR (ATR): $\tilde{\nu}_{max}$ [cm^{-1}] = 2939, 2581, 1613, 1514, 1460, 1362, 1246, 1228, 1115, 1023, 862, 841, 791, 765, 745, 663; HR-MS (ESI): calcd. for $C_{19}H_{23}NO_3$: 314.1756 $[M+H]^+$, found 314.1752; purity (HPLC): >99%.

(±)-4-((6,7-Dimethoxy-1,2,3,4-tetrahydroisoquinolin-1-yl)methyl) phenol (*rac*-Norarmepavine) (2b)

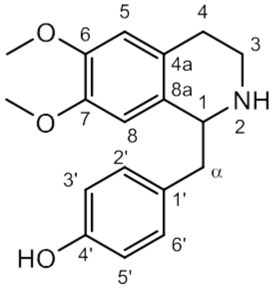

According to Sawyer et al. [8], 1.62 mL (2.59 mmol, 5 equiv) methyllithium (1.6 M in diethyl ether) was added dropwise to a solution of (±)-ethyl 1-(4- ((ethoxycarbon-yl)oxy)benzyl)-6,7-dimethoxy-3,4-dihydroisoquinoline-2(1*H*)- carboxylate (**5a**, 230 mg, 0.519 mmol, 1 equiv) in 2 mL dry THF at 0 °C. The reaction mixture was stirred for 1 h, followed by careful dilution with 50 mL saturated ammonium chloride solution. This was extracted with CH_2Cl_2 (2 × 50 mL) and the combined organic layers were washed once with brine and dried over Na_2SO_4 and filtered. The filtrate was concentrated *in vacuo*. Purification was accomplished by FCC using CH_2Cl_2/C_2H_5OH (C_2H_5OH up to 5%) yielding *rac*-norarmepavine (**2b**, 54 mg, 0.18 mmol, 35%) as a colorless solid. mp: 85 °C; ^{1}H NMR (400 MHz, methanol-d_4): δ (ppm) = 7.15 – 7.00 (m, 2H, 2'-H, 6'-H), 6.79 – 6.71 (m, 2H, 3'-H, 5'-H), 6.67 (s, 1H, 5-H), 6.59 (s, 1H, 8-H), 4.08 (dd, J = 8.5, 5.6 Hz, 1H, 1-H), 3.79 (s, 3H, 6-OCH_3), 3.70 (s, 3H, 7-OCH_3), 3.21 (dt, J = 12.1, 6.0 Hz, 1H, 3-H), 3.11 (dd, J = 13.7, 5.6 Hz, 1H, α-H), 2.95 – 2.80 (m, 2H, 3-H, α-H), 2.75 (t, J = 6.0 Hz, 2H, 4-H); ^{13}C NMR (101 MHz, methanol-d_4): δ (ppm) = 157.3 (C-4'), 149.1 (C-6), 148.4 (C-7), 131.6 (C-2', C-6'), 131.1 (C-1'), 130.6 (C-8a), 128.2 (C-4a), 116.5 (C-3', C-5'), 113.3 (C-5), 111.6 (C-8), 58.0 (C-1), 56.4 (6-OCH_3, 7-OCH_3), 42.4 (C-α), 41.0 (C-3), 29.5 (C-4); IR (ATR): $\tilde{\nu}_{max}$ [cm^{-1}] = 2917, 1611, 1513, 1462, 1449, 1255, 1221, 1109, 1029, 834, 795; HR-MS (ESI): calcd. for $C_{18}H_{21}NO_3$: 300.1600 $[M+H]^+$, found 300.1595; purity (HPLC): >96%.

(±)-5-((6,7-Dimethoxy-2(1*H*)-methyl-1,2,3,4-tetrahydroisoquinolin-1-yl)methyl)-2-methoxyphenol (*rac*-Laudanine) (2c)

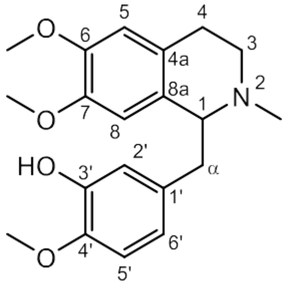

Following general procedure D, $LiAlH_4$ (139 mg, 3.67 mmol, 11 equiv) and (±)-ethyl 1-(3- ((ethoxycarbonyl)oxy)-4-methoxybenzyl)-6,7-dimethoxy-3,4-dihydroisoquinoline-2(1*H*)-carboxylate (**5b**, 158 mg, 0.334 mmol, 1 equiv) were used. The reaction was completed after 3 h and the crude product was purified by FCC using CH_2Cl_2/C_2H_5OH (C_2H_5OH 0% up to 15%) yielding *rac*-laudanine (**2c**, 55 mg, 0.137 mmol, 47%) as a light-yellow solid. mp: 159 °C; ^1H NMR (500 MHz, methanol-d_4): δ (ppm) = 6.83 (d, J = 8.2 Hz, 1H, 5'-H), 6.66 (s, 1H, 5-H), 6.56 (d, J = 2.1 Hz, 1H, 2'-H), 6.52 (dd, J = 8.2, 2.1 Hz, 1H, 6'-H), 5.90 (s, 1H, 8-H), 3.82 (s, 3H, 4'-OCH_3), 3.77 (m, 4H, 6-OCH_3, 1-H), 3.44 (s, 3H, 7-OCH_3), 3.21 (m, 1H, 3-H), 3.14 (dd, J = 13.2, 4.3 Hz, 1H, α-H), 2.91 (m, 1H, 4-H), 2.79 (m, 1H, 3-H), 2.73 – 2.65 (m, 1H, 4-H), 2.63 (dd, J = 13.2, 9.3 Hz, 1H, α -H), 2.53 (s, 3H, NCH_3); ^{13}C NMR (126 MHz, methanol-d_4): δ (ppm) = 149.2 (C-6), 147.7 (C-4'), 147.6 (C-3'), 147.5 (C-7), 133.5 (C-1'), 129,0 (C-8a) 126.4 (C-4a), 122.2 (C-6'), 118.1 (C-2'), 113.0 (C-5'), 112.9 (C-5), 112.9 (C-8), 66.0 (C-1), 56.5 (7-OCH_3), 56.3 (6-OCH_3), 55.9 (4'-OCH_3), 47.1 (C-3), 42.4 (NCH_3), 40.3 (C-α), 26.0 (C-4); IR (ATR): $\tilde{\nu}_{max}$ [cm^{-1}] = 2948, 1507, 1349, 1277, 1248, 1217, 1110, 1026, 937, 859, 809, 785, 748, 668; HR-MS (ESI): calcd. for $C_{20}H_{25}NO_4$: 344.1862 $[M+H]^+$, found 344.1859; purity (HPLC): >97%.

(±)-4-((6,7-Dimethoxy-2-methyl-1,2,3,4-tetrahydroisoquinolin-1-yl)methyl)-2-methoxyphenol (*rac*-Pseudocodamine) (2d)

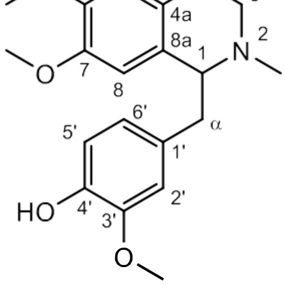

Following general procedure D, $LiAlH_4$ (194 mg, 5,11 mmol, 11 equiv) and (±)-ethyl 1-(4- ((ethoxycarbonyl)oxy)-3-methoxybenzyl)-6,7-dimethoxy-3,4-dihydroisoquinoline-2(1*H*)-carboxylate (**5c**, 220 mg, 0.465 mmol, 1 equiv) were used. The reaction was completed after 3 h and the crude product was purified by FCC using CH_2Cl_2/C_2H_5OH (C_2H_5OH 0% up to 15%) yielding *rac*-pseudocodamine (**2d**, 110 mg, 0.32 mmol, 68%) as a light yellow solid. mp: 132 °C; ^1H NMR (500 MHz, methanol-d_4): δ (ppm) = 6.71 (d, J = 8.5 Hz, 1H, 5'-H), 6.67 (s, 1H, 5-H), 6.58 – 6.51 (m, 2H, 2'-H, 6'-H), 5.89 (s, 1H, 8-H), 3.75 (m, 7H, 3'-OCH_3, 1-H, 6-OC$\underline{H}_3$), 3.44 (s, 3H, 7-OCH_3), 3.25 – 3.13 (m, 2H, 3-H, α-H), 2.91 (m, 1H, 4-H), 2.82 – 2.74 (m, 1H, 3-H), 2.73 – 2.62 (m, 2H, α-H, 4-H), 2.54 (s, 3H, O-CH_2-O); ^{13}C NMR (126 MHz, methanol-d_4): δ (ppm) = 149.1 (C-3'), 148.8 (C-6), 147.5 (C-7), 146.1 (C-4'), 132.0 (C-1'), 129.8 (C-8a), 126.7 (C-4a), 123.6 (C-6'), 116.1 (C-5'), 114.9 (C-2'), 113.1 (C-8), 112.9 (C-5), 65.9 (C-1), 56.4 (3'-OCH_3), 56.3 (6-OCH_3), 55.9 (7-OCH_3), 47.1 (C-3), 42.5 (NCH_3), 40.4 (C-α), 26.1 (C-4); IR (ATR): $\tilde{v}_{max}$ [cm^{-1}] = 2930, 1595, 1508, 1451, 1362, 1247, 1209, 1129, 1109, 1014, 870, 831, 797, 735, 669; HR-MS (ESI): calcd. for $C_{20}H_{25}NO_4$: 344.1862 $[M+H]^+$, found 344.1858; purity (HPLC): >99%.

(±)-1-(3-Hydroxy-4-methoxybenzyl)-6-methoxy-2-methyl-1,2,3,4-tetrahydroisoquinolin-7-ol (*rac*-Reticuline) (2e)

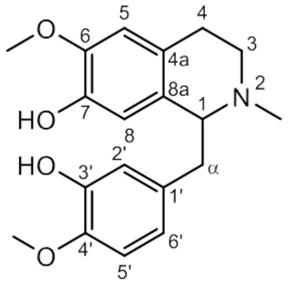

Following general procedure D, $LiAlH_4$ (37 mg, 0.98 mmol, 11 equiv) and (±)-ethyl-1-(3-((ethoxycarbonyl)oxy)-4-methoxybenzyl)-7-hydroxy-6-methoxy-3,4-dihydroisoquin-oline-2(1*H*)-carboxylate (**5d**, 41 mg, 0.089 mmol, 1 equiv) were used. The reaction was completed after 3 h and the crude product was purified by FCC using CH_2Cl_2/C_2H_5OH (C_2H_5OH 0% up to 20%) yielding *rac*-reticuline (**2e**, 18 mg, 0.056 mmol, 63%) as a yellow solid. mp: 76 °C; ^1H NMR (400 MHz, methanol-d_4): δ (ppm) = 6.80 (d, J = 8.2 Hz, 1H, 5'-H), 6.63 (s, 1H, 5-H), 6.61 (d, J = 2.1 Hz, 1H, 2'-H), 6.52 (dd, J = 8.1, 2.1 Hz, 1H, 6'-H), 6.13 (s, 1H, 8-H), 3.82 (s, 3H, 4'-OCH_3), 3.79 (s, 3H, 6-

OCH_3), 3.69 (t, *J* = 7.5, 1H, 1-H), 3.15 (ddd, *J* = 12.6, 9.3, 5.4 Hz, 1H, 3-H), 3.04 (dd, *J* = 13.8, 5.0 Hz, 1H, α-H), 2.85 (ddd, *J* = 15.8, 9.4, 6.1 Hz, 1H, 4-H), 2.76 – 2.59 (m, 3H, 3-H. α-H, 4-H), 2.46 (s, 3H, NCH_3); ^{13}C NMR (101 MHz, methanol-d_4): δ (ppm) = 147.9 (C-6), 147.5 (C-3'), 147.3 (C-4'), 145.1 (C-7), 133.8 (C-1'), 130.6 (C-8a), 125.4 (C-4a), 121.9 (C-6'), 117.6 (C-2'), 115.7 (C-8), 112.7 (C-5'), 112.6 (C-5), 65.9 (C-1), 56.4 (6-OCH_3), 56.3 (4'-OCH_3), 47.6 (C-3), 42.6 (NCH_3), 41.0 (C-α), 26.0 (C-4); IR (ATR): $\tilde{\nu}_{max}$ [cm^{-1}] = 2934, 1590, 1508, 1441, 1370, 1272, 1216, 1129, 1024, 869, 761; HR-MS (ESI): calcd. for $C_{19}H_{23}NO_4$: 330.1705 $[M+H]^+$, found 330.1702; purity (HPLC): >99%.

(±)-1-(4-Hydroxy-3-methoxybenzyl)-6-methoxy-2-methyl-1,2,3,4-tetrahydroisoquinolin-7-ol (*rac*-Orientaline) (2f)

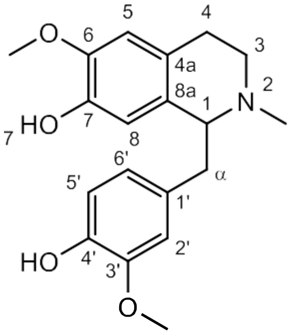

Following general procedure D, $LiAlH_4$ (157 mg, 4.14 mmol, 11 equiv) and (±)-ethyl 1-(4-((ethoxycarbonyl)oxy)-3-methoxybenzyl)-7-hydroxy-6-methoxy-3,4-dihydroisoquinoline-2(1*H*)-carboxylate (**5e**, 173 mg, 0.376 mmol, 1 equiv) were used. The reaction was completed after 3 h and the crude product was purified by FCC using CH_2Cl_2/ C_2H_5OH (C_2H_5OH up to 20%) yielding *rac*-orientaline (**2f**, 77 mg, 0.234 mmol, 62%) as a yellow solid. mp: 74 °C; ^1H NMR (500 MHz, methanol-d_4): δ (ppm) = 6.69 (d, *J* = 7.9 Hz, 1H, 5'-H), 6.64 (s, 1H, 5-H), 6.59 – 6.52 (m, 2H, 6'-H, 2'-H), 6.13 (s, 1H, 8-H), 3.80 (s, 3H, 6-OCH_3), 3.74 - 3.70 (m, 4H, 1-H, 3'-OCH_3), 3.15 (ddd, *J* = 12.5, 9.4, 5.5 Hz, 1H, 3-H), 3.08 (dd, *J* = 13.7, 4.8 Hz, 1H, α-H), 2.92 – 2.69 (m, 3H, 4-H, α-H, 3-H), 2.63 (dt, *J* = 16.3, 4.6 Hz, 1H, 4-H), 2.49 (s, 3H, NCH_3); ^{13}C NMR (101 MHz, methanol-d_4): δ (ppm) = 148.6 (C-3'), 147.9 (C-6), 145.9 (C-4'), 145.1 (C-7), 132.0 (C-1'), 130.2 (C-8a), 125.5 (C-4a), 123.3 (C-6'), 116.0 (C-8), 115.9 (C-5'), 114.5 (C-2'), 112.6 (C-5), 65.9(C-1), 56.3 (3'-OCH_3), 56.3 (6-OCH_3), 47.6 (C-3), 42.5 (NCH_3), 41.0 (C-α), 26.0 (C-4); IR (ATR): $\tilde{\nu}_{max}$ [cm^{-1}] = 2935, 1732, 1599, 1511, 1447, 1370, 1270,

1153, 1123, 1029, 801; HR-MS (ESI): calcd. for $C_{19}H_{23}NO_4$: 330.1705 $[M+H]^+$, found 330.1702; purity (HPLC): >91%.

(±)-4-((6-Methyl-5,6,7,8-tetrahydro-[1,3]dioxolo[4,5-*g*]isoquinolin-5-yl)methyl) phenol (*rac*-Cinnamolaurine) (3a)

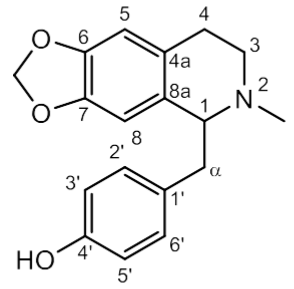

Following general procedure D, $LiAlH_4$ (78 mg, 2.06 mmol, 11 equiv) and (±)-ethyl 5-(4-((ethoxycarbonyl)oxy)benzyl)-7,8-dihydro-[1,3]dioxolo[4,5-*g*]isoquinoline-6(5*H*)-carboxylate (**5f**, 80.0 mg, 0.187 mmol, 1 equiv) were used. The reaction was completed after 3 h and the crude product was purified by FCC using CH_2Cl_2/ C_2H_5OH (C_2H_5OH up to 15%) yielding *rac*-cinnamolaurine (**3a**, 37 mg, 0.126 mmol) as a colorless solid (yield 67%). mp: 169 °C ; ^1H NMR (400 MHz, methanol-d_4): δ (ppm) = 6.90 (d, J = 8.5 Hz, 2H, 2'-H, 6'-H), 6.69 (d, J = 8.5 Hz, 2H, 3'-H, 5'-H), 6.55 (s, 1H, 5-H), 6.00 (s, 1H, 8-H), 5.81 (dd, J = 8.5, 1.2 Hz, 2H, O-CH_2-O), 3.72 (dd, J = 7.9, 5.0 Hz, 1H, 1-H), 3.17 (ddd, J = 12.6, 9.5, 5.5 Hz, 1H, 3-H), 3.08 (dd, J = 13.7, 5.0 Hz, 1H, α-H), 2.86 (ddd, J = 16.1, 9.6, 6.4 Hz, 1H, 4-H), 2.79 – 2.68 (m, 2H, α-H, 3-H), 2.62 (ddd, J = 16.4, 5.6, 3.6 Hz, 1H, 4-H), 2.48 (s, 3H, NCH_3); ^{13}C NMR (101 MHz, methanol-d_4): δ (ppm) = 156.9 (C-4'), 147.7 (C-6), 146.6 (7), 131.7 (C-2', C-6'), 131.3 (C-1'), 131.0 (C-8a), 127.6 (C-4a), 116.1 (C-3', C-5'), 109.2 (C-5), 109.0 (C-8), 101.9 (O-CH_2-O), 66.5 (C-1), 47.1 (C-3), 42.5 (NCH_3), 40.7 (C-α), 26.4 (C-4); IR (ATR): $\tilde{\nu}_{max}$ [cm^{-1}] = 2957, 1480, 1384, 1275, 1255, 1237, 1107, 1034, 939, 920, 871, 829, 770, 699; HR-MS (ESI): calcd. for $C_{18}H_{19}NO_3$: 298.1443 $[M+H]^+$, found 298.1439; purity (HPLC): >97%.

(±)-4-((5,6,7,8-Tetrahydro-[1,3]dioxolo[4,5-g]isoquinolin-5-yl)methyl) phenol (*rac*- Norcinnamolaurine) (3b)

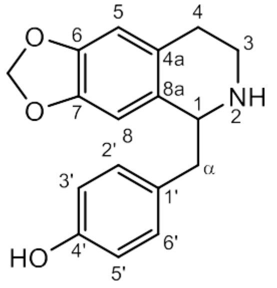

According to Sawyer et al. [8], (±)-ethyl 5-(4-((ethoxycarbonyl)oxy)benzyl)-7,8-dihydro-[1,3]dioxolo[4,5-*g*]isoquinoline-6(5*H*)-carboxylate (**5f**, 90.0 mg, 0.187 mmol, 1 equiv) was dissolved in 2 mL dry THF and a solution of methyllithium (1.6 M in diethyl ether, 0.66 mL; 1.1 mmol) was added dropwise at 0 °C. The reaction mixture was stirred for 1 h, followed by careful dilution with 50 mL saturated ammonium chloride solution. This was extracted with CH_2Cl_2 (2 × 50 mL) and the combined organic layers were washed once with brine and dried over Na_2SO_4, filtered and concentrated *in vacuo*. Purification was accomplished by FCC using CH_2Cl_2/C_2H_5OH (C_2H_5OH up to 10%) yielding *rac*-norcinnamolaurine (**3b**, 22 mg, 0.077 mmol, 37%) as a colorless solid. mp: 190 °C. ^{1}H NMR (400 MHz, methanol-d_4): δ (ppm) = 7.13 – 7.01 (m, 2H, 2'-H, 6'-H), 6.78 – 6.72 (m, 2H, 3'-H, 5'-H), 6.69 (s, 1H, 5-H), 6.57 (s, 1H, 8-H), 5.87 (m, 2H, O-CH_2-O), 4.04 (dd, J = 9.4, 4.5 Hz, 1H, 1-H), 3.22 - 3.06 (m, 2H, α-H, 3-H), 2.89 – 2.76 (m, 2H, 3-H, α-H), 2.72 (t, J = 5.7 Hz, 2H, 4-H); ^{13}C NMR (101 MHz, methanol-d_4): δ (ppm) = 157.3 (C-4'), 147.7 (C-7), 147.4 (C-6), 131.9 (C-4a), 131.4 (C-2', C-6'), 130.3 (C-1'), 129.0 (C-8a), 116.5 (C-3', C-5'), 109.5 (C-8), 107.5 (C-5), 102.0 (O-CH_2-O), 58.4 (C-1), 42.3 (C-α), 40.9 (C-3), 30.0 (C-4); IR (ATR): $\tilde{\nu}_{max}$ [cm^{-1}] = 2921, 1609, 1514, 1483, 1428, 1388, 1241, 1195, 1101, 1035, 963, 931, 854, 799, 743, 685; HR-MS (ESI): calcd. for $C_{17}H_{17}NO_3$: 284.1287 [M+H]$^+$, found 284.1283; purity (HPLC): >99%.

(±)-2-Methoxy-5-((6-methyl-5,6,7,8-tetrahydro-[1,3]dioxolo[4,5-*g*]isoquinolin-5-yl)methyl) phenol (*rac*-*N*-Demethylphyllocryptine) (3c)

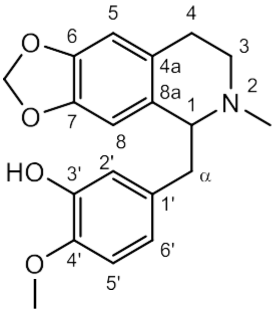

Following general procedure D, $LiAlH_4$ (100 mg, 2.64 mmol, 11 equiv) and (±)-ethyl 5-(3- ((ethoxycarbonyl)oxy)-4-methoxybenzyl)-7,8-dihydro-[1,3]dioxolo[4,5-*g*]isoquin-oline- 6(5*H*)-carboxylate (**5g**, 110 mg, 0.24 mmol, 1 equiv) were used. The reaction was completed after 3 h and the crude product was purified by FCC using CH_2Cl_2/ C_2H_5OH (C_2H_5OH 0% up to 15%) yielding *rac*-*N*-demethylphyllocryptine (**3c**, 37 mg, 0.113 mmol, 47%) as a light yellow solid. mp: 121 °C; ^1H NMR (500 MHz, methanol-d_4): δ (ppm) = 6.80 (d, J = 8.2 Hz, 1H, 5'-H), 6.59 (d, J = 2.2 Hz, 1H, 2'-H), 6.56 – 6.50 (m, 2H, 5-H, 6'-H), 6.03 (s, 1H, 8-H), 5.80 (dd, J = 9.5, 1.2 Hz, 2H, O-CH_2-O), 3.82 (s, 3H, 4'-OCH_3), 3.72 (dd, J = 7.8, 5.0 Hz, 1H, 1-H), 3.15 (m, 1H, 3-H), 3.05 (dd, J = 13.6, 5.0 Hz, 1H, α-H), 2.84 (m, 1H, 4-H), 2.76 – 2.58 (m, 3H, 3-H, α-H, 4-H), 2.46 (s, 3H, NCH_3); ^{13}C NMR (126 MHz, methanol-d_4): δ (ppm) = 147.7 (C-4'), 147.6 (C-7), 147.4 (C-3'), 146.7 (C-6), 133.5 (C-1'), 131.1 (C-8a), 127.6 (C-4a), 121.9 (C-6'), 117.7 (C-2'), 112.7 (C-5'), 109.2 (C-3'), 108.9 (C-5), 101.9 (C-8), 66.4 (O-CH_2-O), 56.5 (C-1), 47.1 (OCH_3), 42.5 (C-3), 41.0 (NCH_3), 26.4 (C-4); IR (ATR): $\tilde{\nu}_{max}$ [cm^{-1}] = 2916, 2360, 2167, 1592, 1480, 1428, 1228, 1157, 1129, 1035, 921, 867, 819, 693, 664; HR-MS (ESI): calcd. for $C_{19}H_{21}NO_4$: 328.1549 $[M+H]^+$, found 328.1544; purity (HPLC): >93%.

(±)-4-((5,6,7-Trimethoxy-2-methyl-1,2,3,4-tetrahydroisoquinolin-1-yl)methyl) phenol (*rac*-Thalifendlerine) (4)

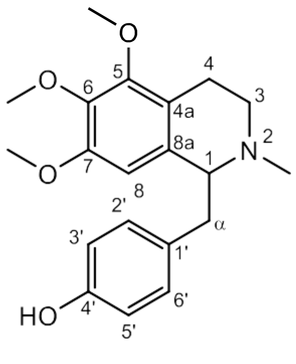

Following general procedure D, $LiAlH_4$ (62.6 mg, 1.65 mmol, 11 equiv) and (±)-ethyl 1-(4-((ethoxycarbonyl)oxy)benzyl)-5,6,7-trimethoxy-3,4-dihydroisoquinoline-2(1*H*)-carboxylate (**5h**, 71 mg, 0,15 mmol, 1 equiv) were used. The reaction was completed after 3 h and the crude product was purified by FCC using hexane/ethyl acetate with 0.75% NEt_3 (ethyl acetate up to 80%) and the second time using CH_2Cl_2/C_2H_5OH (C_2H_5OH up to 15%) yielding *rac*-thalifendlerine (**4**, 42 mg, 0.122 mmol, 82%) as a colorless solid. mp: 174 °C; ^1H NMR (400 MHz, methanol-d_4): δ (ppm) = 6.93 – 6.85

(m, 2H, 2’-H, 6’-H), 6.74 – 6.66 (m, 2H, 3’-H, 5’-H), 5.73 (s, 1H, 8-H), 3.82 (s, 3H, 7-OCH_3), 3.76 (s, 3H, 6-OCH_3), 3.72 (dd, J = 9.2, 4.3 Hz, 1H, 1-H), 3.45 (s, 3H, 5-OCH_3), 3.24 – 3.10 (m, 2H, α-H, 3-H), 2.85 – 2.72 (m, 2H, 4-H), 2.70 – 2.57 (m, 2H, α-H, 3-H), 2.51 (s, 3H, NCH_3); ^{13}C NMR (101 MHz, methanol-d_4): δ (ppm) = 157.0 (C-4’), 152.0 (C-7), 151.9 (C-5), 141.6 (C-6), 133.5 (C-4a), 132.0 (C-2’, C-6’), 131.4 (C-1’), 120.4 (C-8a), 116.1 (C-3’, C-5’), 108.9 (C-8), 66.2 (C-1), 61.2 (6-OCH_3), 60.9 (7-OCH_3), 55.9 (5-OCH_3), 46.4 (C-4), 42.4 (NCH_3), 39.8 (C-α), 20.8 (C-3); IR (ATR): $\tilde{\nu}_{max}$ [cm^{-1}] = 2929, 2513, 1585, 1515, 1494, 1340, 1244, 1107, 1089, 1035, 992, 854, 832, 743; HR-MS (ESI): calcd. for $C_{20}H_{25}NO_4$: 344.1862 $[M+H]^+$, found 344.1858; purity (HPLC): >97%.

^{1}H and ^{13} NMR spectra

Ethyl (5-formyl-2-methoxyphenyl) carbonate

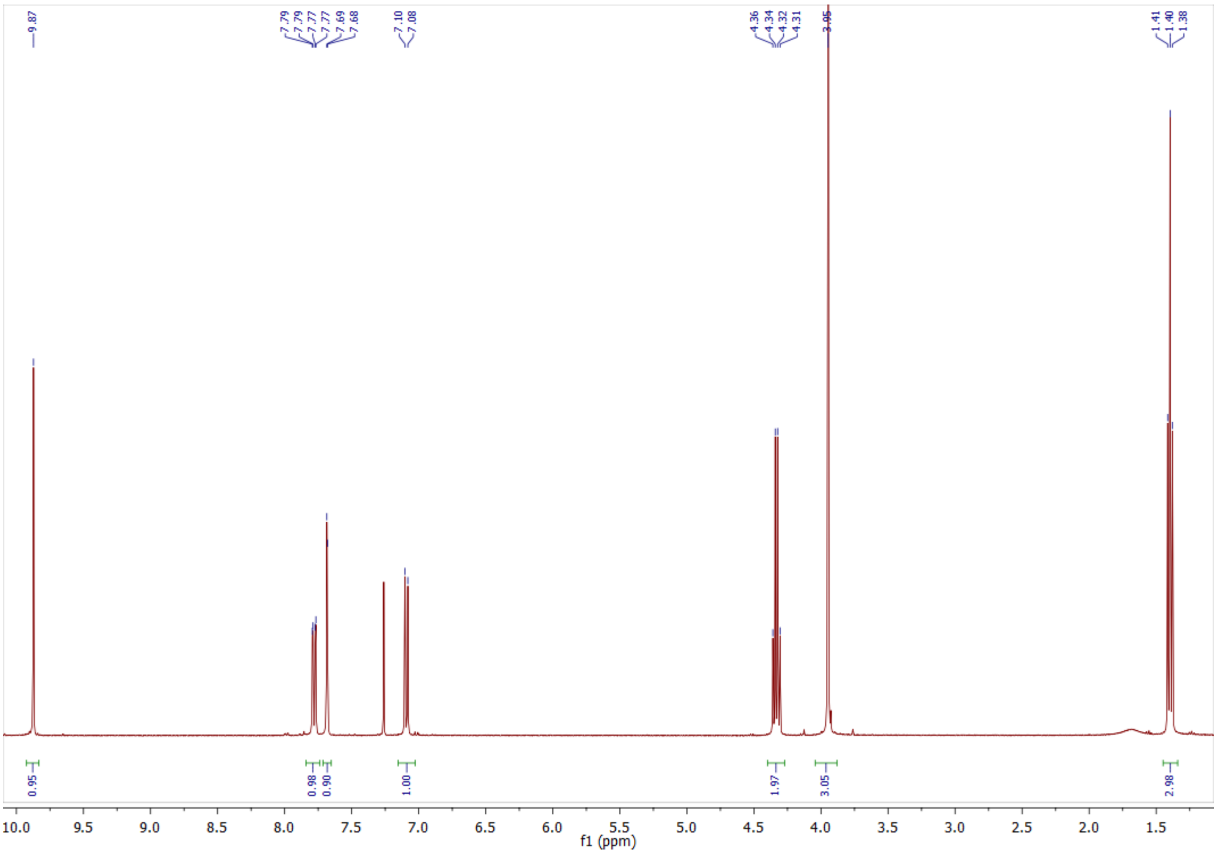

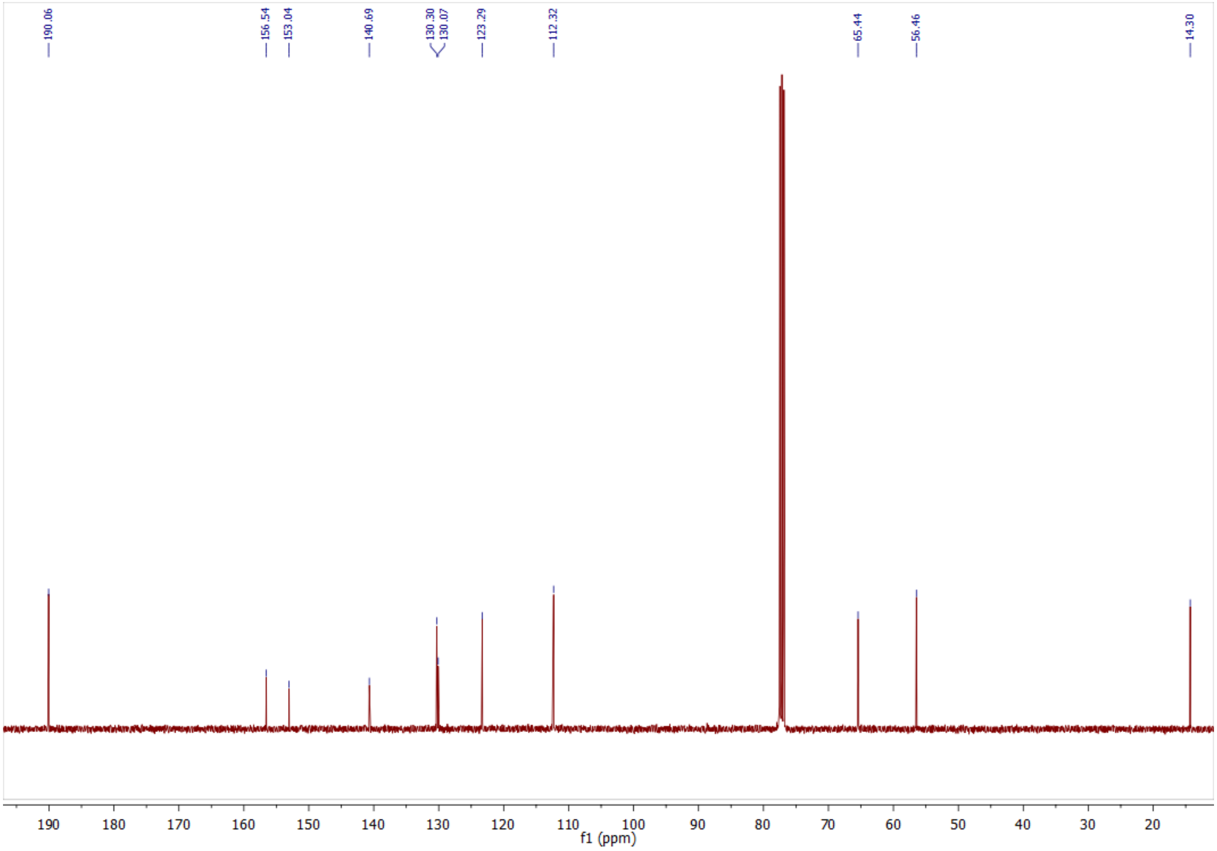

Ethyl (4-formyl-2-methoxyphenyl) carbonate

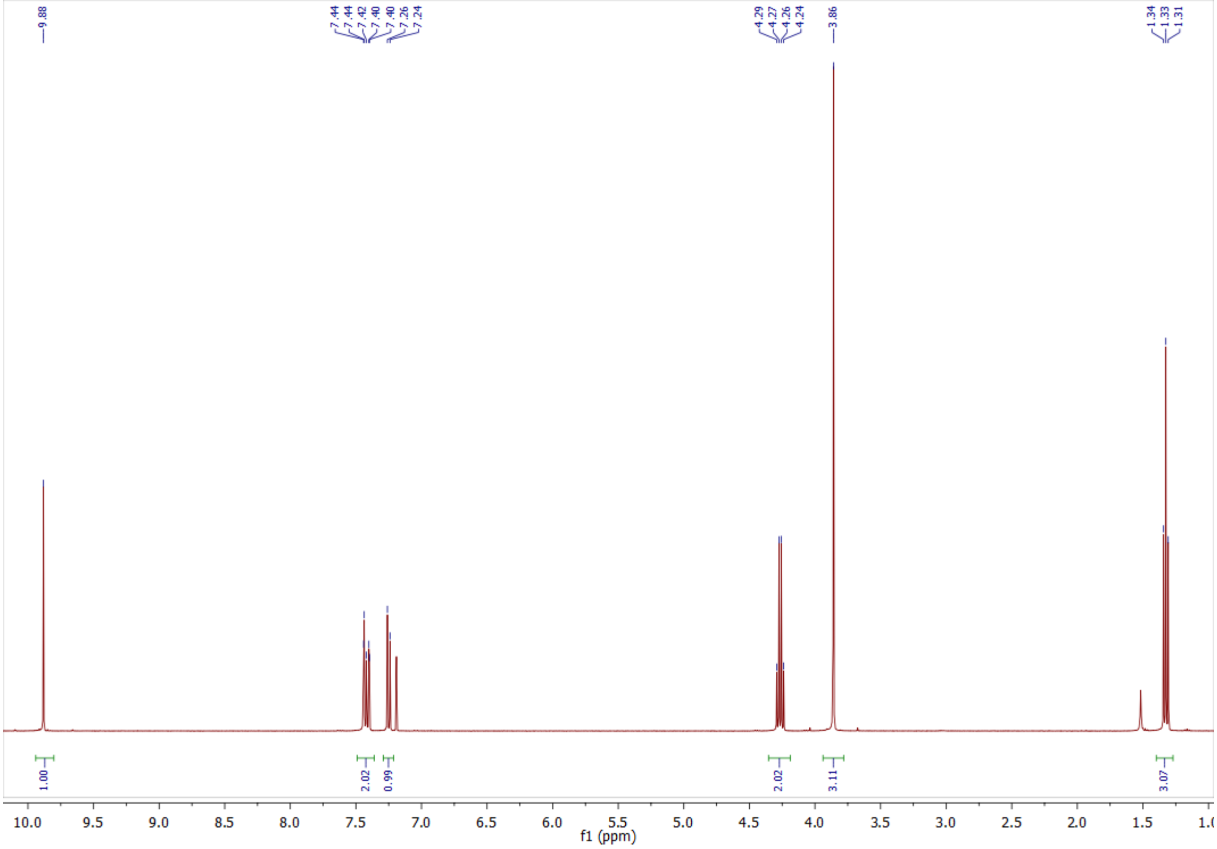

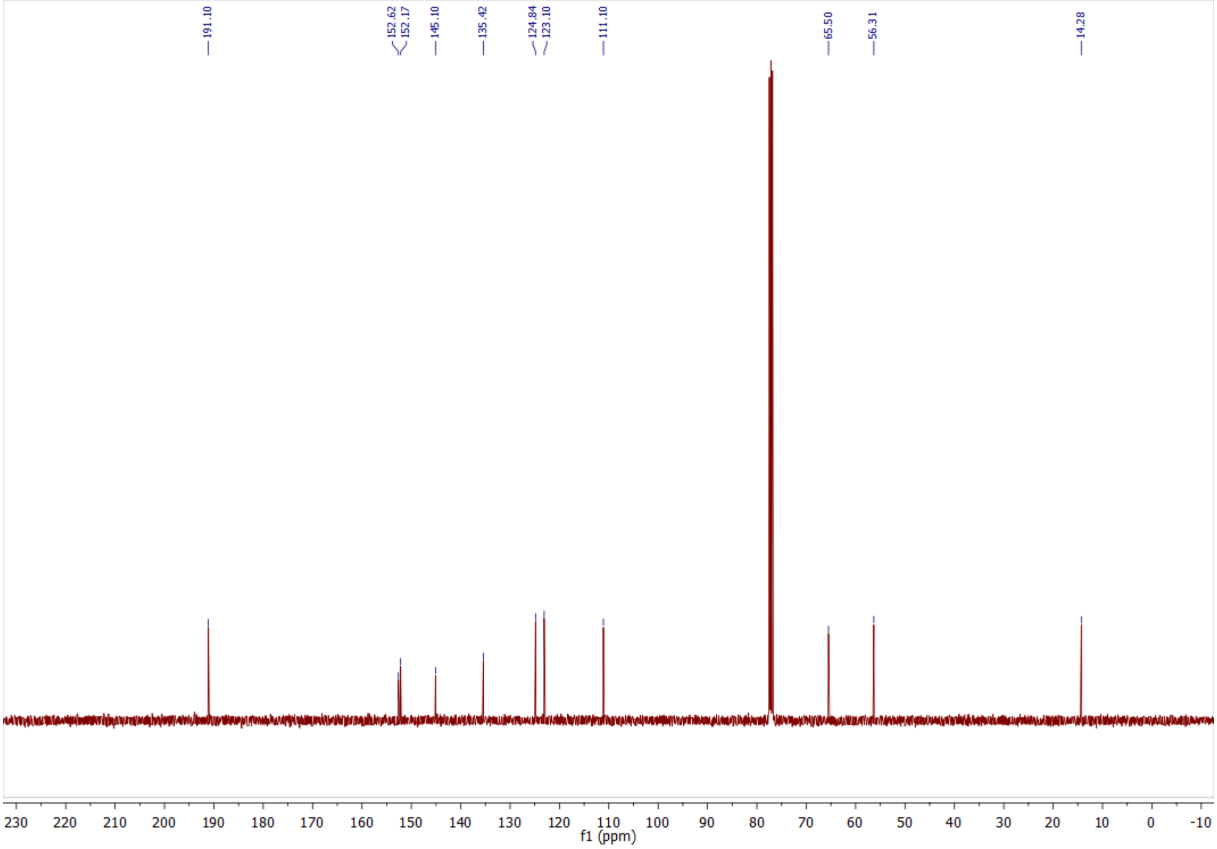

Ethyl (2-methoxy-5-(2-methoxyvinyl)phenyl) carbonate (**B2**, *E*/*Z* mixture)

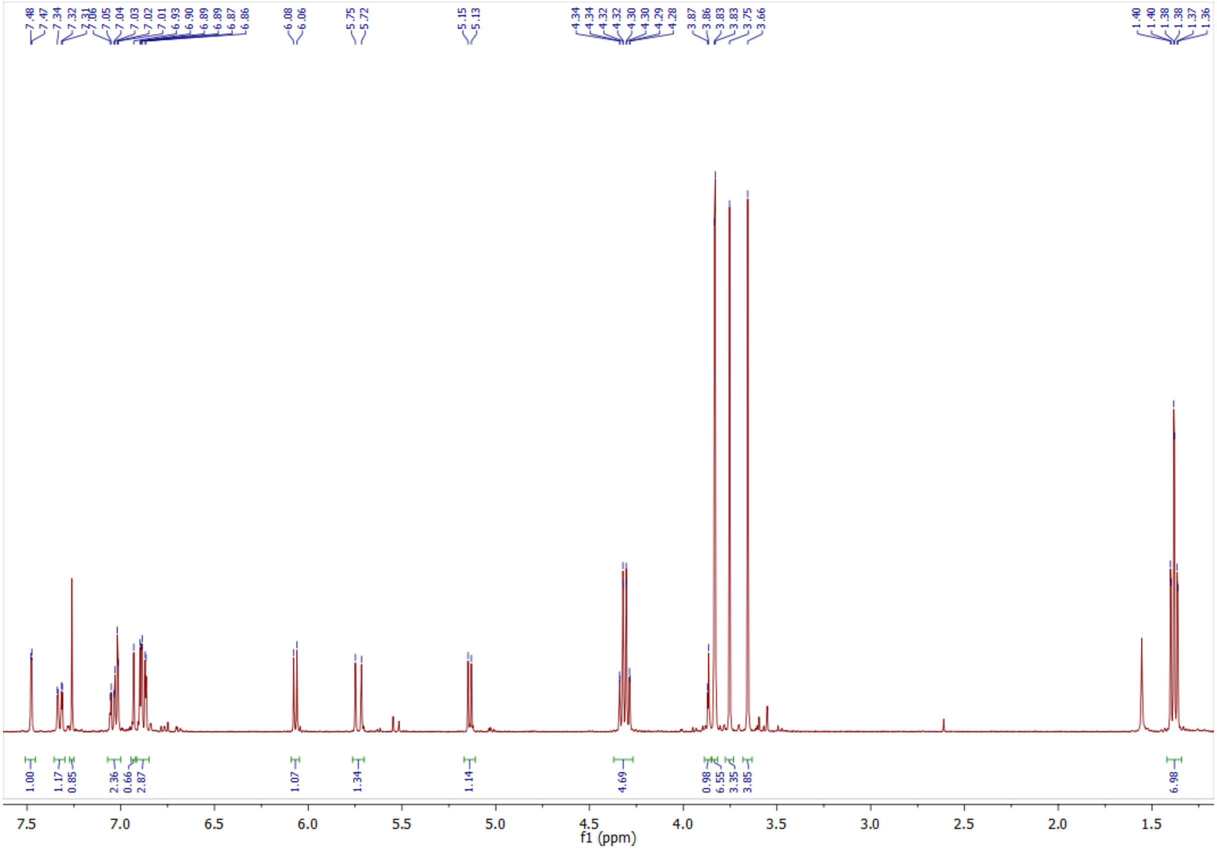

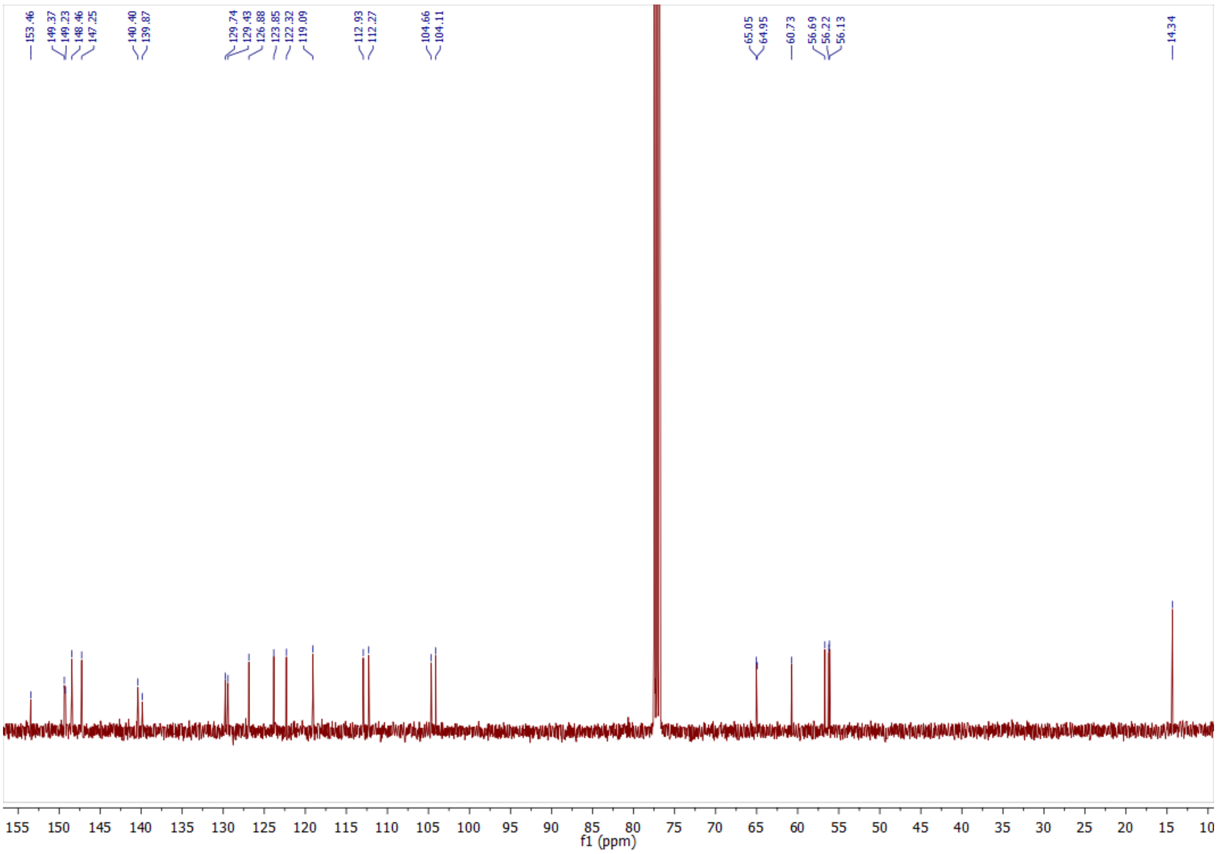

Ethyl (2-methoxy-4-(2-methoxyvinyl)phenyl) carbonate (**B3**, *E*/*Z* mixture)

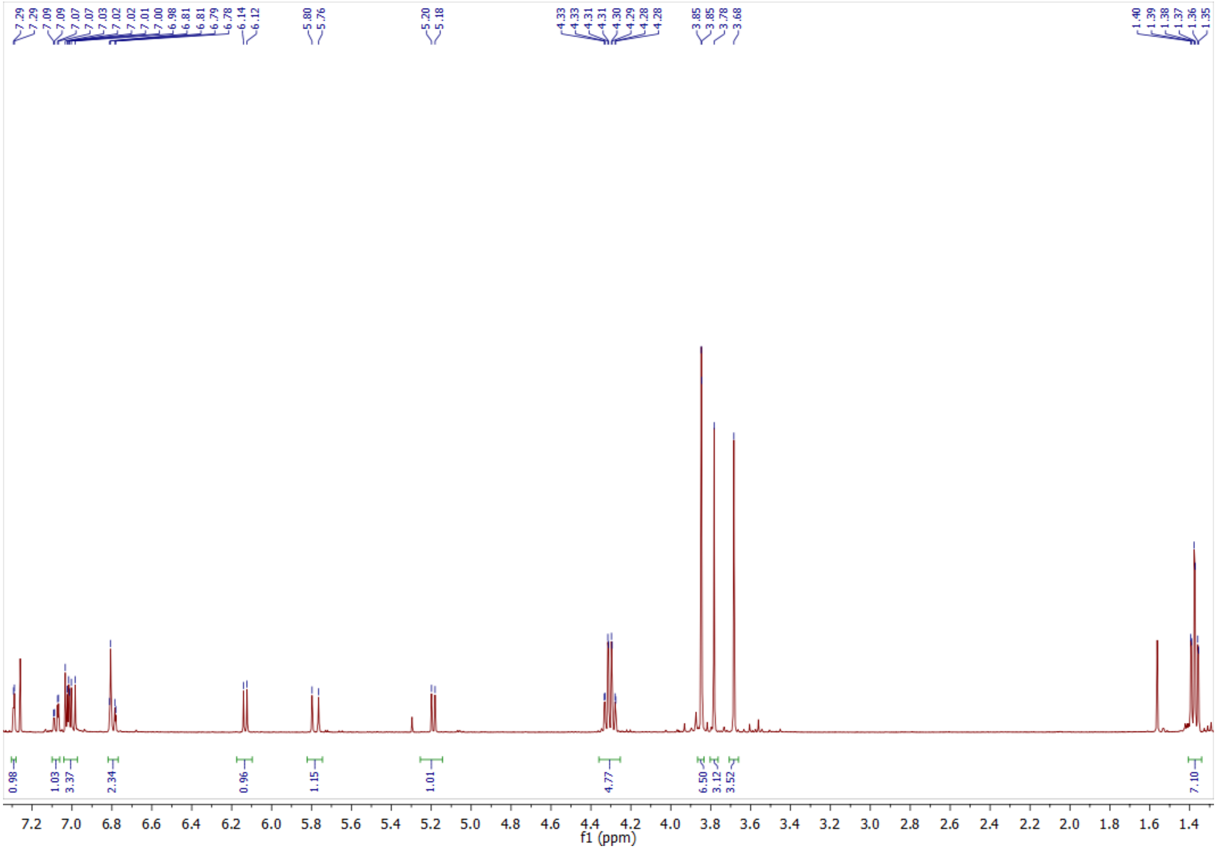

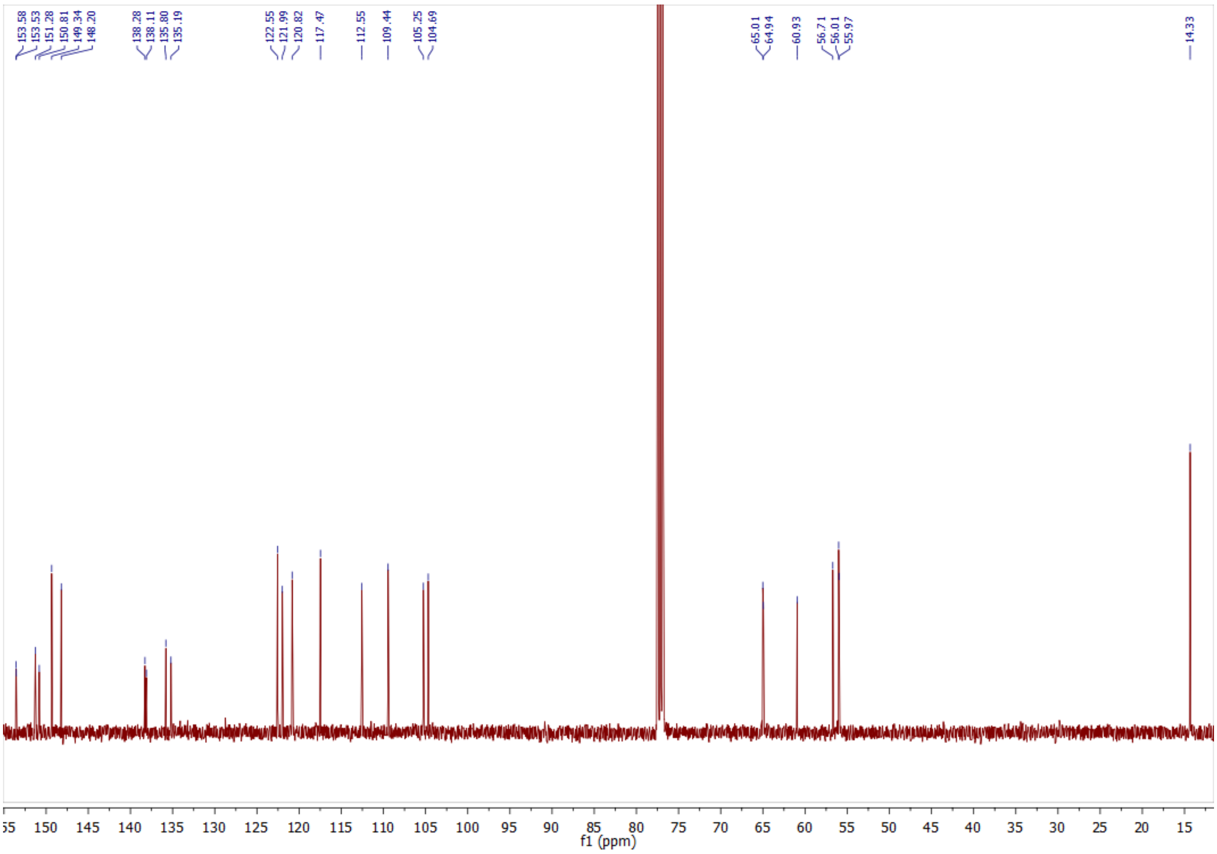

2-(2,3,4-Trimethoxyphenyl)ethan-1-amine

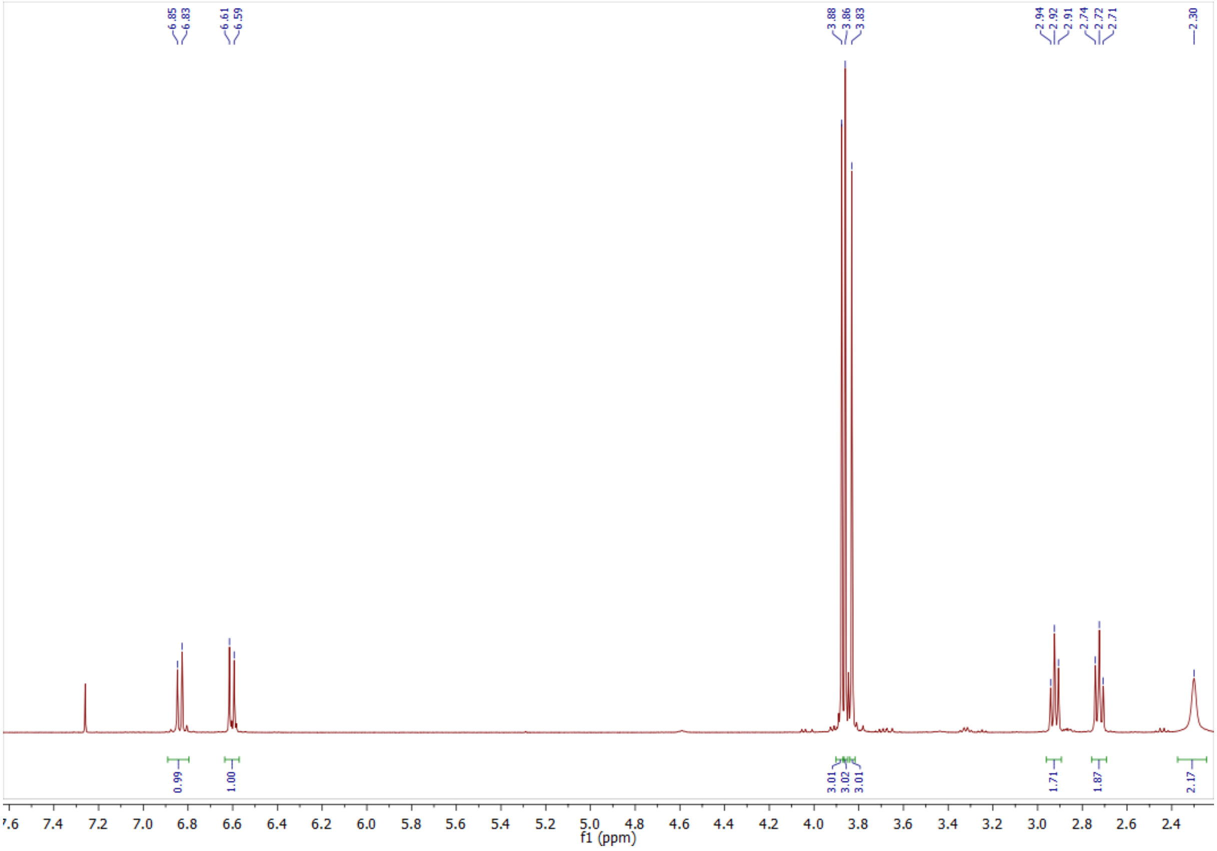

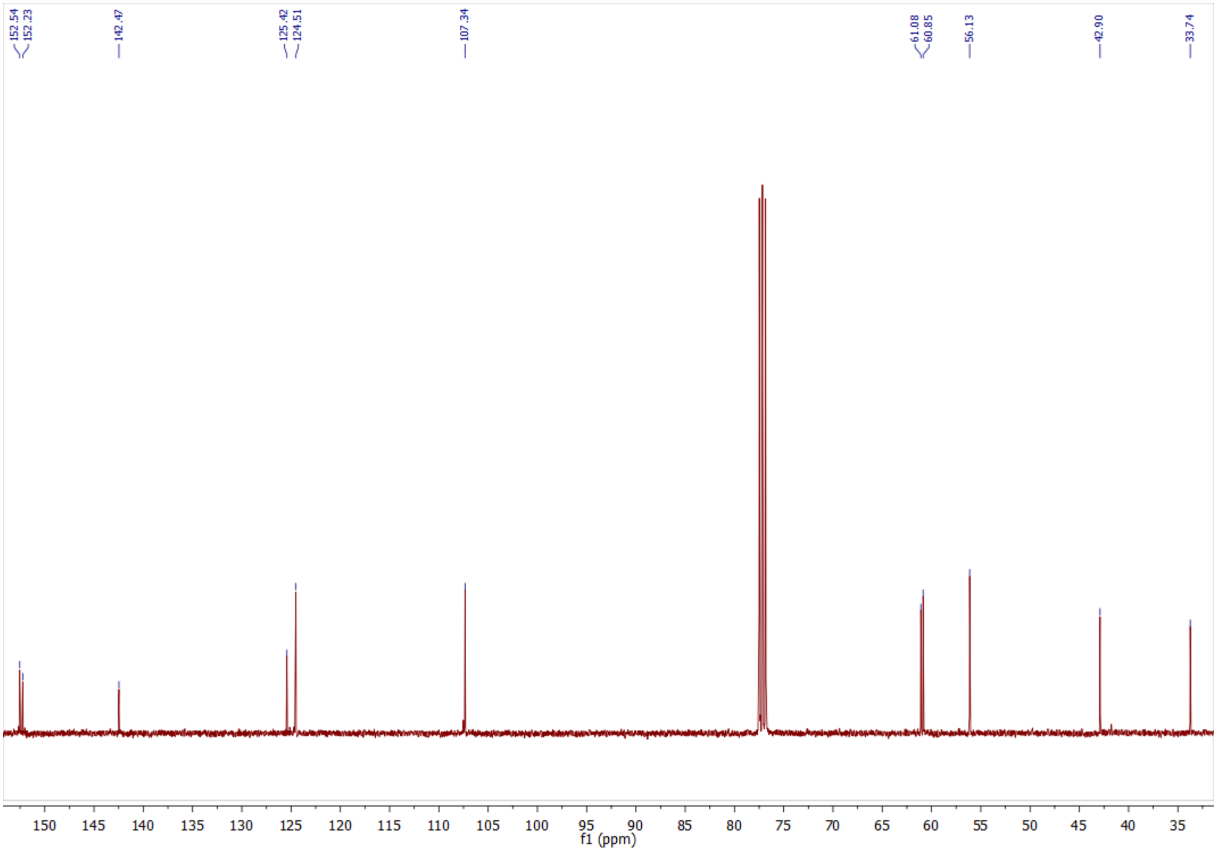

Ethyl (2-(benzo[*d*][1,3]dioxol-5-yl)ethyl) carbamate (**A2**)

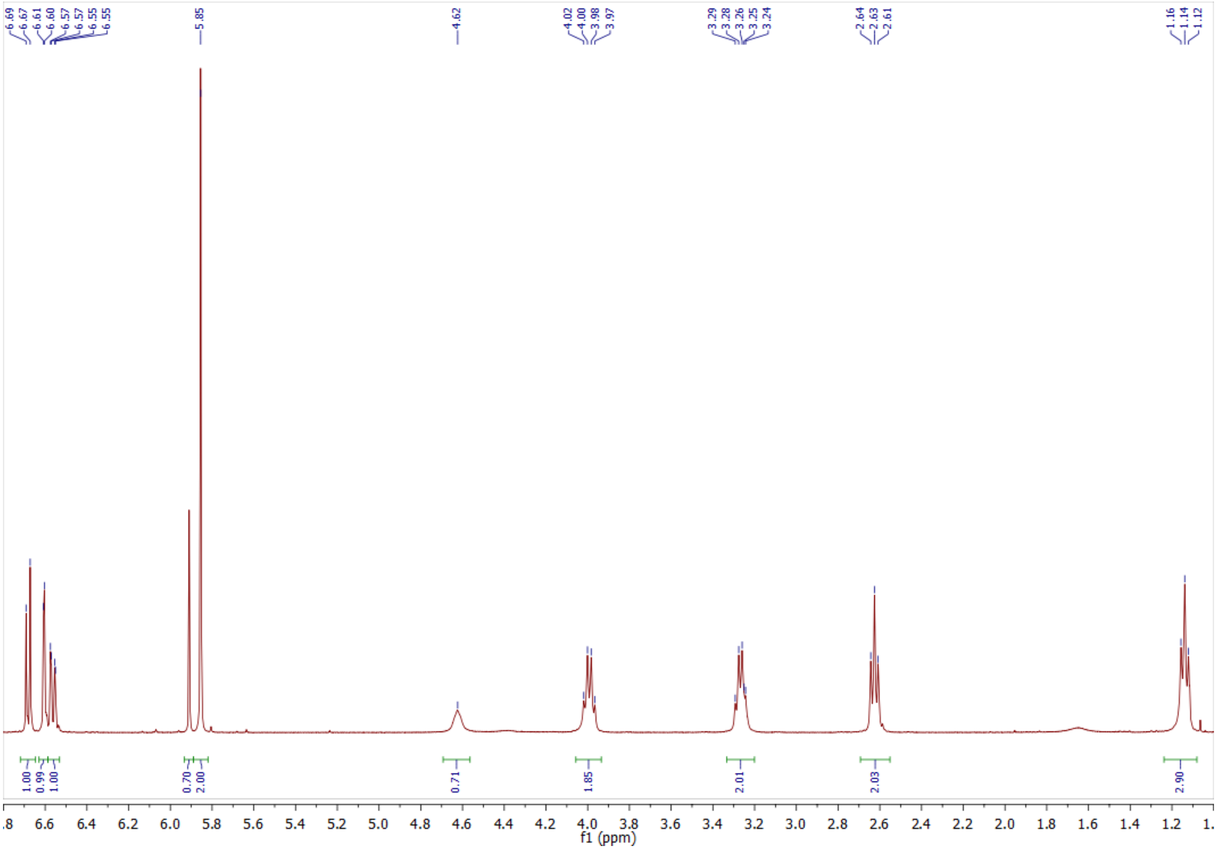

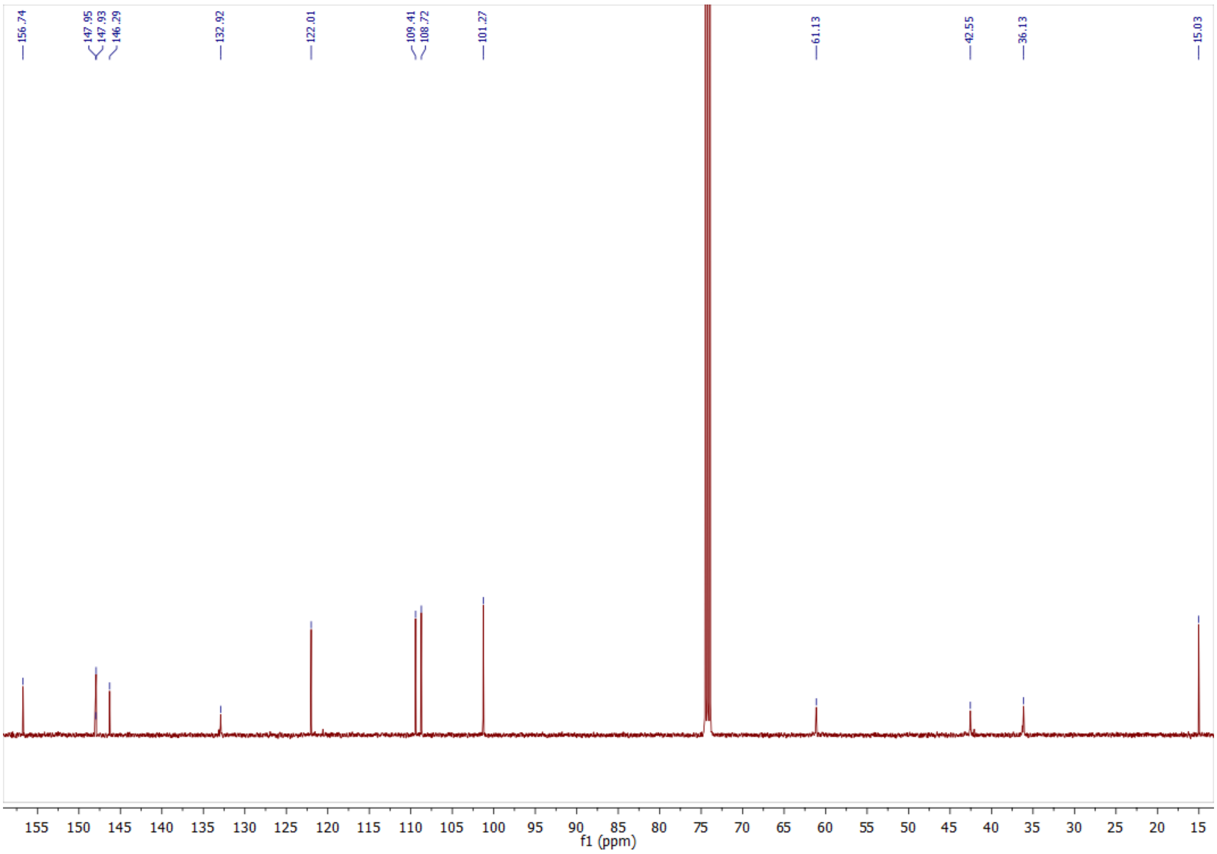

Ethyl (2,3,4-trimethoxyphenethyl) carbamate (**A4**)

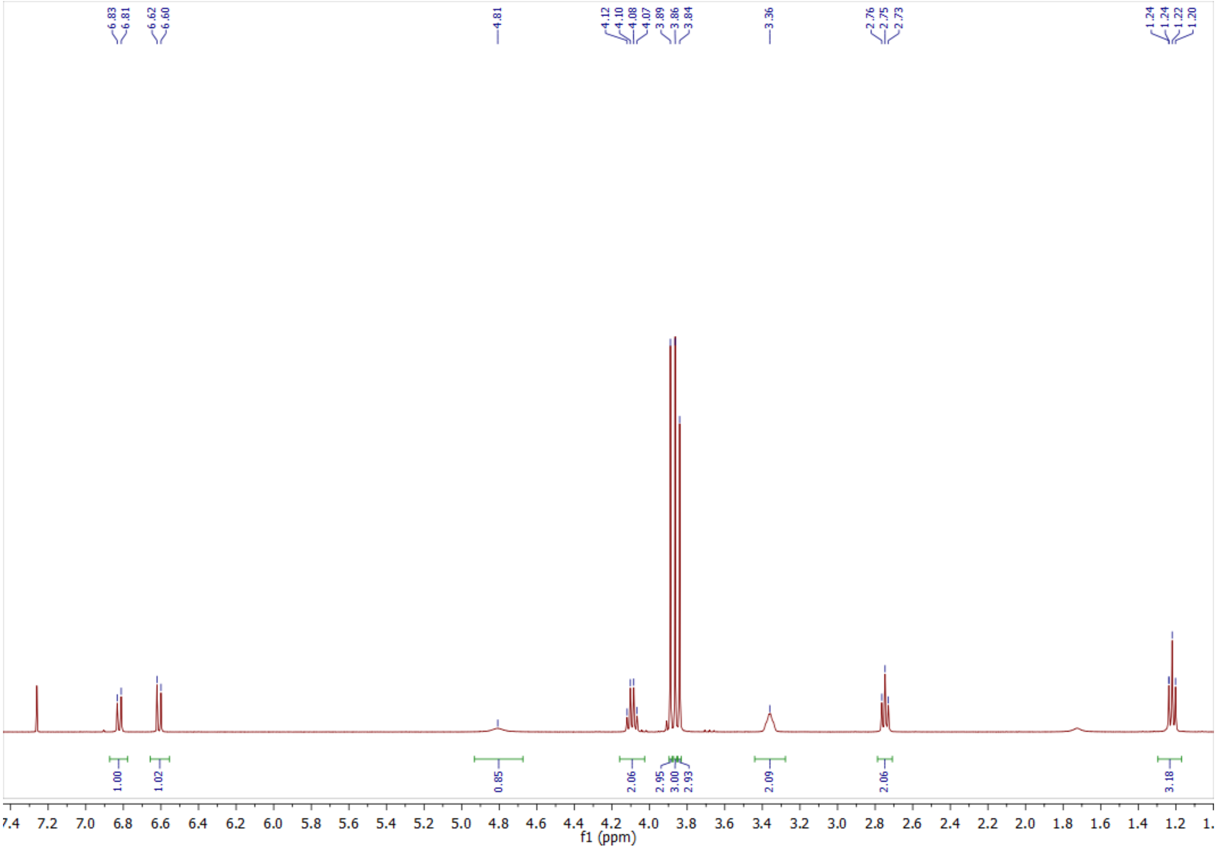

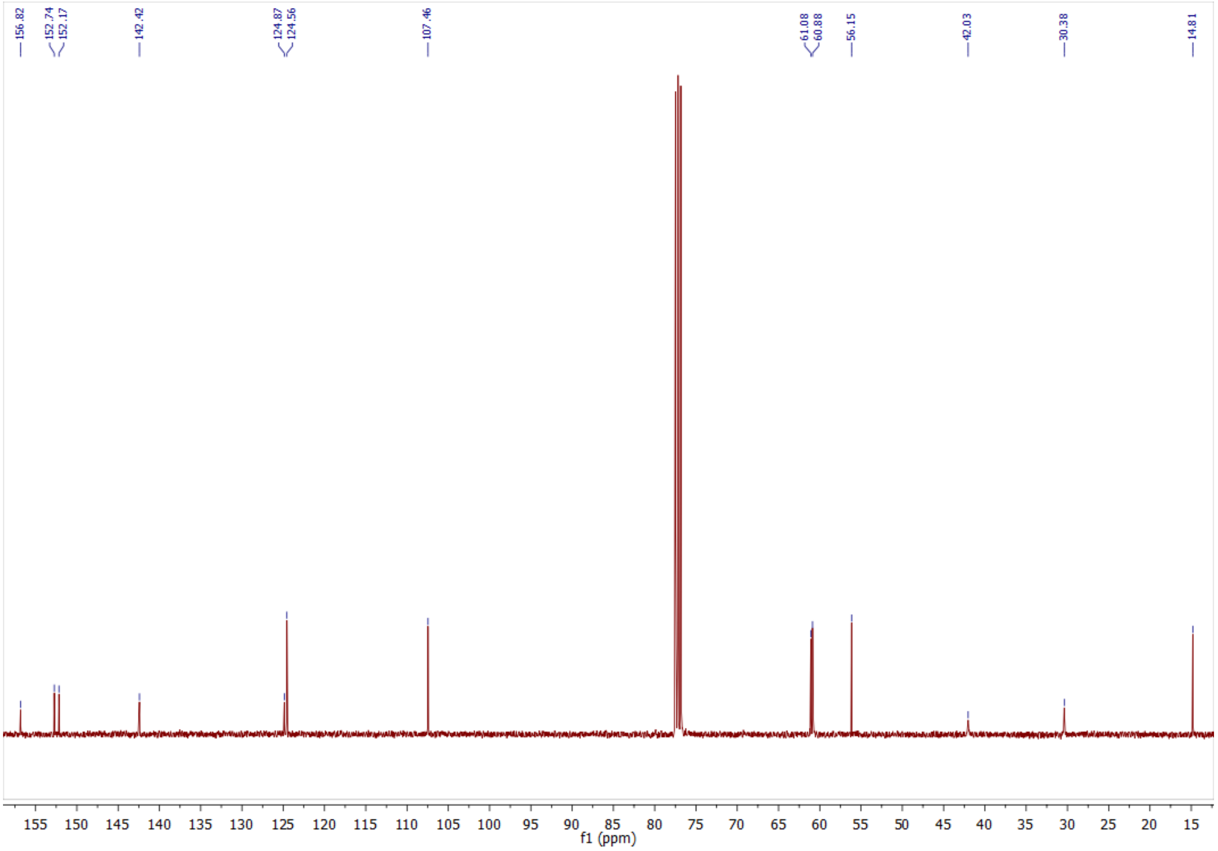

(±)-Ethyl 1-(3-((ethoxycarbonyl)oxy)-4-methoxybenzyl)-6,7-dimethoxy-3,4-dihydroisoquinoline-2(1*H*)-carboxylate (**5b**)

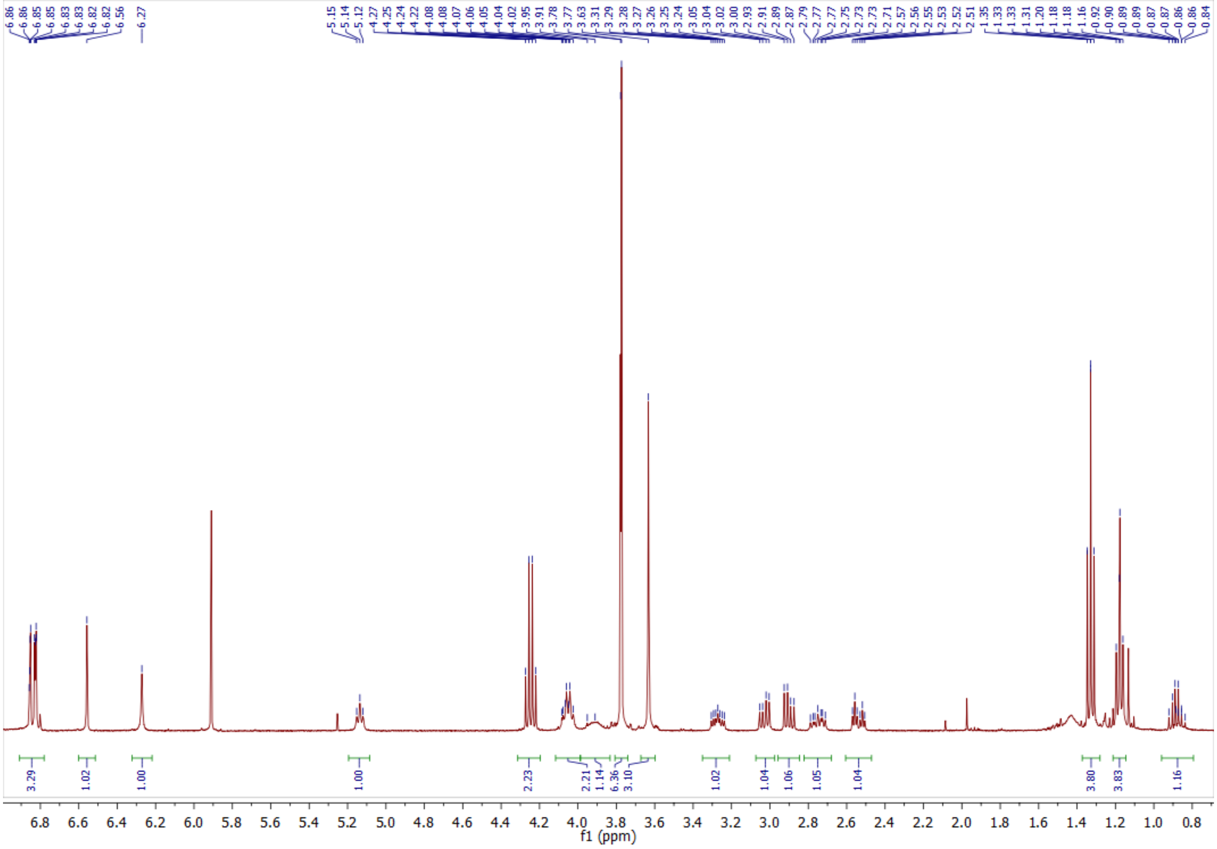

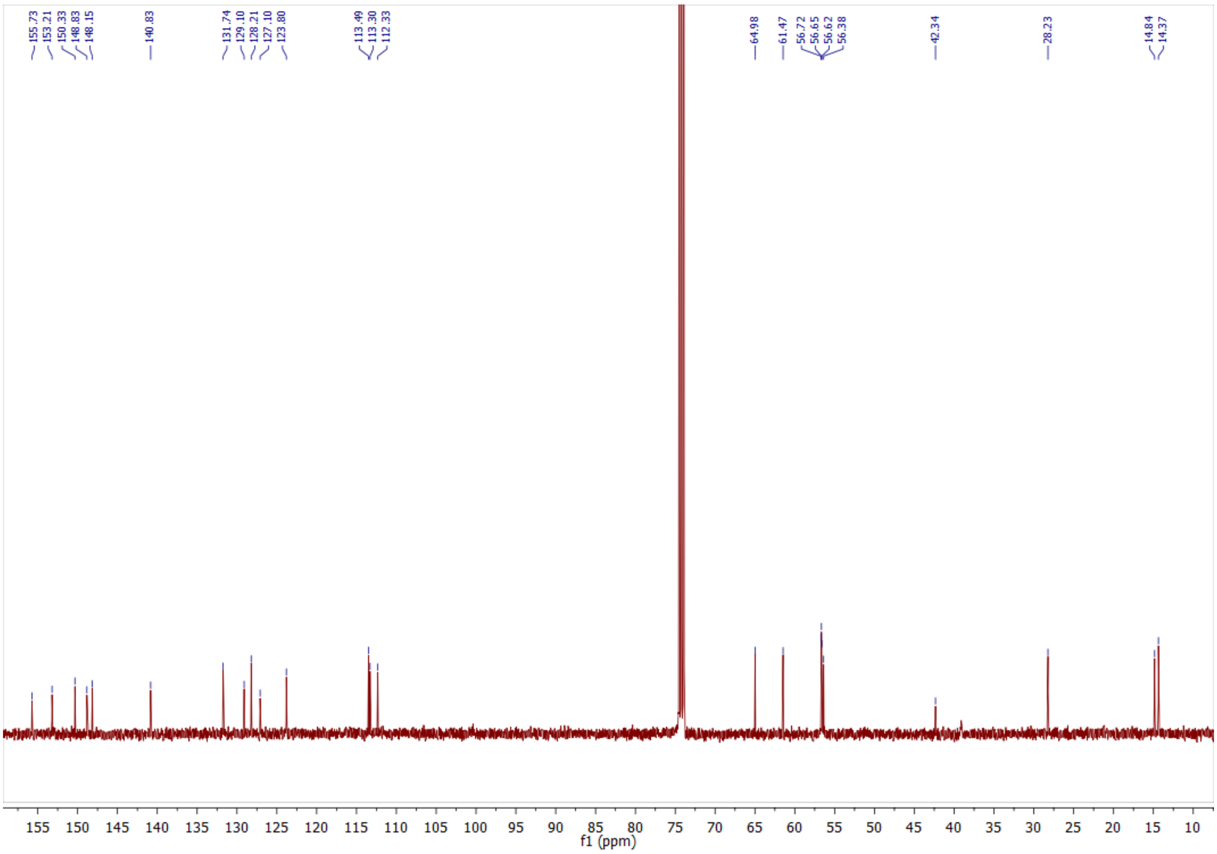

(±)-Ethyl 1-(4-((ethoxycarbonyl)oxy)-3-methoxybenzyl)-6,7-dimethoxy-3,4-dihydroisoquinoline-2(1*H*)-carboxylate (**5c**)

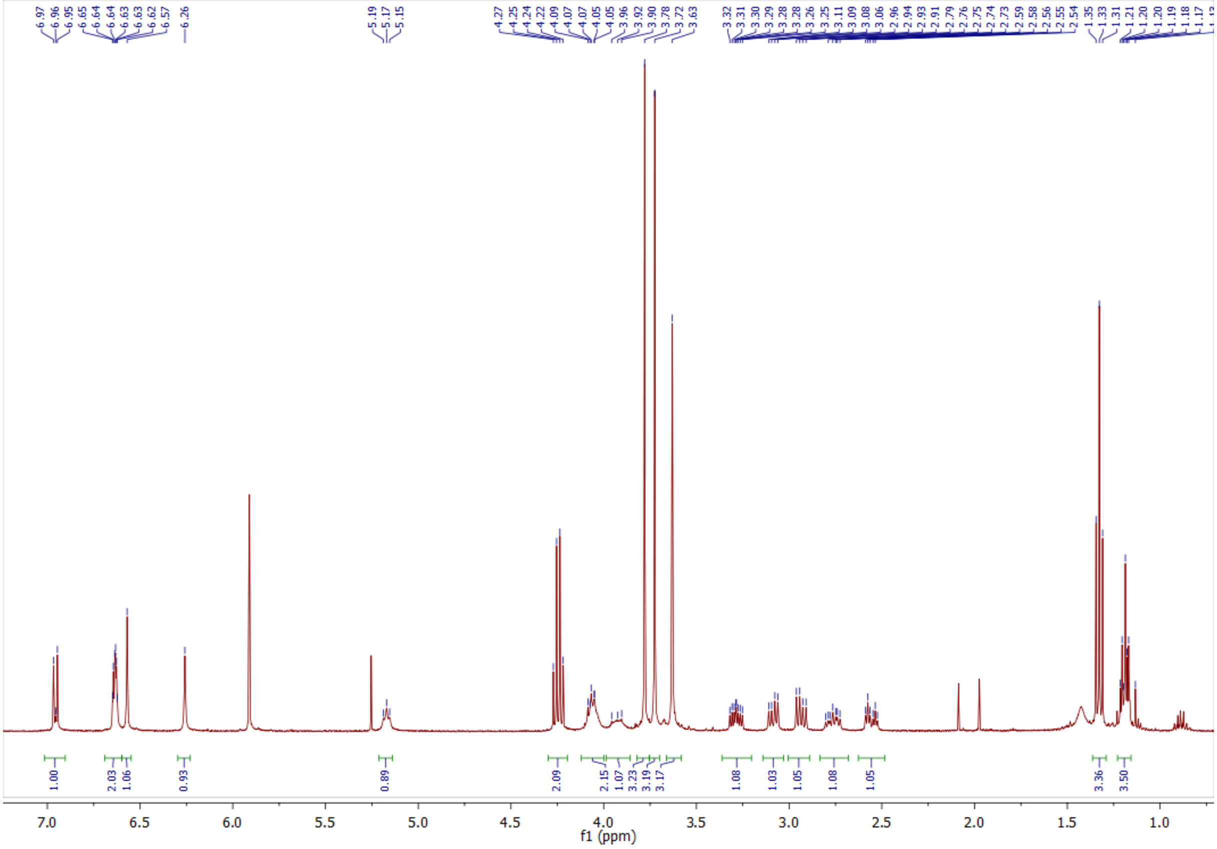

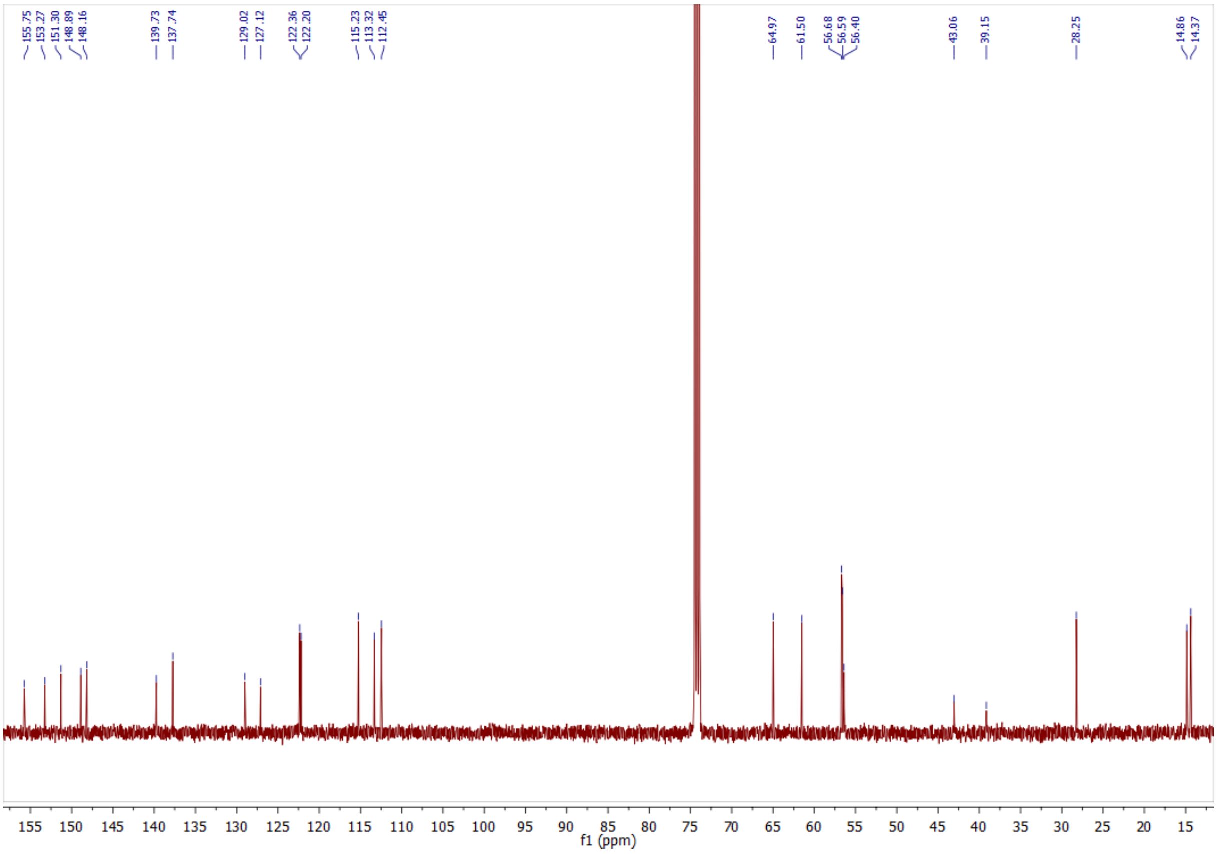

(±)-Ethyl 1-(3-((ethoxycarbonyl)oxy)-4-methoxybenzyl)-7-hydroxy-6-methoxy-3,4-dihydroisoquinoline-2(1*H*)-carboxylate (**5d**)

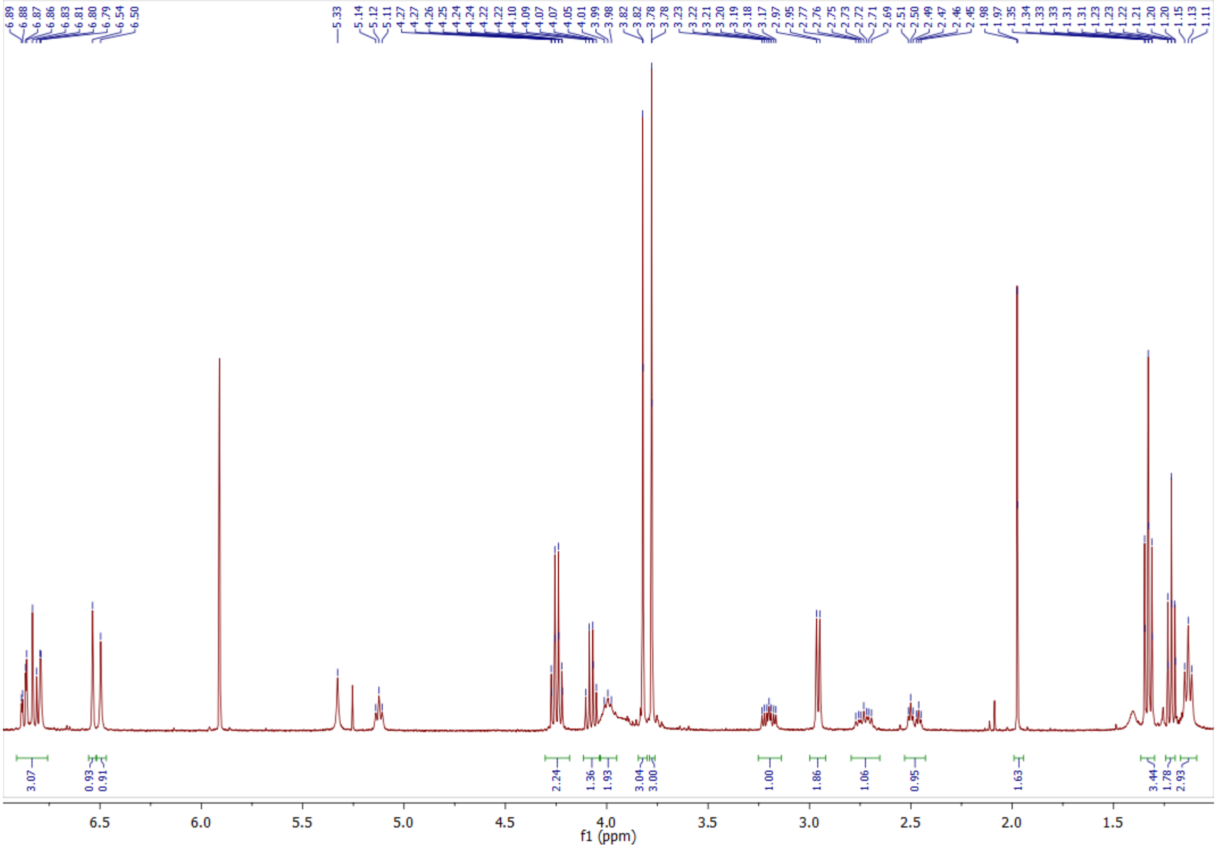

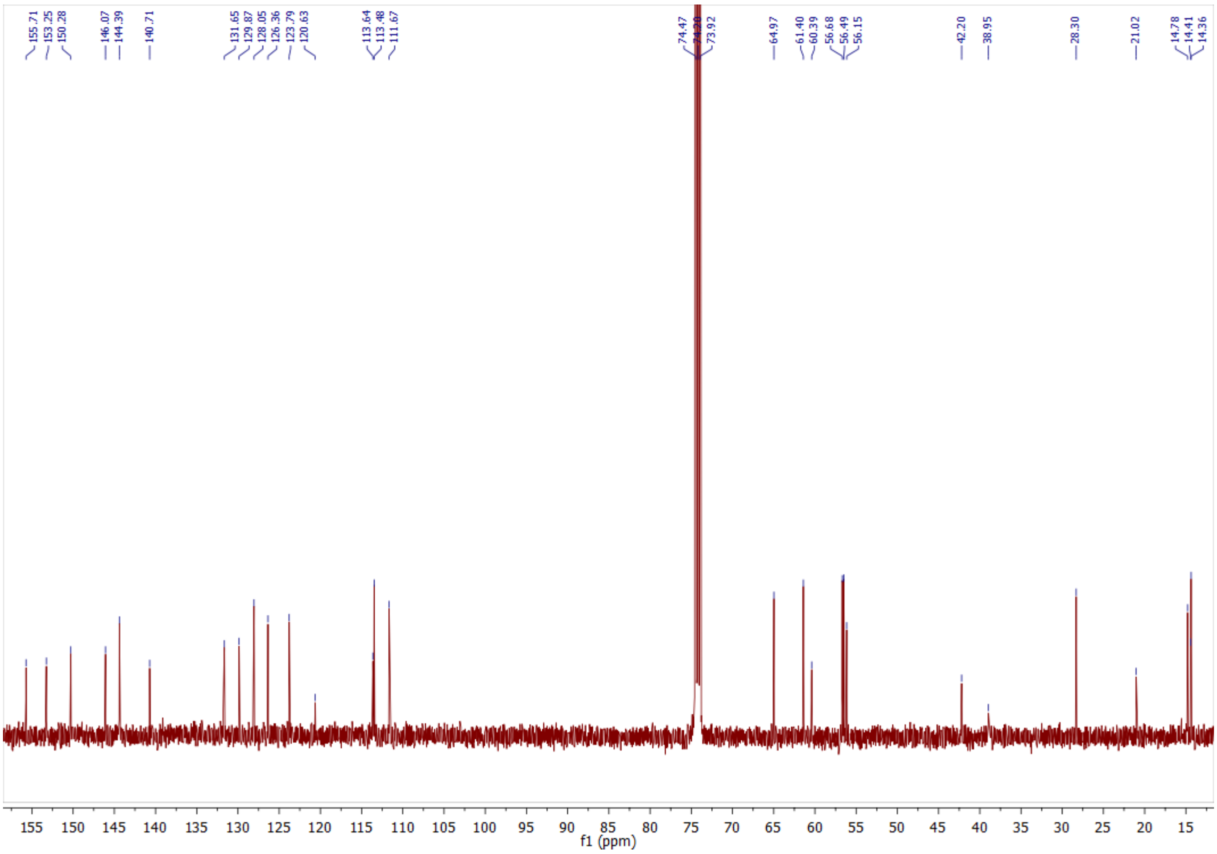

(±)-Ethyl 1-(4-((ethoxycarbonyl)oxy)-3-methoxybenzyl)-7-hydroxy-6-methoxy-3,4-dihydro-isoquinoline-2(1*H*)-carboxylate (**5e**)

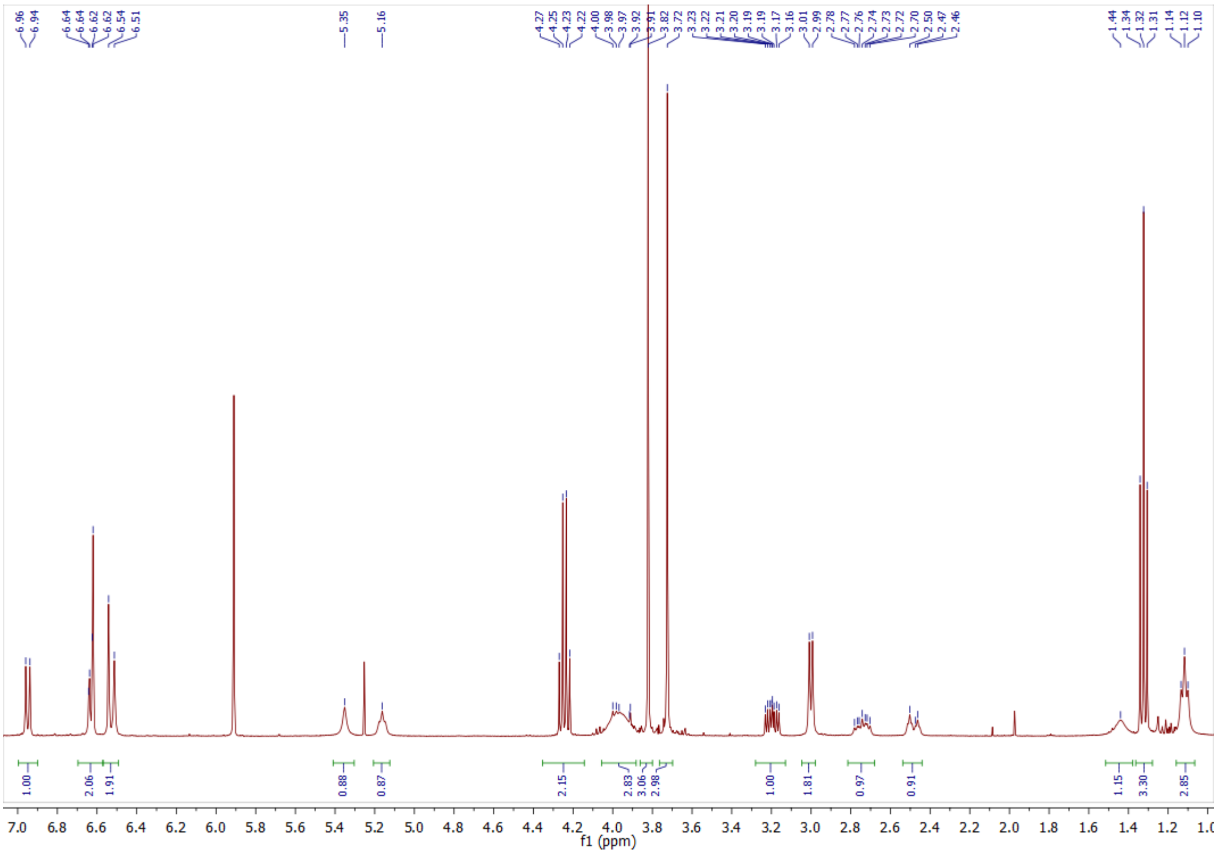

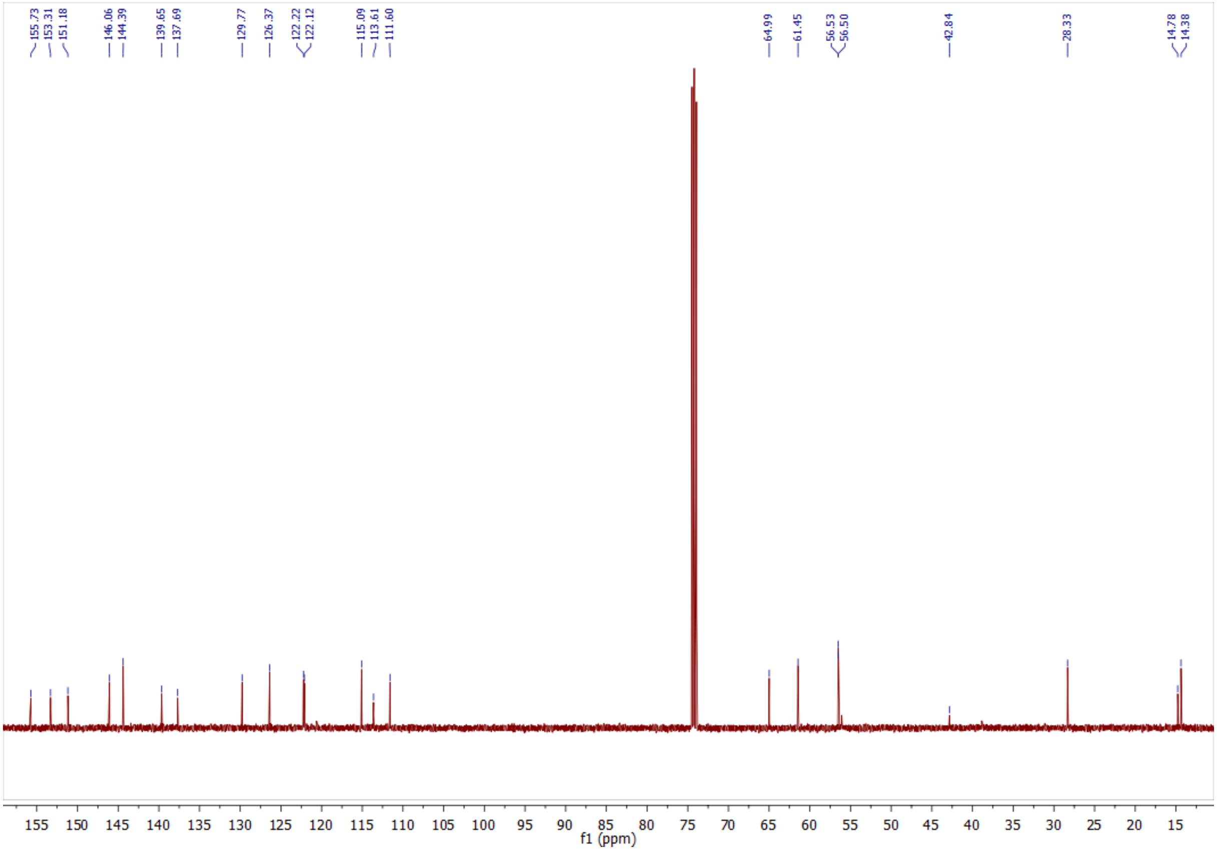

(±)-Ethyl (*S*)-5-(4-((ethoxycarbonyl)oxy)benzyl)-7,8-dihydro-[1,3]dioxolo[4,5-g]isoquinoline-6(5*H*)-carboxylate (**5f**)

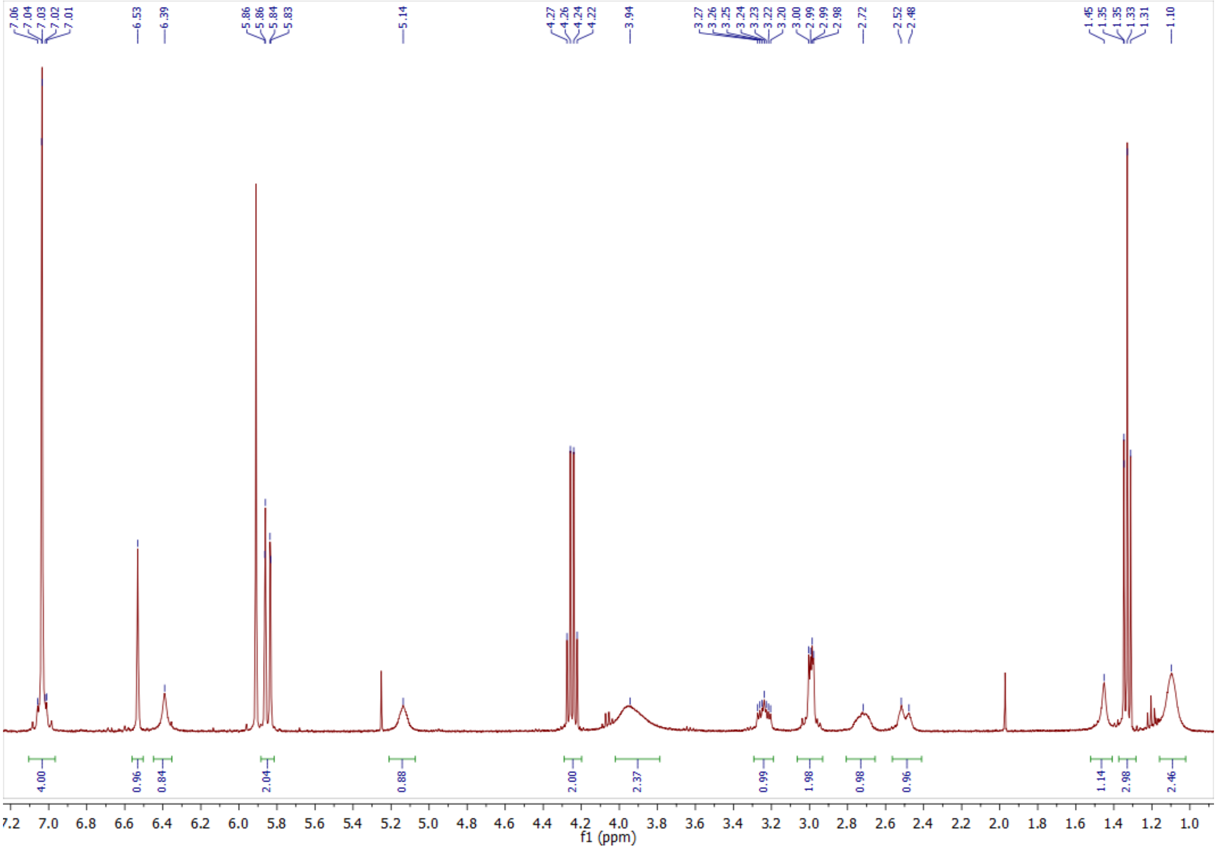

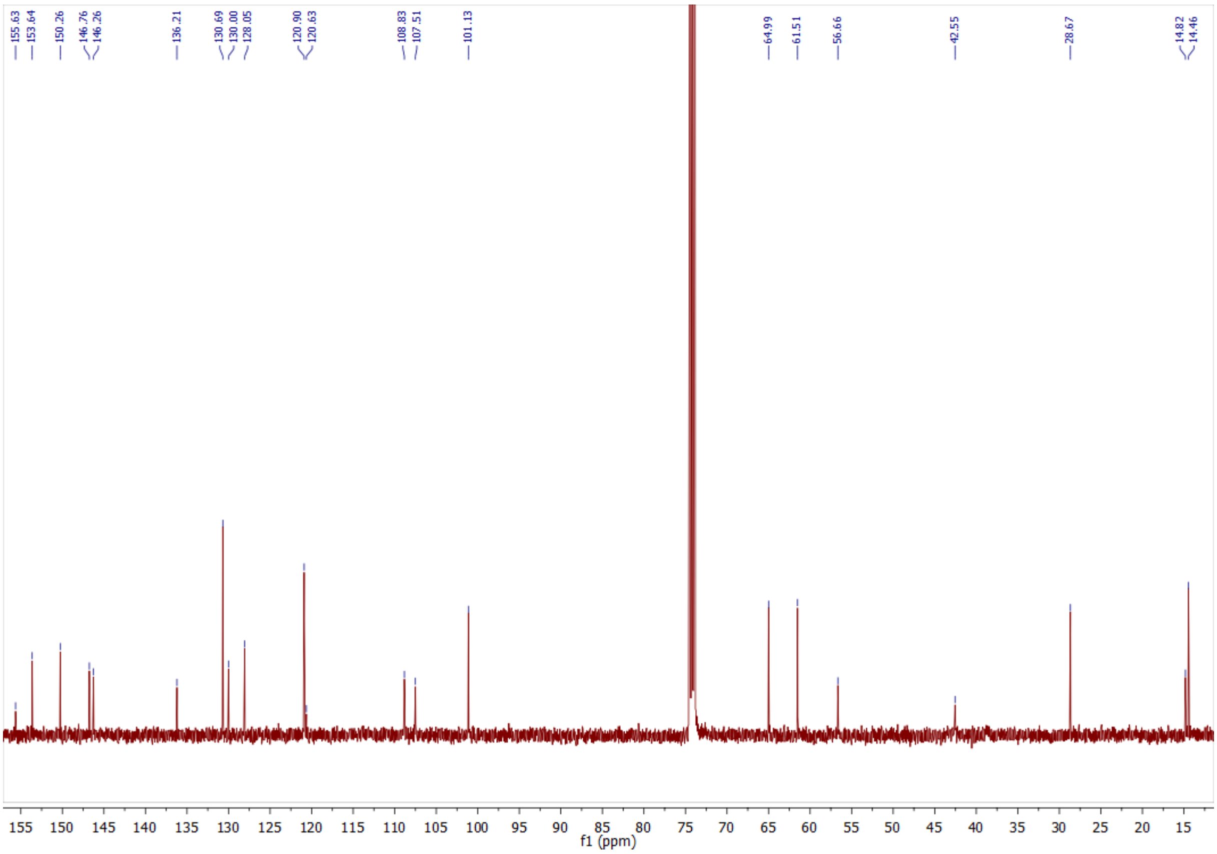

(±)-Ethyl 5-(3-((ethoxycarbonyl)oxy)-4-methoxybenzyl)-7,8-dihydro-[1,3]dioxolo[4,5-g]isoquinoline-6(5*H*)-carboxylate (**5g**)

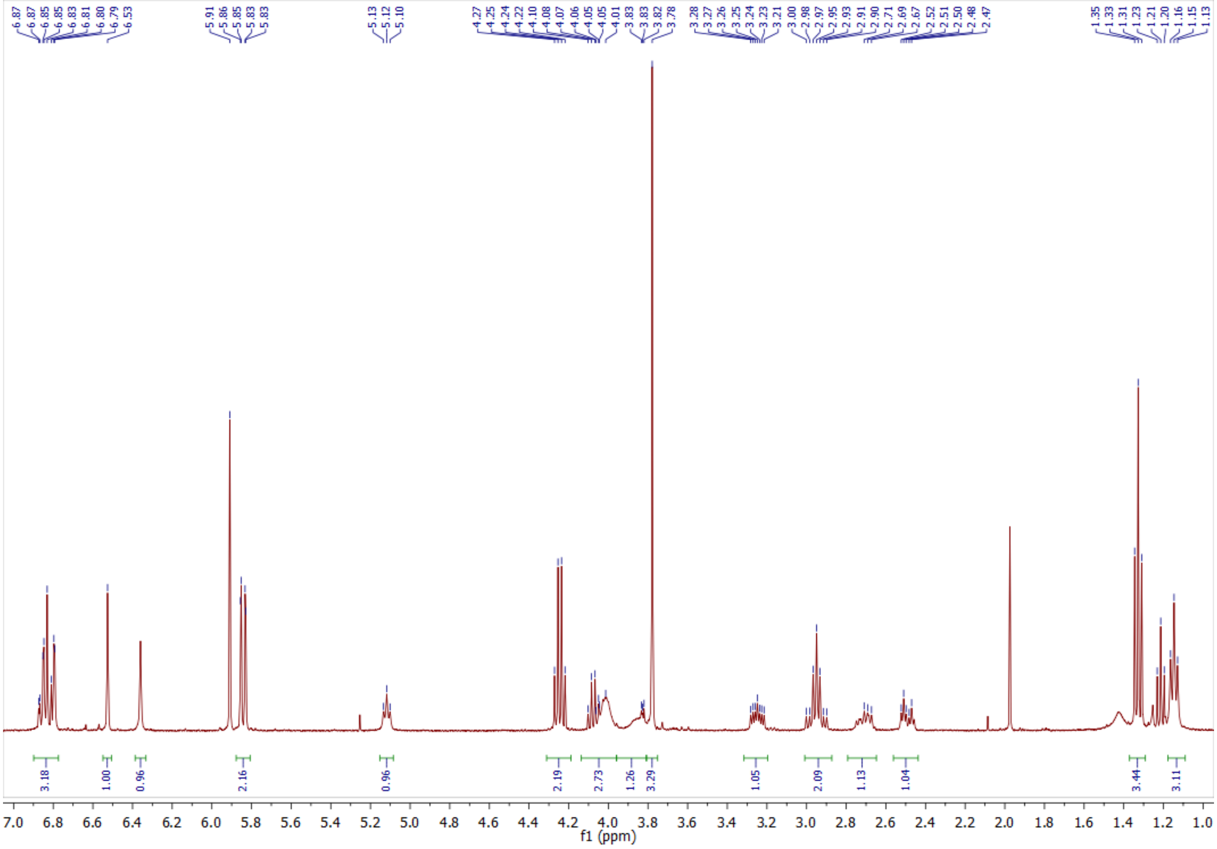

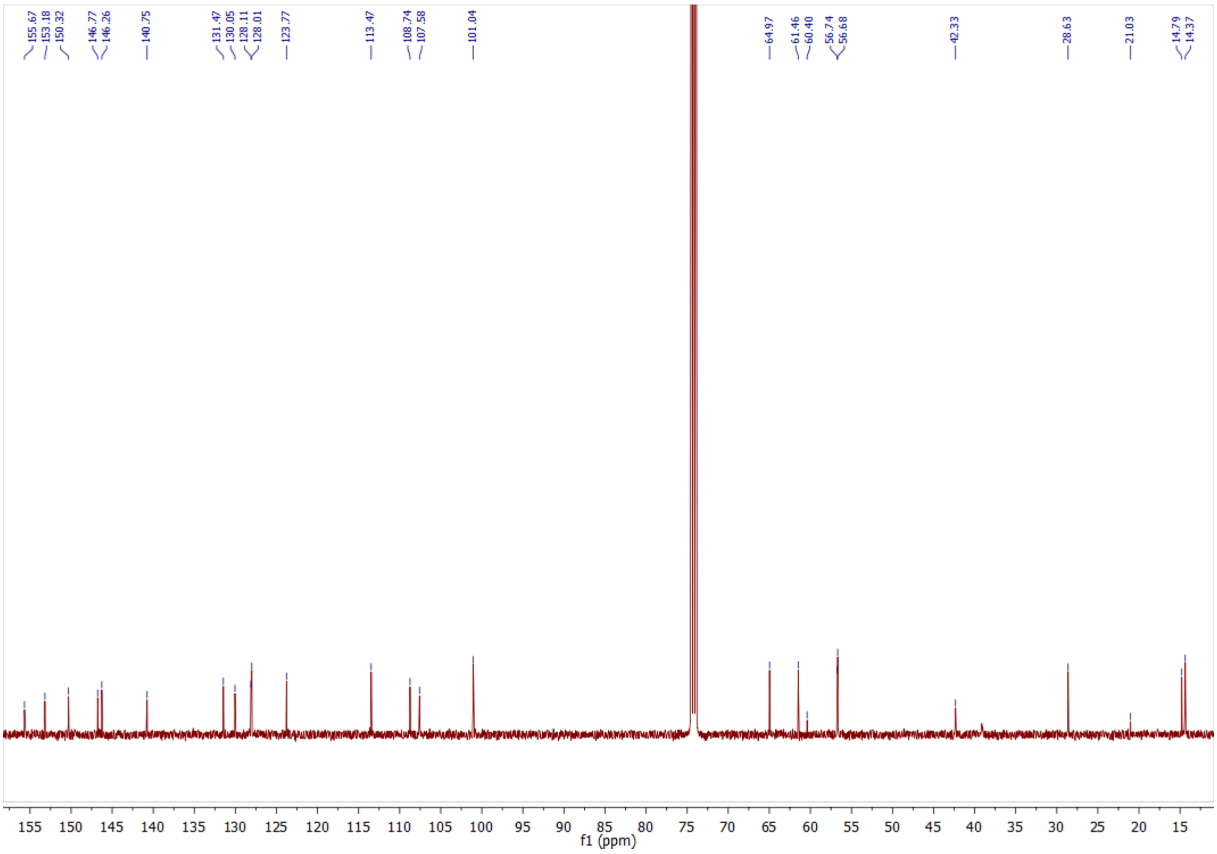

(±)-Ethyl 1-(4-((ethoxycarbonyl)oxy)benzyl)-5,6,7-trimethoxy-3,4-dihydroisoquinoline-2(1H)-carboxylate (**5h**)

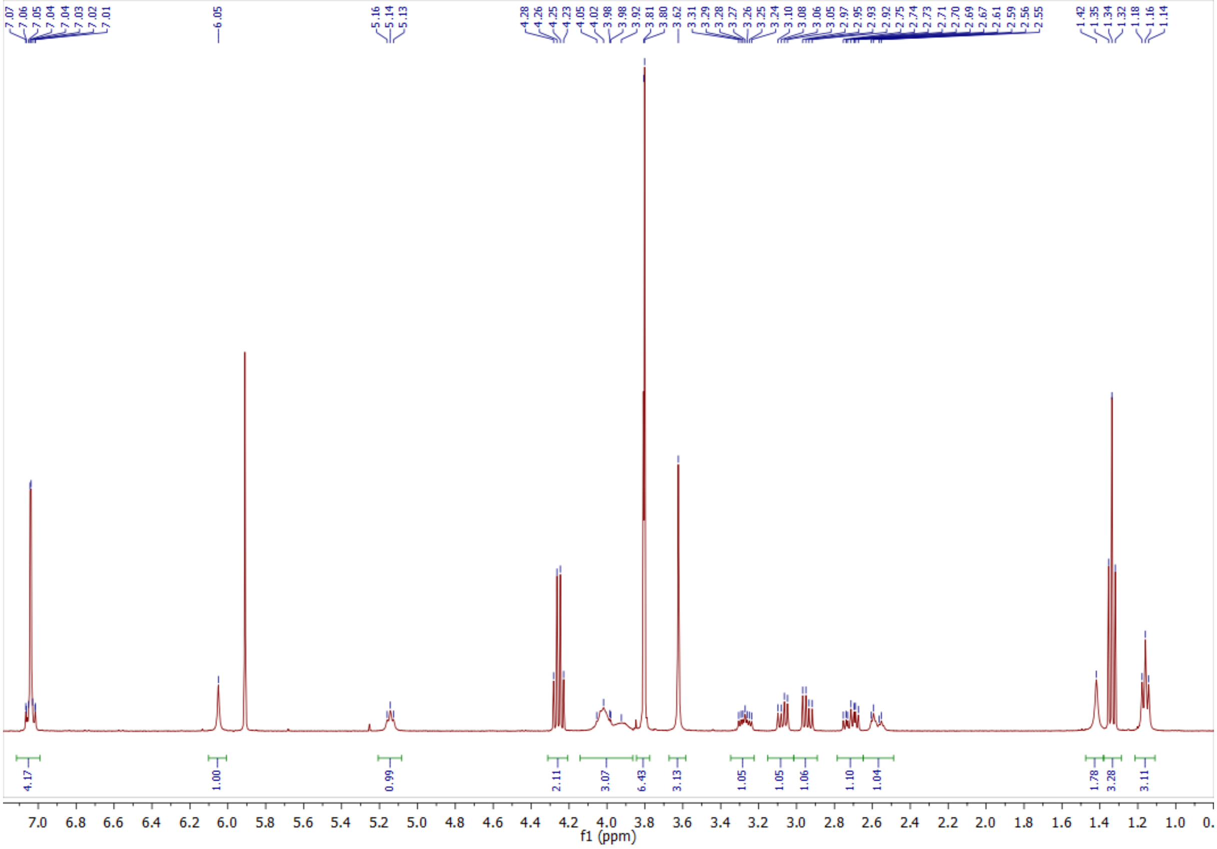

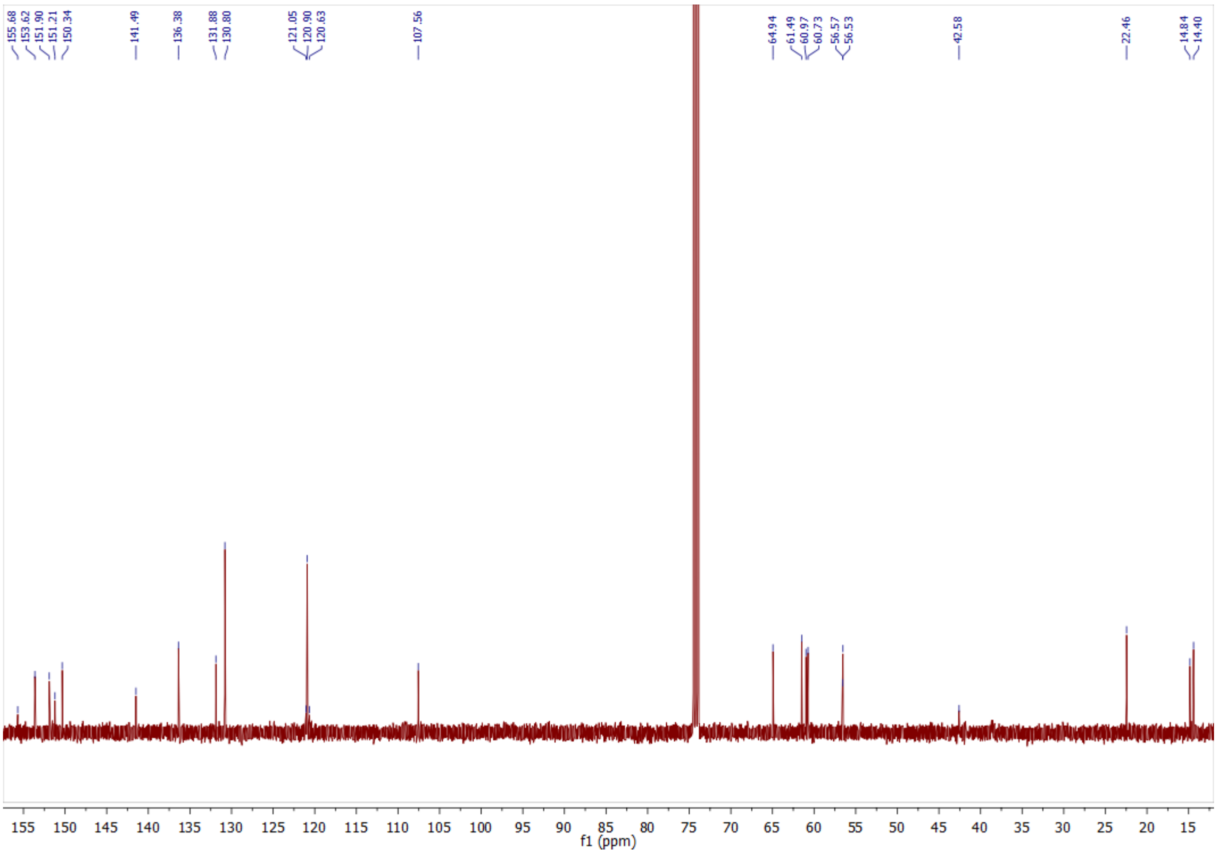

(±)-4-((6,7-Dimethoxy-2-methyl-1,2,3,4-tetrahydroisoquinolin-1-yl)methyl) phenol (*rac*-Armepavine) **(2a)**

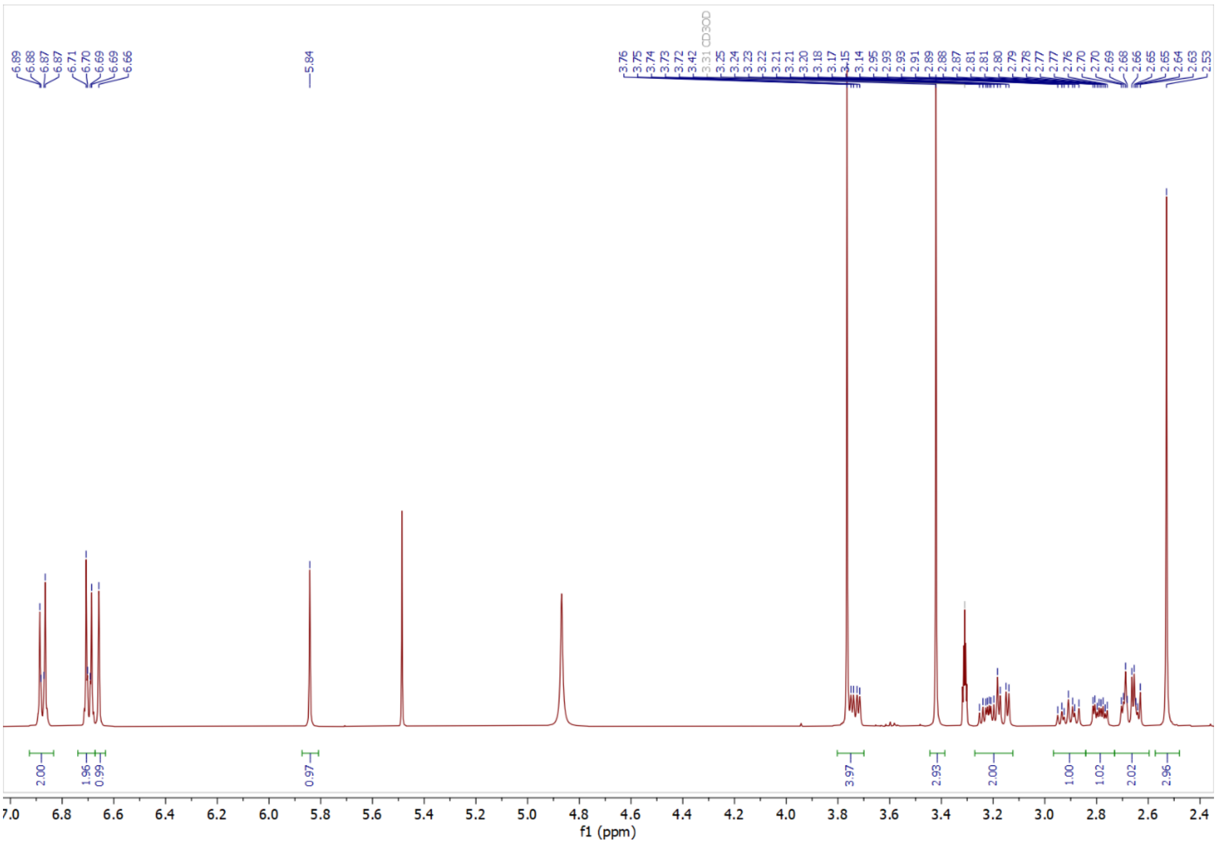

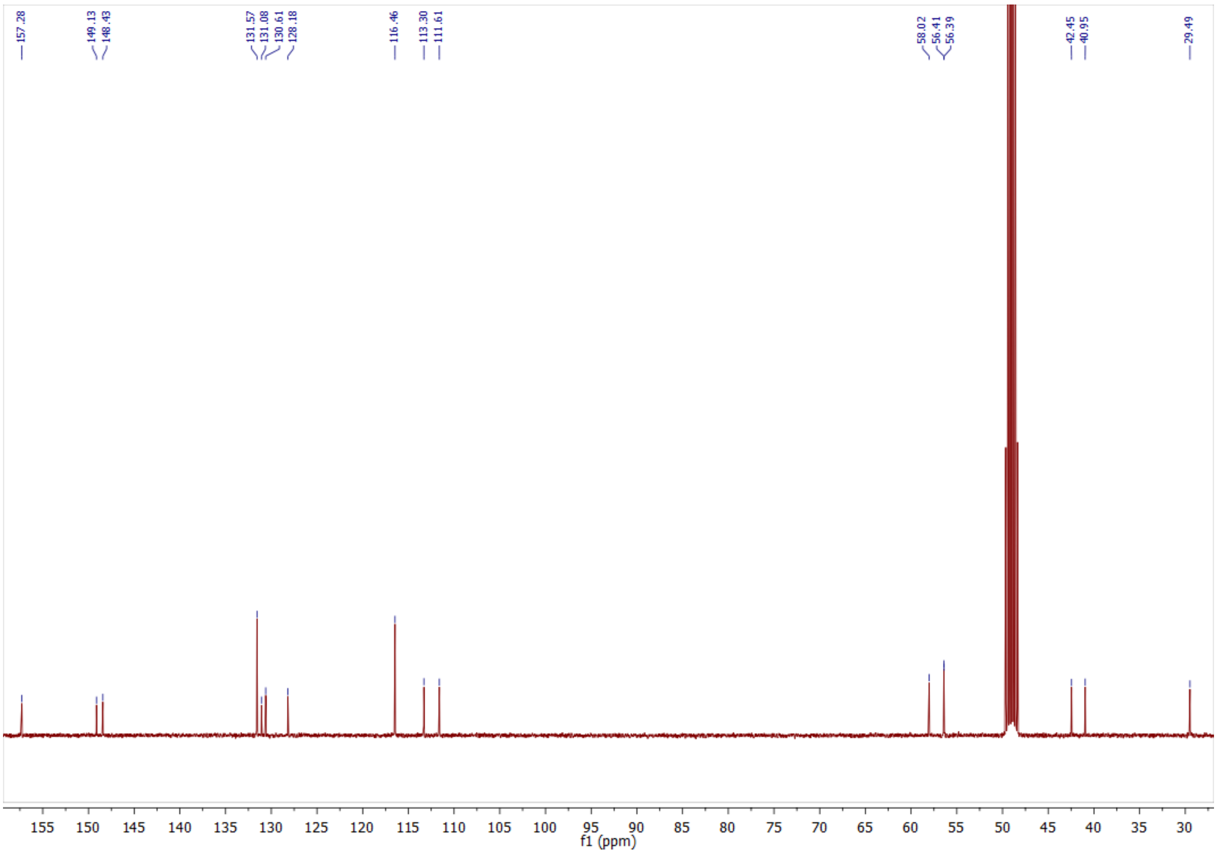

(±)-4-((6,7-Dimethoxy-1,2,3,4-tetrahydroisoquinolin-1-yl)methyl) phenol (*rac*-Norarmepavine) (**2b**)

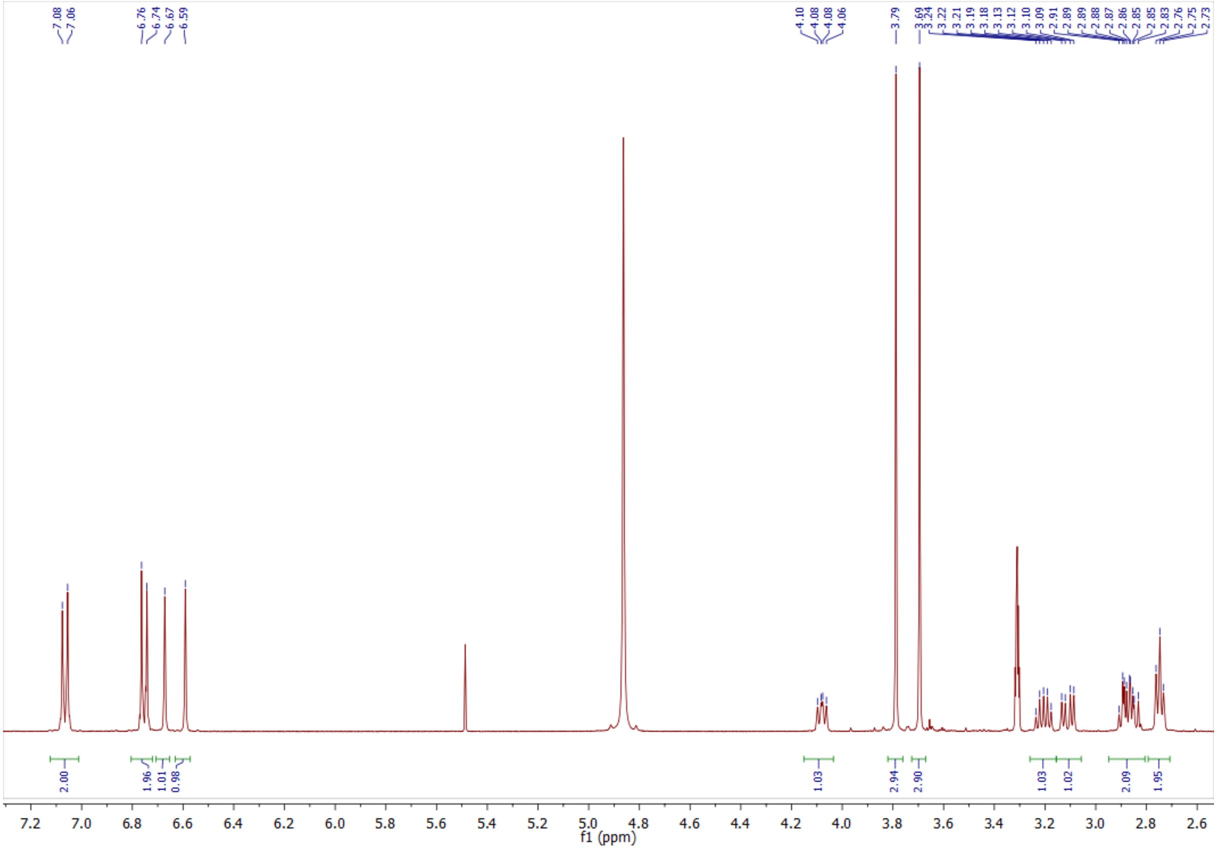

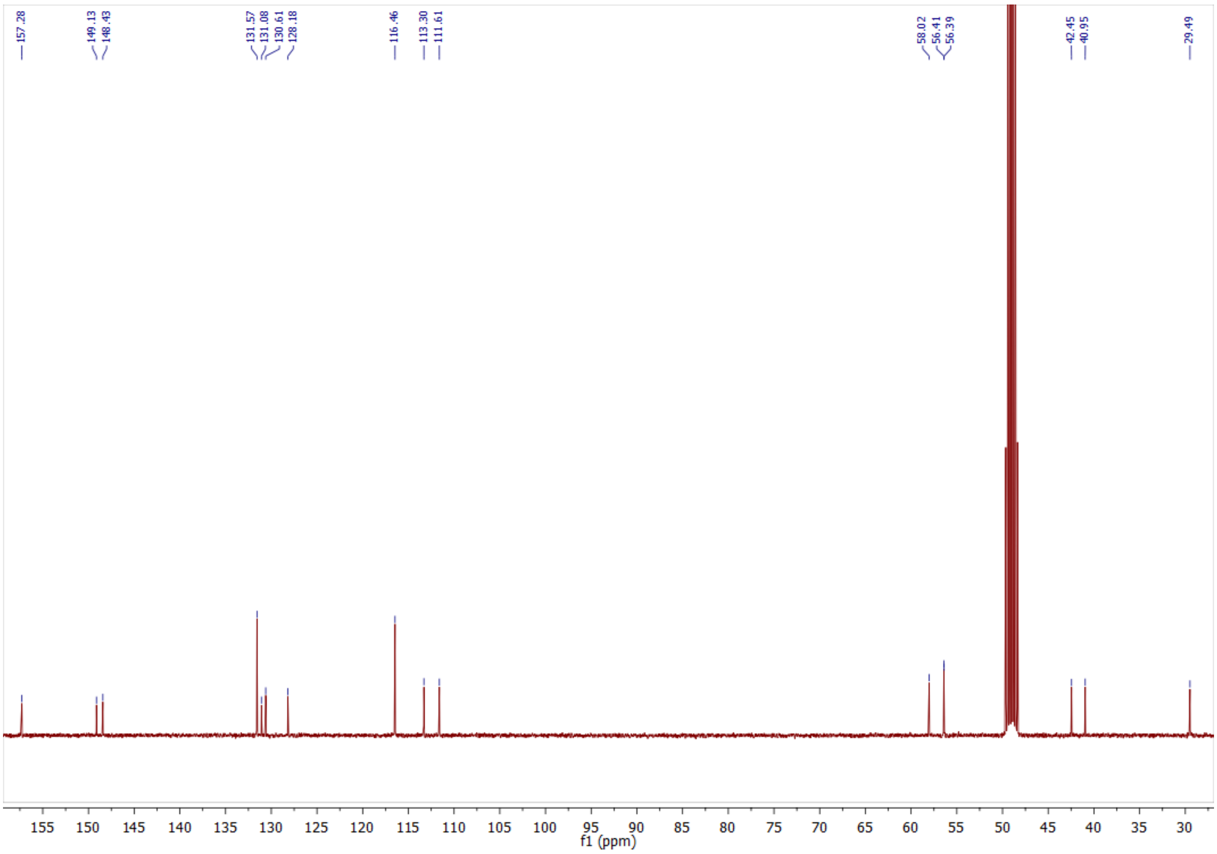

(±)-5-((6,7-Dimethoxy-2(1*H*)-methyl-1,2,3,4-tetrahydroisoquinolin-1-yl)methyl)-2-methoxyphenol (*rac*-Laudanine) (**2c**)

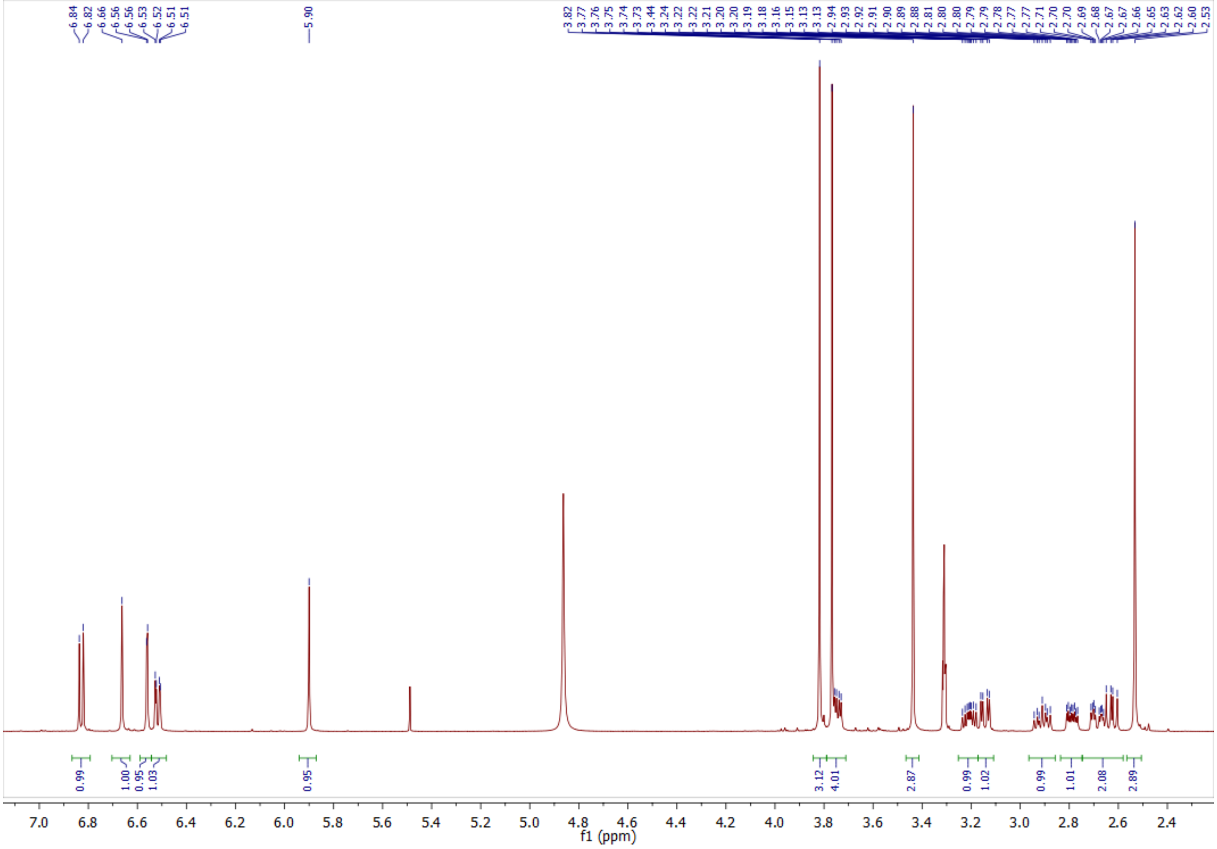

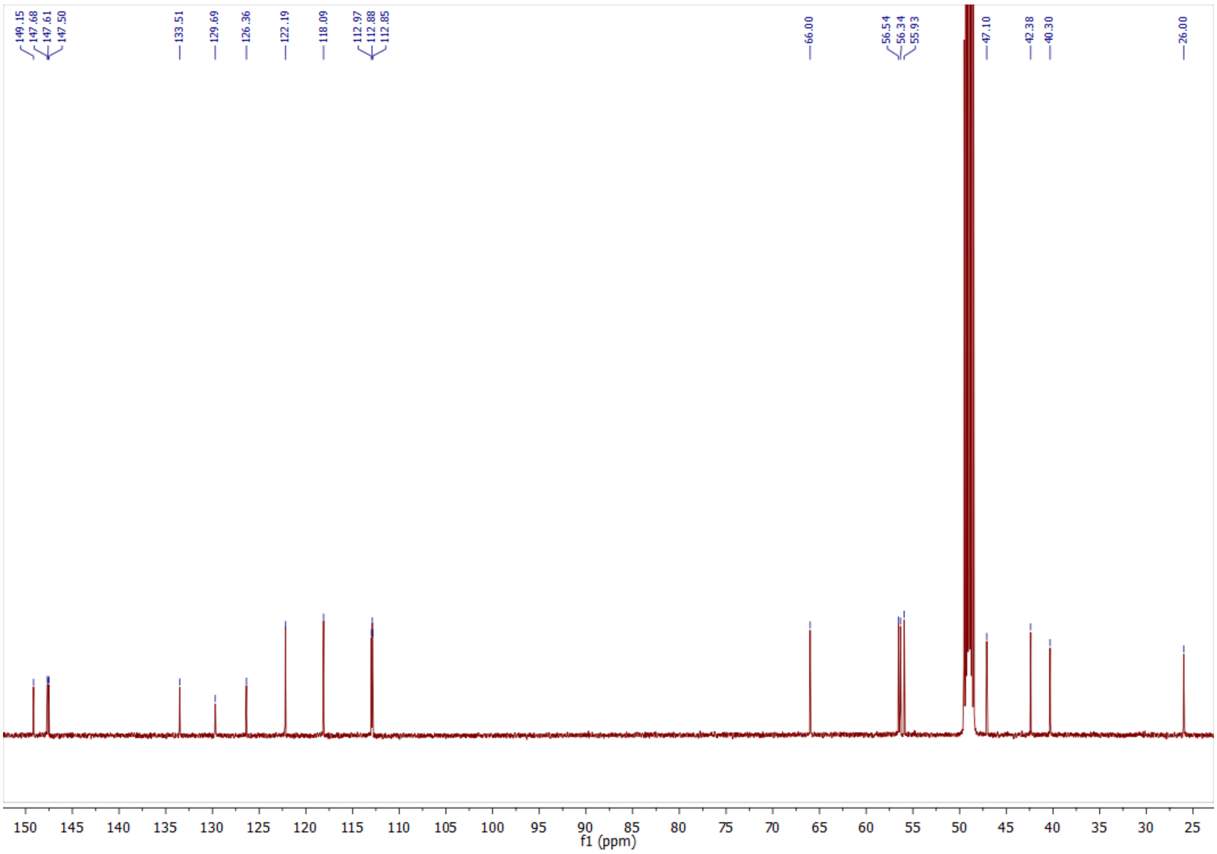

(±)-4-((6,7-Dimethoxy-2-methyl-1,2,3,4-tetrahydroisoquinolin-1-yl)methyl)-2-methoxyphenol (*rac*-Pseudocodamine) (**2d**)

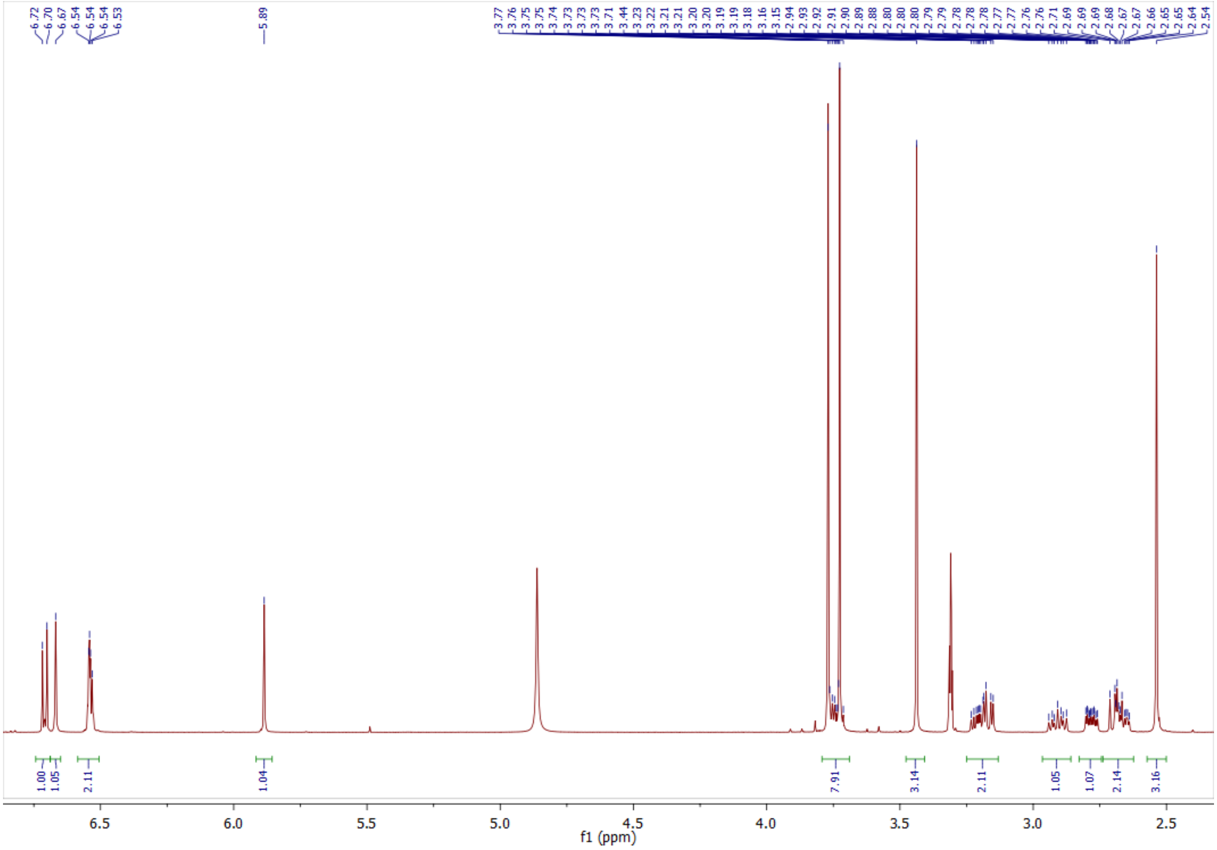

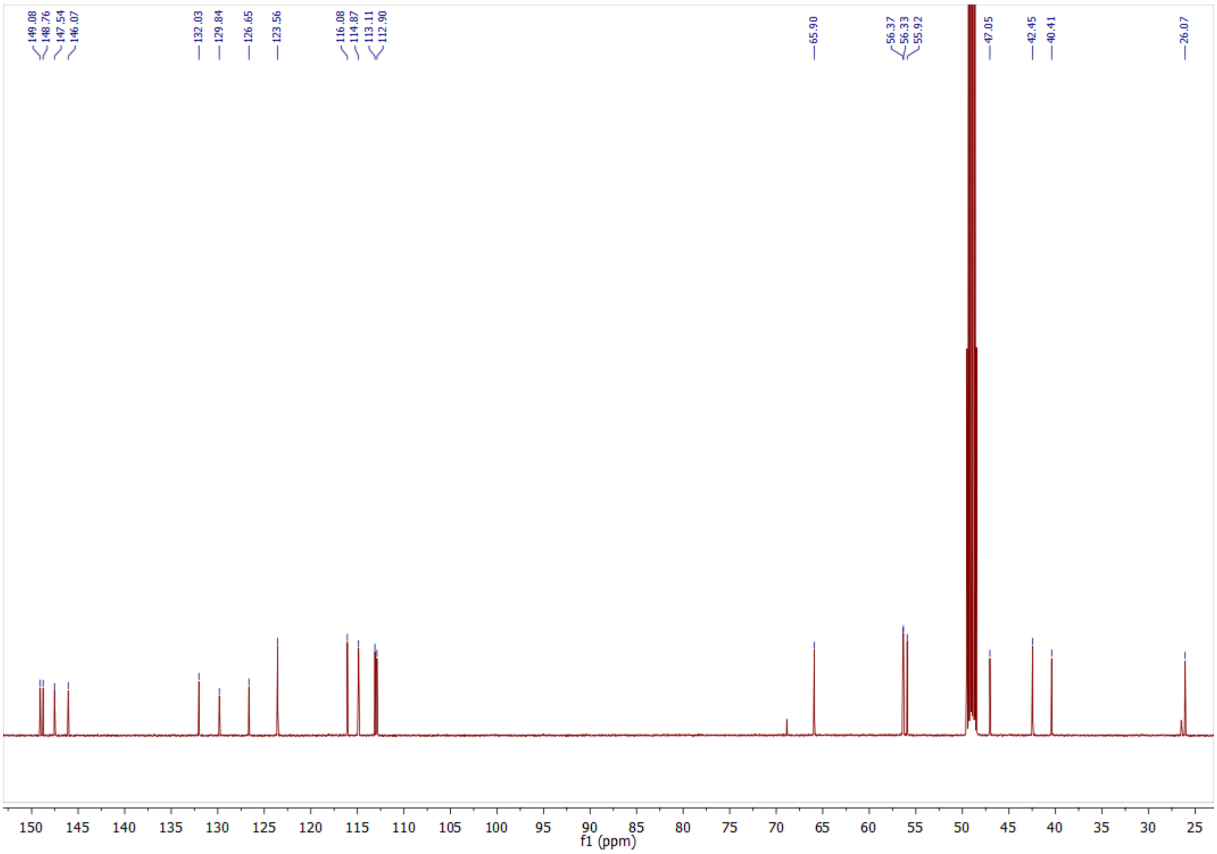

(±)-1-(3-Hydroxy-4-methoxybenzyl)-6-methoxy-2-methyl-1,2,3,4-tetrahydroisoquinolin-7-ol (*rac*-Reticuline) (**2e**)

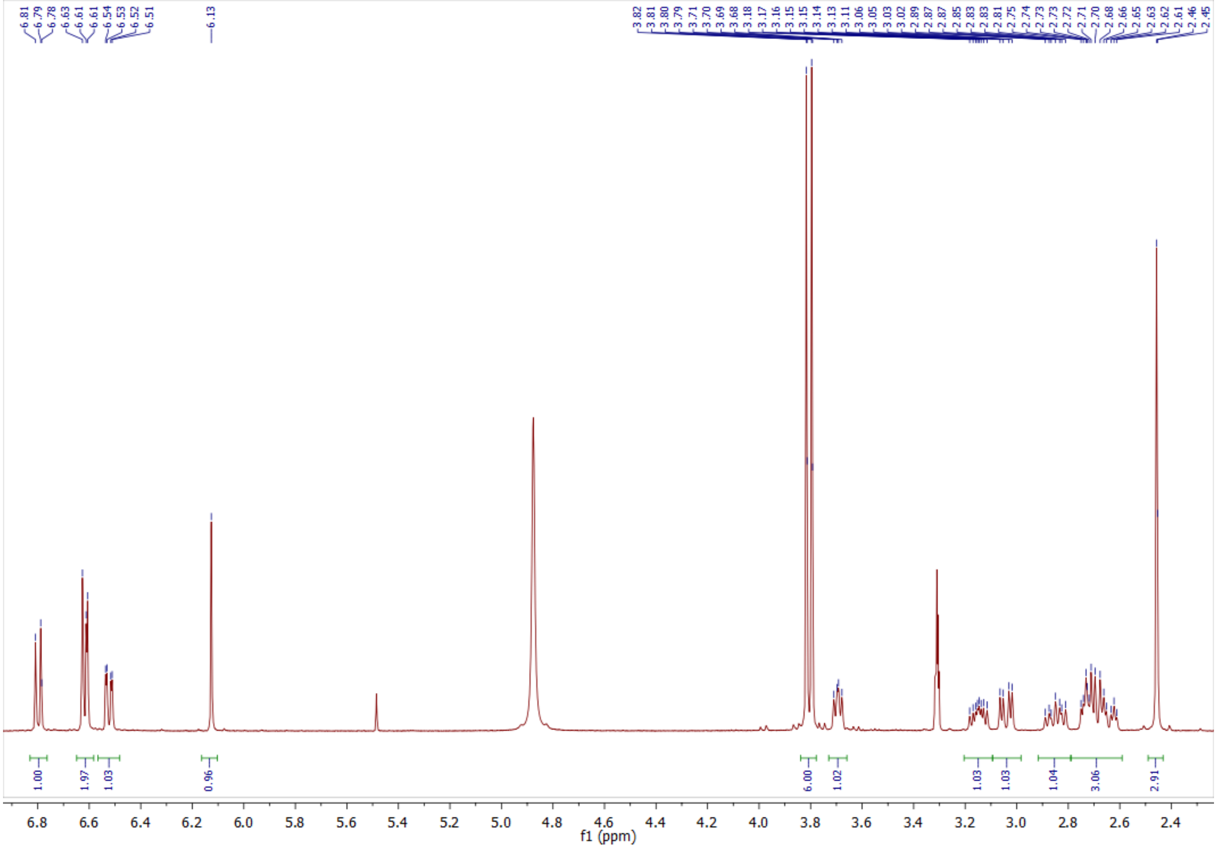

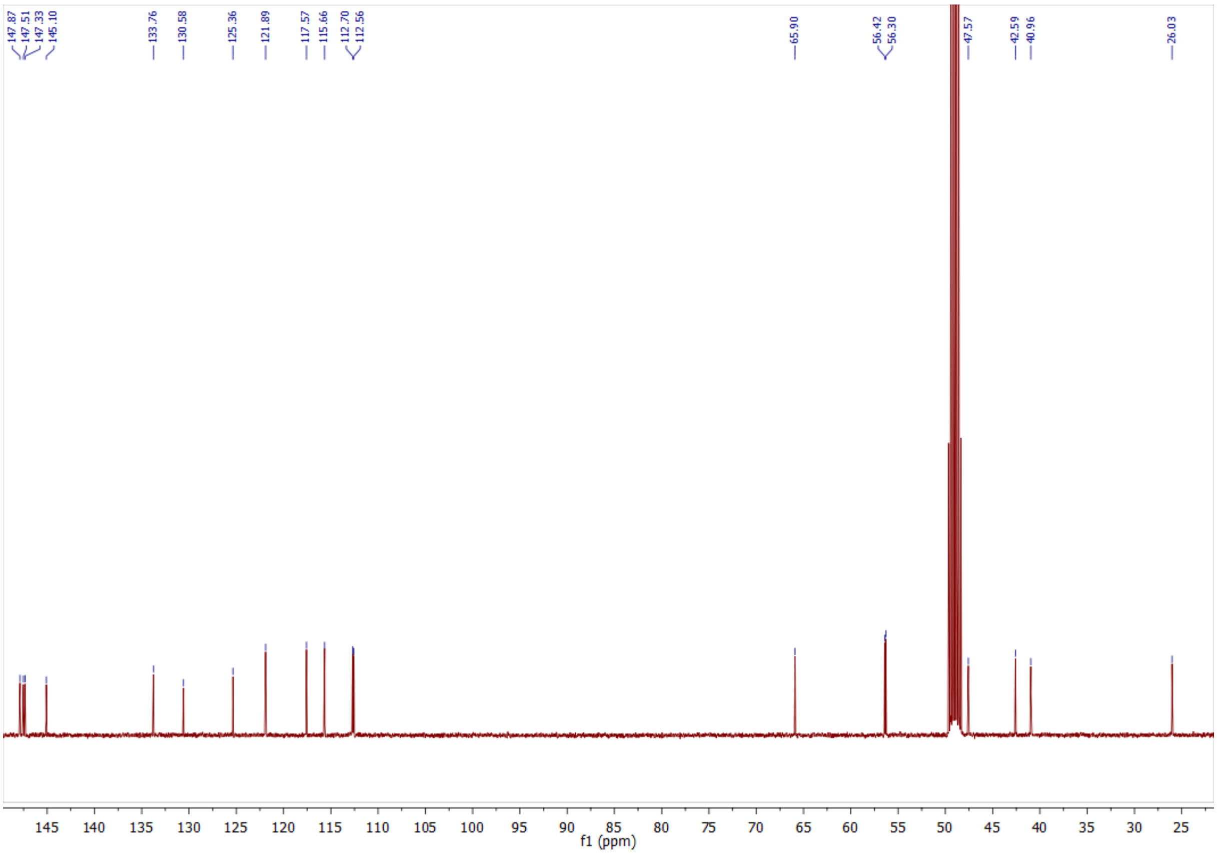

(±)-1-(4-Hydroxy-3-methoxybenzyl)-6-methoxy-2-methyl-1,2,3,4-tetrahydroisoquinolin-7-ol (*rac*-Orientaline) (**2f**)

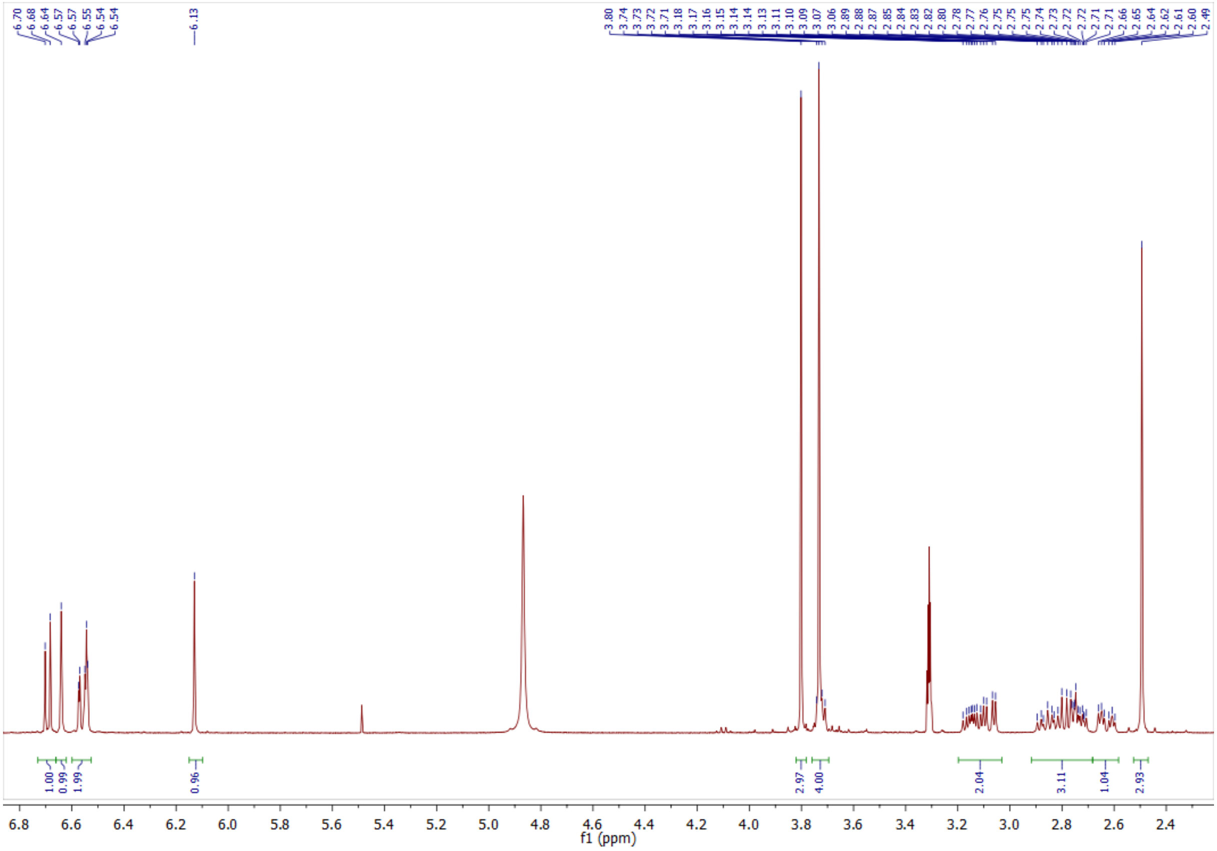

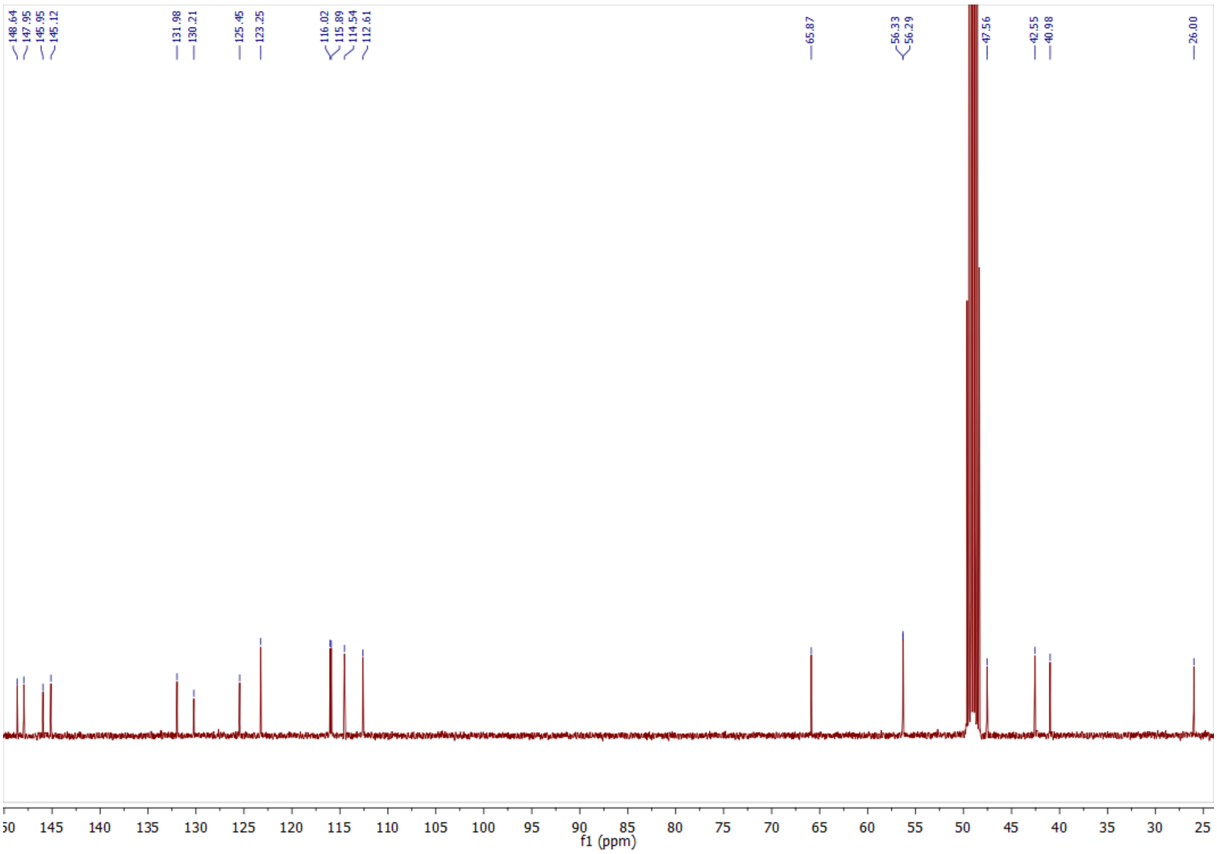

(±)-4-((6-Methyl-5,6,7,8-tetrahydro-[1,3]dioxolo[4,5-g]isoquinolin-5-yl)methyl) phenol (*rac*-Cinnamolaurine) (**3a**)

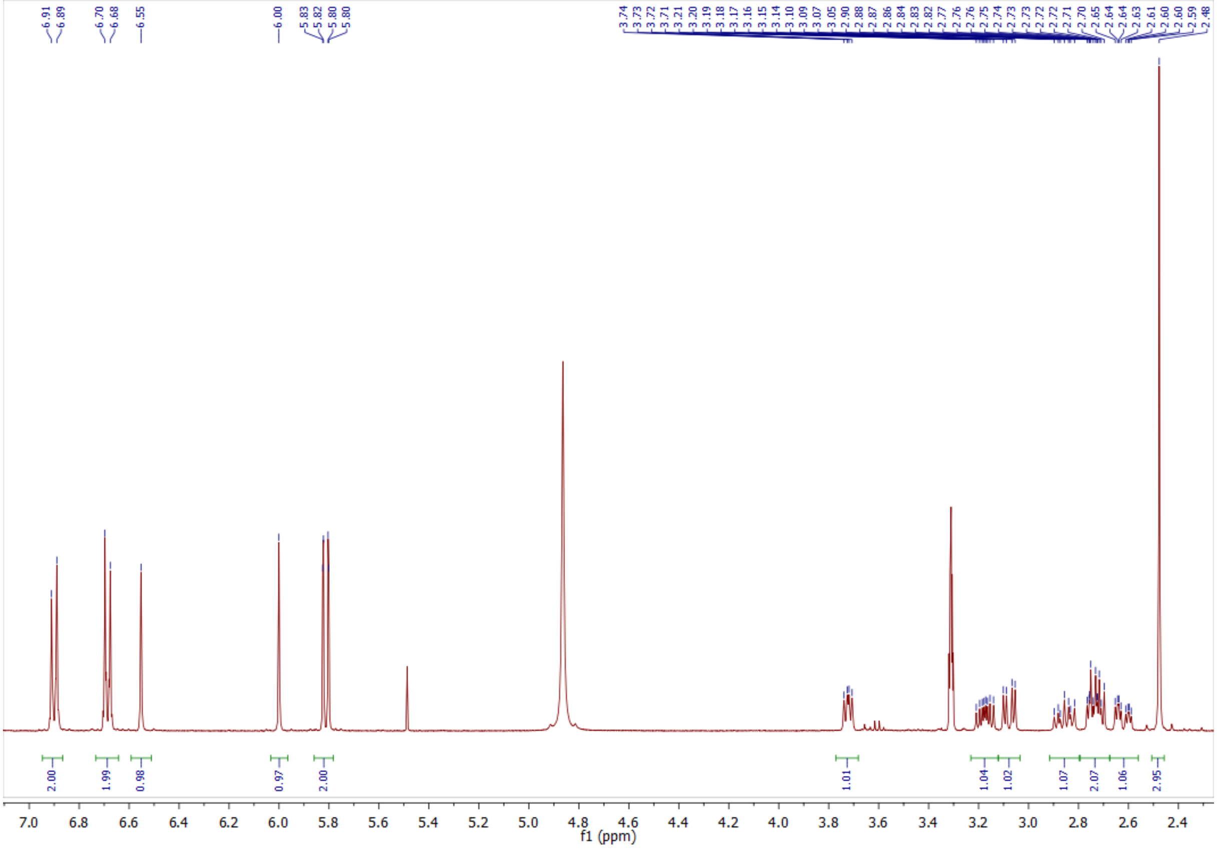

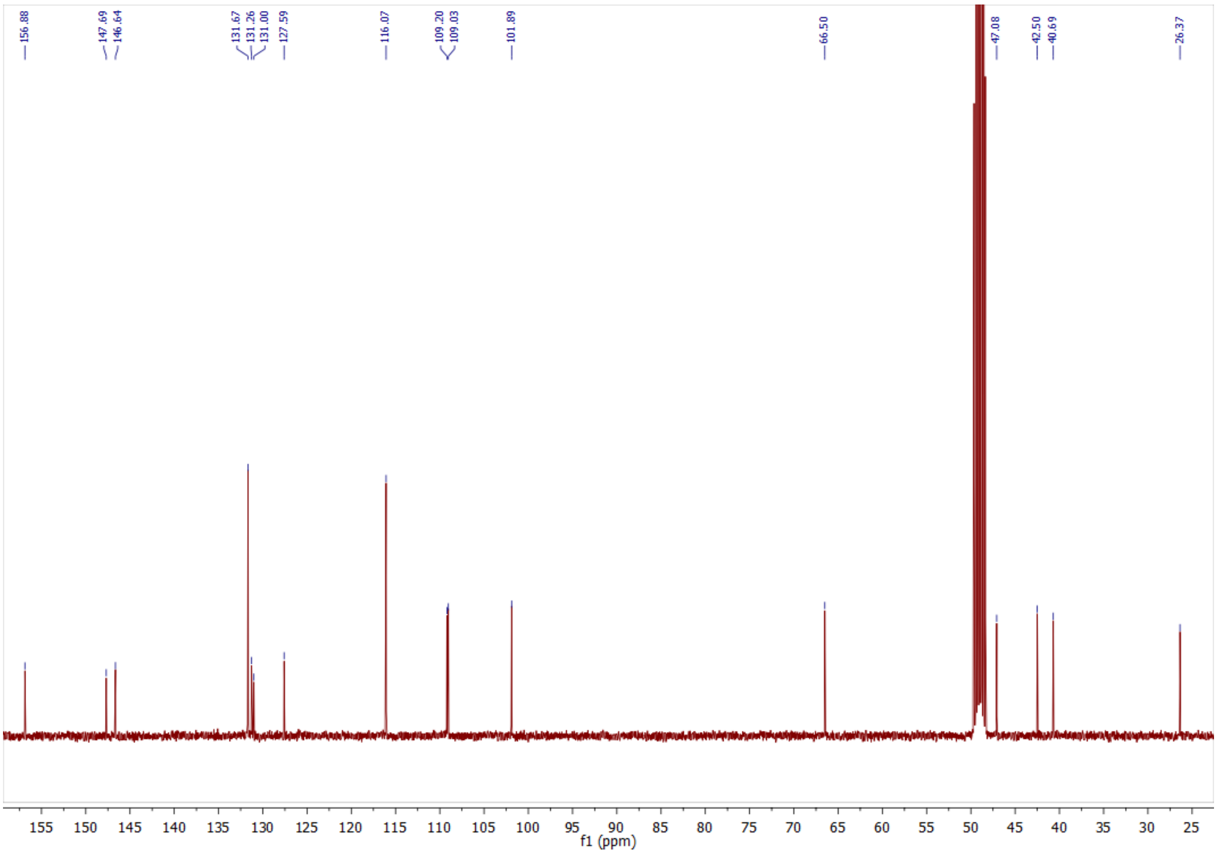

(±)-4-((5,6,7,8-Tetrahydro-[1,3]dioxolo[4,5-g]isoquinolin-5-yl)methyl) phenol (*rac*-Norcinnamolaurine) (**3b**)

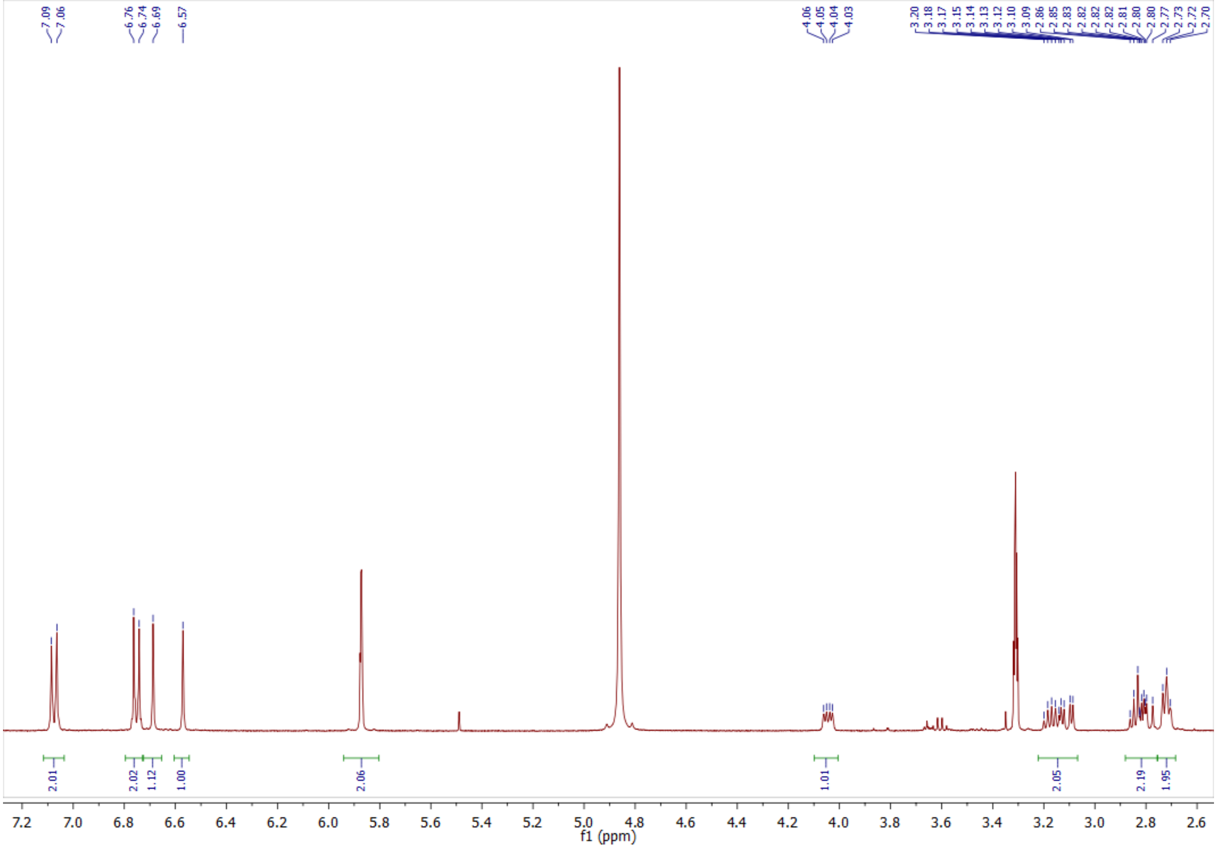

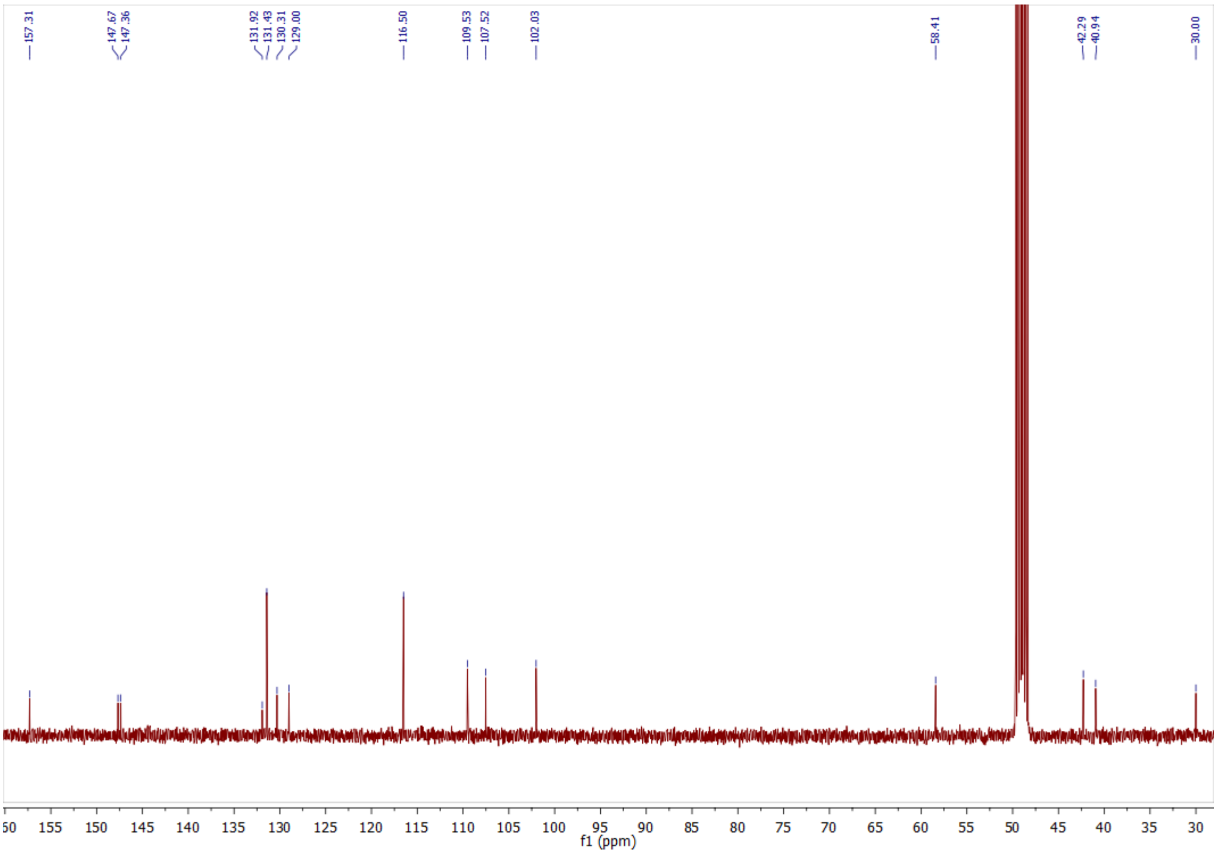

(±)-2-Methoxy-5-((6-methyl-5,6,7,8-tetrahydro-[1,3]dioxolo[4,5-g]isoquinolin-5-yl)methyl) phenol (*rac*-*N*-Demethylphyllocryptine) (**3c**)

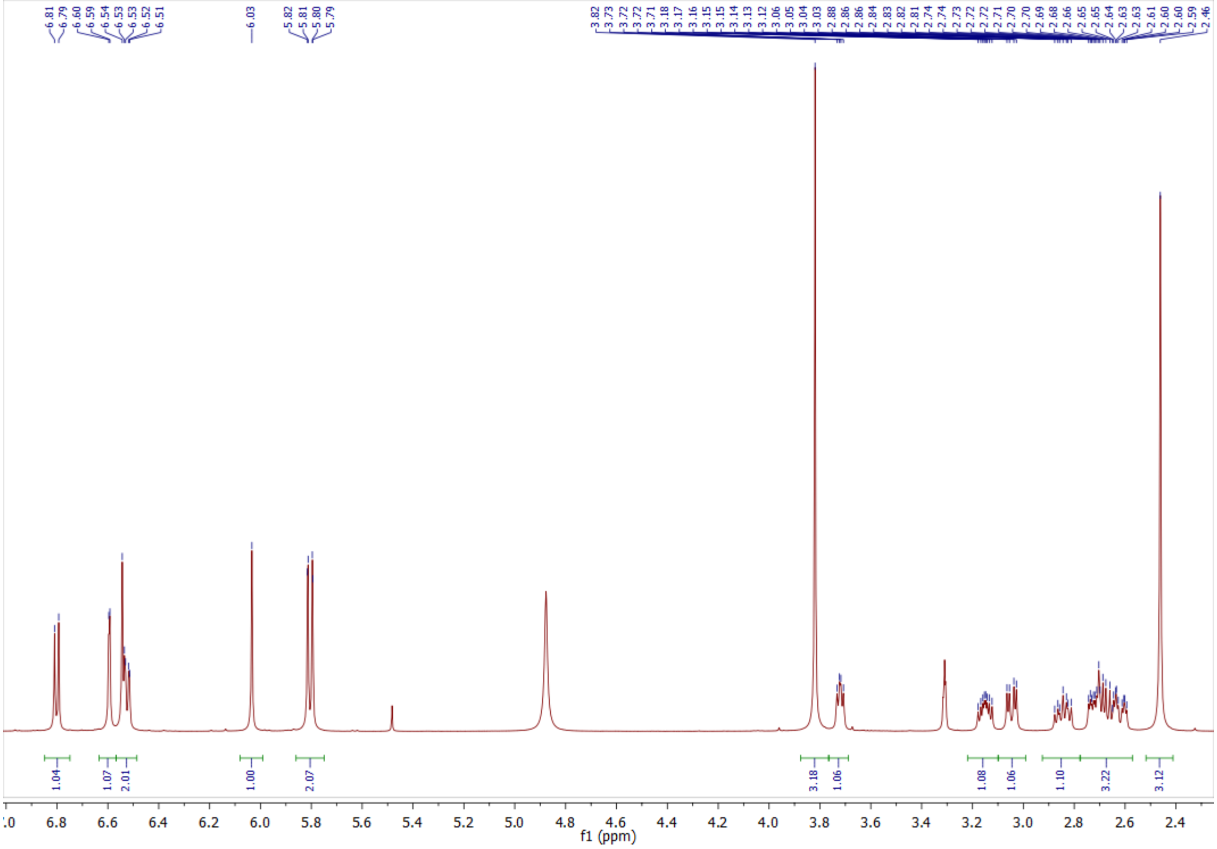

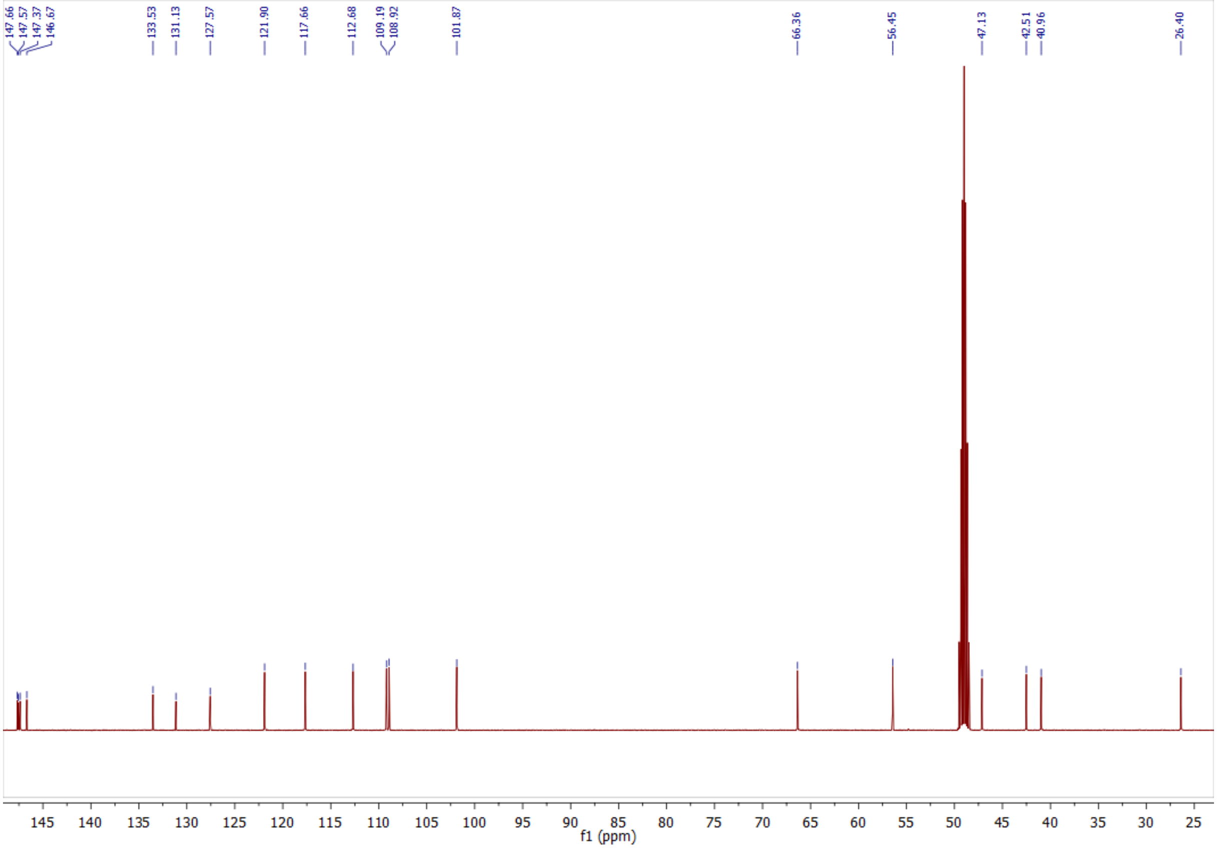

(±)-4-((5,6,7-Trimethoxy-2-methyl-1,2,3,4-tetrahydroisoquinolin-1-yl)methyl) phenol (*rac*-Thalifendlerine) (**4**)

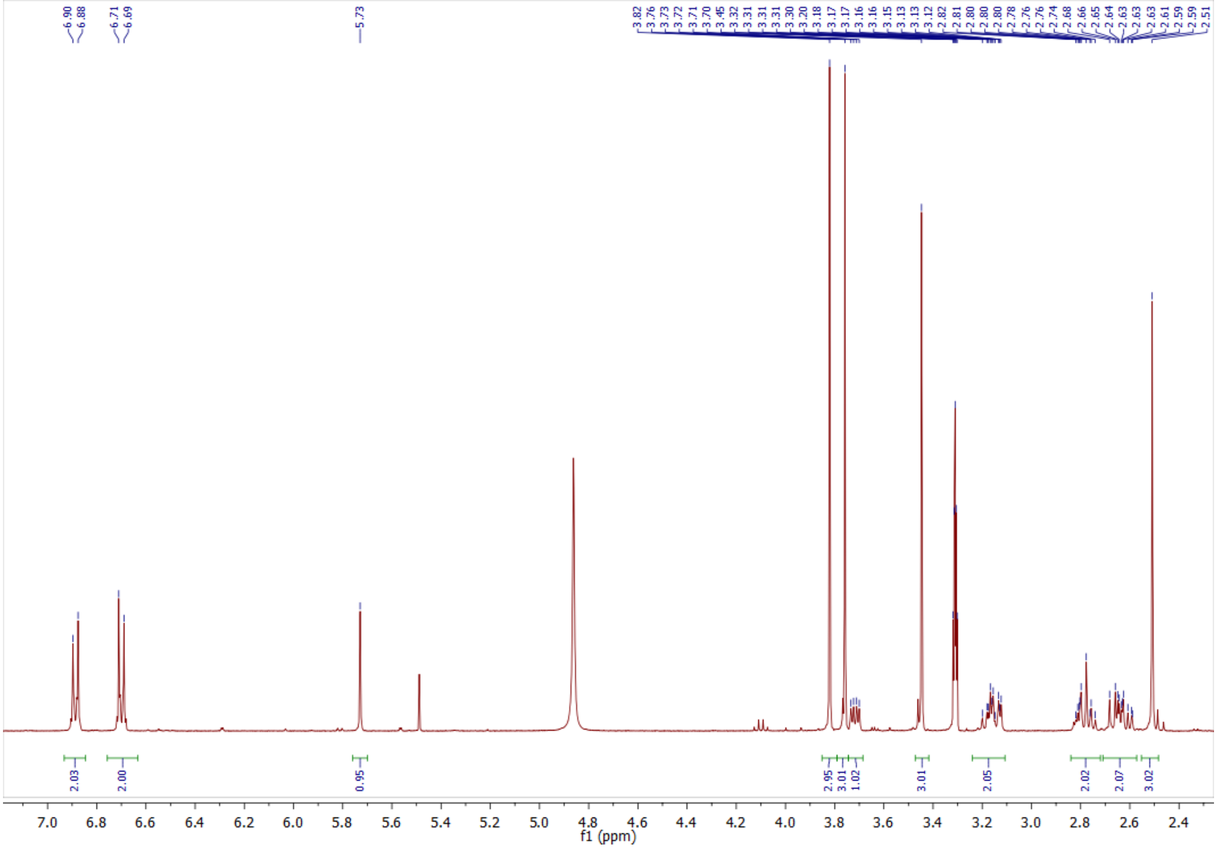

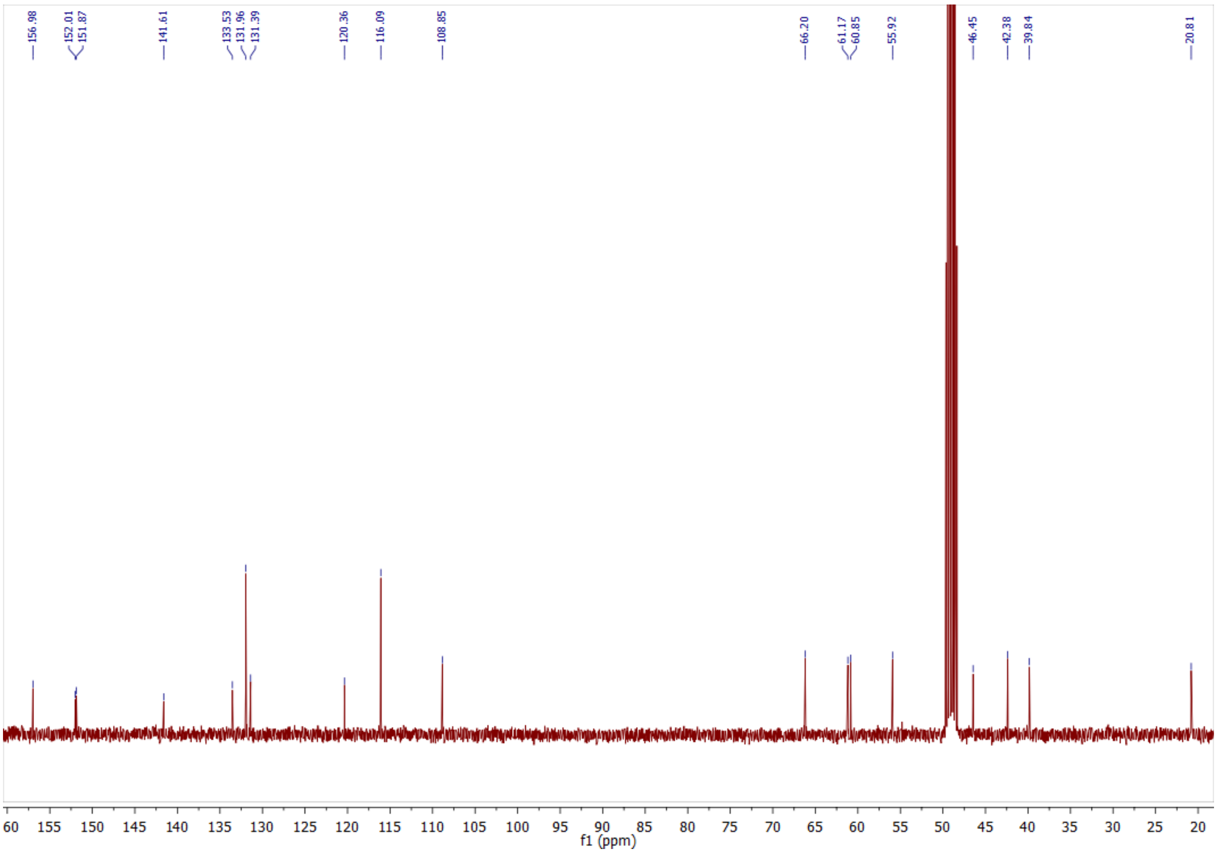

3. Screening for TPC2 modulation

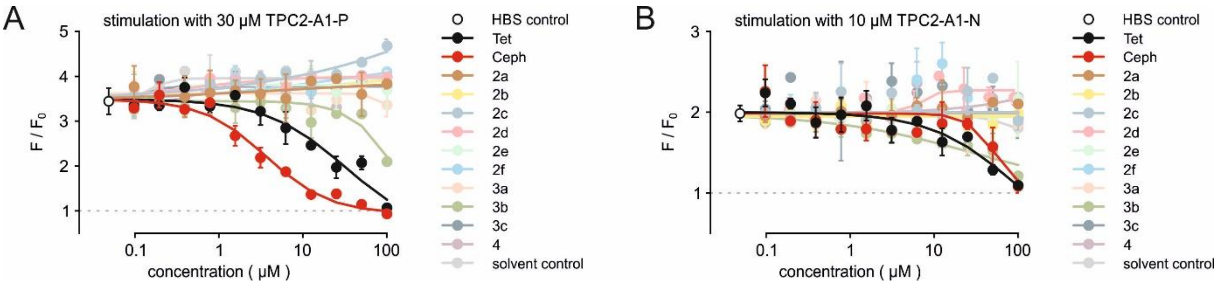

Figure S2 TPC2 modulation. Representative FLIPR analyses and impact of 1-benzyltetrahydroisoquinolines on TPC2 stimulation with TPC2-A1-P- (**A**) or TPC2-A1-N (**B**). Fluo-4 loaded HEK293$_{hTPC2L11A/L12A}$ suspensions were pipetted into multiwell plates and were measured in a FLIPR. After recording of a baseline (F_0), indicated alkaloids, known inhibitors tetrandrine (Tet) and cepharanthine (Ceph) as well as a solvent control were added in a concentration-dependent manner, each concentration in duplicates. After an incubation time of 5 min, followed the addition of the respective TPC2 activator. Ca^{2+} responses in each well were monitored in form of rising fluorescence intensities (F). Concentration-response curves were obtained from n=2 ±SD. Grey dotted line symbolize a complete inhibition of TPC2 (F/F_0=1)

4. Screening for antiproliferative activity of selected alkaloids

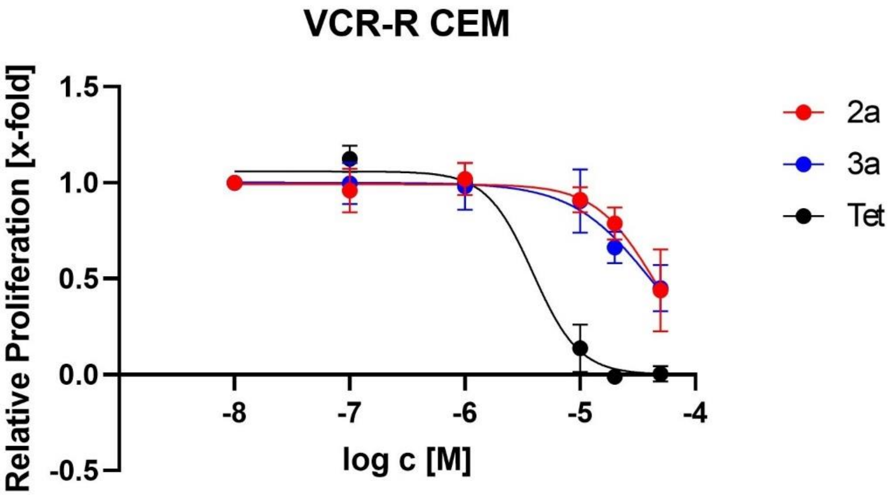

Figure S3: Antiproliferative activity of the benzylisoquinolines **2a** and **3a** in the multidrug-resistant leukemia cell line VCR-R CEM. Values determined by CellTiter

Blue cell viability assay. Tetrandrine (Tet) was used as reference compound. Highest tested concentration was 50 µM (higher concentrations were regarded irrelevant for meaningful evidence on biological activity).

Observed IC_{50} values: tetrandrine: 4 µM, **2a** and **3a**: about 40 µM.

References

1. Müller, M.; Gerndt, S.; Chao, Y. K.; Zisis, T.; Nguyen, O. N. P.; Gerwien, A.; Urban, N.; Müller, C.; Gegenfurtner, F. A.; Geisslinger, F.; Ortler, C.; Chen, C. C.; Zahler, S.; Biel, M.; Schaefer, M.; Grimm, C.; Bracher, F.; Vollmar, A. M.; Bartel, K., *Cell Chem Biol* **2021,** 28, 1119-1131.
2. Chien, C.-W.; Shi, C.; Lin, C.-F.; Ojima, I., *Tetrahedron* **2011,** 67, 6513-6523.
3. Dikusar, E. A.; Kozlov, N. G., *Russian Journal of General Chemistry* **2007,** 77, 905-910.
4. Yang, S. H.; Song, C. H.; Van, H. T.; Park, E.; Khadka, D. B.; Gong, E. Y.; Lee, K.; Cho, W. J., *J Med Chem* **2013,** 56, 3414-8.
5. Awuah, E.; Capretta, A., *J Org Chem* **2010,** 75, 5627-34.
6. Cava, M. P.; Buck, K. T., *Tetrahedron* **1969,** 25, 2795-2805.
7. Kametani, T.; Takahashi, K.; Sugahara, T.; Koizumi, M.; Fukumoto, K., *Journal of the Chemical Society C: Organic* **1971**.
8. Sawyer, J. S.; Narayanan, B. A., *Synthetic Communications* **2006,** 13, 135-138.
